# N4BP1 uses tandem KH domains to associate with EDC4 and mRNA decapping factors in P-bodies

**DOI:** 10.1038/s42003-026-10938-x

**Published:** 2026-09-17

**Authors:** Paweł Piłat, Ankur Garg, Udo Heinemann, Mateusz Wilamowski, Jolanta Jura

**Affiliations:** 1 Jagiellonian University, Faculty of Biochemistry, Biophysics and Biotechnology, Department of General Biochemistry, Krakow, Poland; 2 Jagiellonian University, Doctoral School of Exact and Natural Sciences, Krakow, Poland; 3 Macromolecular Structure and Interaction, Max-Delbrück Center for Molecular Medicine in the Helmholtz Association, Berlin, Germany; 4 Institute for Chemistry and Biochemistry, Freie Universität Berlin, Berlin, Germany; 5 Present Address: Cold Spring Harbor Laboratory, New York, NY USA; 6 Present Address: Howard Hughes Medical Institute, Cold Spring Harbor Laboratory, New York, NY USA

**Keywords:** RNA, X-ray crystallography, RNA decay

## Abstract

Processing bodies (P-bodies) are cytoplasmic, non-membrane-bound structures involved in mRNA decay. EDC4 serves as a key scaffold for the decapping complex within P-bodies. Here, we demonstrate that N4BP1 interacts with EDC4, as well as with DCP1A, DCP2, and XRN1 - key components of 5′-cap hydrolysis. Endogenous N4BP1 colocalizes with EDC4 in P-bodies, requiring both of its KH domains. Structural analysis revealed that N4BP1 contains a type-I KH fold but lacks the canonical GXXG motif required for single-stranded RNA binding. Deletion or mutation of KH domains non-canonical GXXG motifs disrupts the N4BP1-EDC4 complex. N4BP1 reduces HIV-1 transcript levels independently of its P-body localization or association with decapping components, implying the involvement of other host factors in regulating viral mRNAs. Similarly, for N4PB1-dependend negative regulation of endogenous transcripts in HaCaT keratinocytes, EDC4 is not essential. For both HIV-1 and endogenous transcripts, the reduction is associated with the activity of the NYN domain.

## Abstract (web summary)

We show the association of N4BP1 RNase with key components of the decapping complex in P-bodies. We identify the KH domains of N4BP1 as crucial for the interaction and show that N4BP1 suppresses HIV-1 independently of P-bodies recruitment.

## Introduction

NEDD4-binding partner-1 (N4BP1) was first described in 2002 as a protein interactor and monoubiquitylation substrate of Nedd4^1^. Subsequent studies revealed that N4BP1 is an endonuclease containing an NYN domain belonging to the PIN-like (Pil-T amino-terminal) superfamily 3, responsible for RNA degradation^2,3^. The protein also possesses tandem K-homology RNA-binding domains (KHDs), typically involved in RNA target recognition, and a C-terminal ubiquitin-binding domain known as COCUN (Cousin of CUBAN), responsible for the interaction of the protein with ubiquitin^3^.

N4BP1 is inducible by IFN-α and restricts infection of HIV-1 strains in human T-cells and macrophages in an IFN-dependent manner^4^. It has also been shown to directly bind HIV-1 mRNA. In CD4^+^ T cells, which are highly permissive to HIV-1 infection, the level of N4BP1 protein rapidly decreases due to cleavage at Arg509 triggered by MALT1 paracaspase. This degradation of N4BP1 results in HIV-1 reactivation^4^. Importantly, N4BP1’s antiviral activity is not limited to HIV-1: it also inhibits gene expression of *gammaretroviruses* (MLV) and *spumaviruses* (HFV)^4,5^.

Further studies have confirmed that N4BP1 is involved in negative regulation of inflammation. Bone marrow-derived macrophages (BMDMs) from *N4bp1*^*-/-*^ mice exhibited hyper-responsiveness to stimulation of TLR1/2, TLR7, and TLR9 - though not TLR3 and TLR4 - and produced increased levels of cytokines, including IL-6, TNF-α, G-CSF, as well as the chemokines CXCL1, CCL3 and CCL4 compared to wild-type controls^6^. In vitro experiments demonstrated that N4BP1 is cleaved by caspase-8 following stimulation of TLR3 and TLR4. This cleavage was not observed upon treatment of BMDMs with TLR1/2, TLR7, and TLR9 agonizts. Both N4BP1 and caspase-8 were detected in the cytoplasmic fraction, indicating that cleavage occurs in the cytosol^6^. Interestingly, N4BP1 was shown to inhibit NFκB activation downstream of all TLRs except TLR3 and TLR4 by interacting with NEMO through its coil-zipper (COZI) domain. Caspase-8-mediated cleavage of N4BP1 separates the NYN domain, which retains RNase activity and contributes to the antiviral response^7^.

The role of N4BP1 as an immune regulator is also significant in the skin. In vitro studies have shown that N4BP1 regulates keratinocyte proliferation and differentiation, and that N4BP1-deficient mice develop a severe and persistent psoriasis-like disease induced by imiquimod (IMQ). Moreover, due to its high expression in neutrophils, N4BP1 limits their survival in the blood and their infiltration into psoriatic skin by targeting CXCL1, CCL20, and S100A8^8^.

Although the role of N4BP1 in negatively regulating inflammation, as well as its involvement in inhibiting viral RNA replication, is well established, the localization of N4BP1 and its binding partners still requires detailed investigation. In this study, we identified potential partners of N4BP1 and confirmed its association with P-body proteins, including EDC4. Moreover, we present the crystal structure of the tandem N4BP1 K-homology domains (KHDs) and identify these domains as an essential region for the interaction between N4BP1 and EDC4.

## Results

**Fig. 1:**
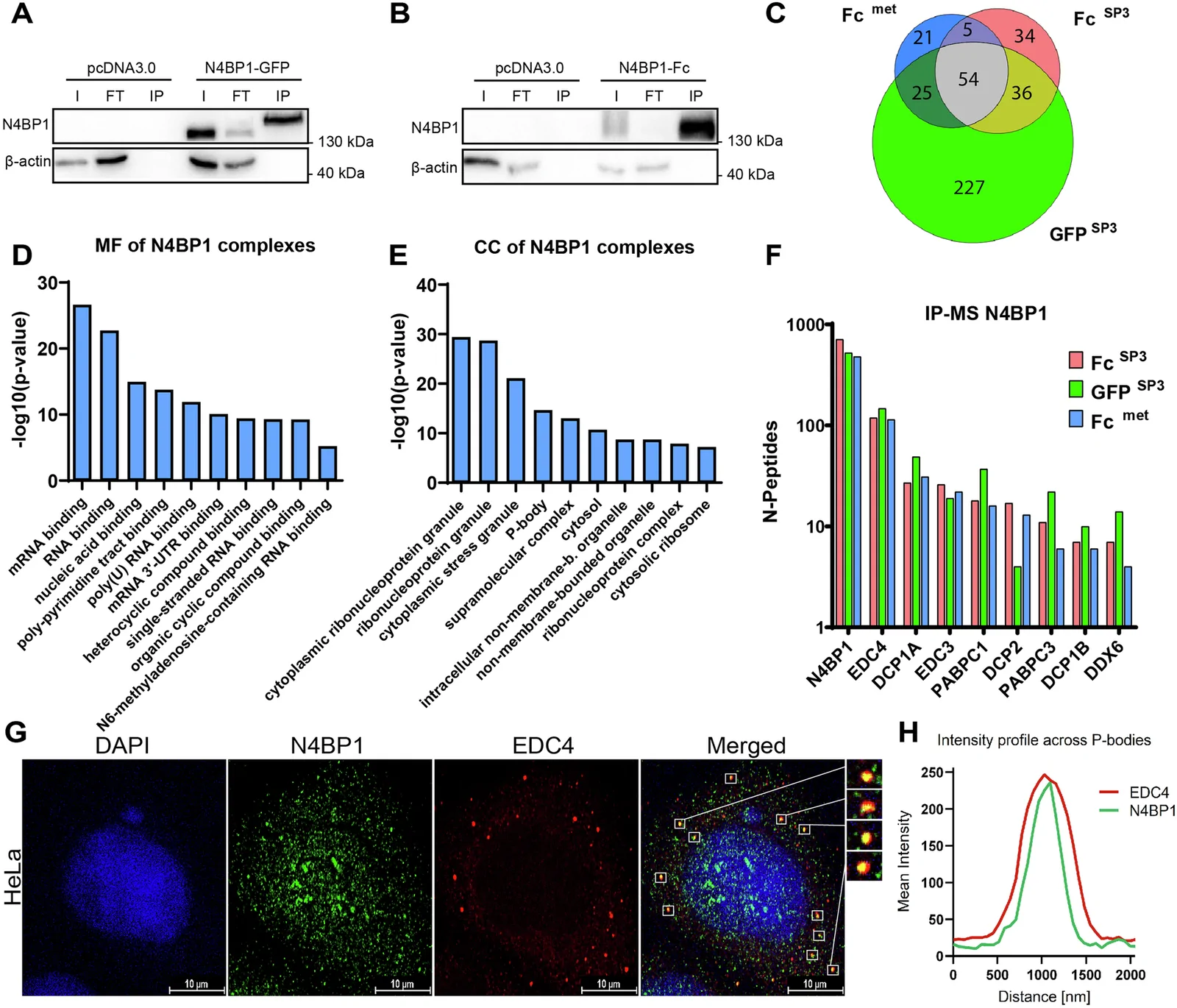
Immunoprecipitation of N4BP1 complexes from HEK293T cells followed by MS analysis. N4BP1 is a novel component of P-bodies. **A** Western blot analysis demonstrates efficient immunoprecipitation of N4BP1-GFP complexes. **B** Western blot analysis confirming successful IP of N4BP1-Fc complexes. **C** Venn diagram illustrates the number of proteins identified by mass spectrometry using different N4BP1 immunoprecipitation approaches (Fc or GFP) and extraction methods (methanol precipitation or SP3-based purification). **D** Gene Ontology (GO) analysis of molecular functions (MF) for proteins identified in N4BP1 complexes, limited to the top ten categories. **E** GO-based analysis of the cellular components (CC) associated with N4BP1-interacting proteins. **F**. IP-MS data representing peptide count for N4BP1-interacting proteins involved in RNA decapping. (Data presented at **D** and **E** were generated using g:Profiler. Statistical significance was assessed using a one‑tailed Fisher’s exact test (cumulative hypergeometric test) with g:SCS correction for multiple testing) **G** Confocal images showing colocalization of endogenous N4BP1 and EDC4 in cytoplasmic granules HeLa cells following immunofluorescent staining. **H** Representative fluorescence intensity profile of colocalizing granules, showing overlapping signals for EDC4 (red) and N4BP1 (green).

Since RNA-binding proteins often function as components of multiprotein complexes to coordinate diverse molecular activities, we hypothesize that the RNase activity of N4BP1 should also be investigated in the context of a multi-subunit RNP complex. In this study, we developed an assay for the efficient identification of molecular complexes interacting with N4BP1. For immunoprecipitation (IP) of N4BP1, its coding sequence was fused at the C-terminus to either the Fc fragment of an immunoglobulin heavy chain or to green fluorescent protein (GFP) (Fig. 1A, B). In the Fc-based approach, the tagged protein was captured directly on a protein G resin. In the GFP-based approach, we used anti-GFP nanobodies covalently coupled to a GFP-Trap resin for affinity purification. These tag-based IP methods circumvent the need for polyclonal antibodies, and thus significantly reduce background signal associated with conventional IP protocols.

The efficiency of N4BP1 IP from HEK293T cells was validated by Western blotting. A substantial reduction in the bait protein signal in the flow-through (FT) fraction following IP confirmed effective binding of tagged N4BP1 to the magnetic beads. Moreover, the elution fraction contained a strong N4BP1 signal with minimal background, as indicated by the absence of non-specific proteins, such as β-actin (Fig. 1A, B). These results demonstrate that our affinity-based workflows successfully isolate N4BP1 with minimal contamination of lysate-derived protein. Importantly, potential artifacts introduced by the molecular tags were mitigated by employing two tagging systems (Fc and GFP).

We next applied immunoprecipitation followed by mass spectrometry analysis (IP-MS) to identify N4BP1-associated multiprotein complexes (Fig. 1C–F). These proteins were isolated using either Fc- or GFP-tagged N4BP1 constructs. Two protein extraction protocols were used for Fc-tagged N4BP1: standard methanol precipitation (N4BP1_Fc met_) and SP3-based purification (N4BP1_Fc SP3_), and one data set against N4BP1 fused to GFP (N4BP1_GFP SP3_). A Venn diagram illustrates relations between the three IP-MS datasets containing proteins that form complexes with N4BP1. Analysis of these datasets revealed a core set of 54 proteins consistently identified across all three conditions (Fig. 1C). Mascot scores, used to evaluate the confidence of protein identification, are provided in Table 1 and Supplementary Data 1. The comparable enrichment levels observed for key N4BP1-associated proteins across the GFP-and Fc-tagged samples (Fig. 1F, Table 1, and Supplementary Data 1) suggest that the fusion tags do not significantly affect N4BP1 function or protein-protein interactions. Together, these data establish a reliable platform for studying N4BP1-associated protein complexes, which warrants further investigation into the functional role of N4BP1 in RNA metabolism.

**Table 1:** List of proteins identified in IP-MS analysis of N4BP1 complex in HEK293T cell line

	FC_SP3	Mascot_score	FC_met	Mascot_score	GFP_SP3	Mascot_score
1	**N4BP1**	**41031**	**N4BP1**	**26252**	**N4BP1**	**30245**
2	IGHG	14665	IGHG	10165	**EDC4**	**8082**
3	**EDC4**	**6618**	**EDC4**	**5889**	HSPA1A	3092
4	IGHG3	4543	IGHG3	4324	HSPA8	2429
5	IGHG2	3967	IGHG2	3769	**DCP1A**	**2337**
6	**DCP1A**	**1483**	IGHG4	2918	HSPA1L	2039
7	SFPQ	1080	**DCP1A**	**1561**	HNRNPM	1992
8	**EDC3**	**1066**	**EDC3**	**942**	**PABPC1**	**1656**
9	**PABPC1**	**763**	TRIM25	855	SFPQ	1600
10	NONO	703	**PABPC1**	**733**	DHX9	1498
11	**DCP2**	**652**	NONO	670	NONO	1330
12	IGF2BP1	610	**DCP2**	**632**	IGF2BP1	1091
13	TRIM25	583	HSPA2	628	HSPA2	1089
14	RO60	564	IGF2BP1	572	DDX17	980
15	**YBX1**	**475**	SFPQ	482	HNRNPA1	979
16	**PABPC3**	**469**	RO60	447	DDX3X	975
17	H1-3	410	RBM14	408	RPS27A	937
18	HSPD1	386	**PABPC4**	**382**	DDX5	933
19	**DCP1B**	**341**	TBK1	298	NPM1	872
20	RBM14	303	IGF2BP3	292	**PABPC3**	**862**
21	H2BU1	299	**DCP1B**	**289**	TBK1	849
22	**DDX6**	**296**	**PABPC3**	**276**	**EDC3**	**823**
23	TBK1	282	SLC25A6	246	DDX3Y	735
24	SSB	251	RPLP2	230	RPS3	735
25	PGAM5	232	SLC25A5	228	HNRNPA2B1	724
26	SLC25A6	226	HNRNPC	224	RPLP0	708
27	AZI2	223	**DDX6**	**187**	RBM14	689
28	EEF1A2	207	AZI2	179	HSPA6	680
29	**PABPC4**	**187**	HNRNPM	169	HNRNPA1L2	669
30	HNRNPA1L2	179	IGLL5	156	**PABPC4**	**659**
31	YBX3	170	LSM14A	149	RPS19	614
32	PRDX4	168	SLC25A4	148	DSG1	610
33	RBMX	163	IGF2BP2	146	**DDX6**	**592**
34	RPLP2	152	HNRNPA3	139	FLG2	566
35	SLC25A4	151	HNRNPCL2	138	HSPA5	558
36	RPS3	148	MIB1	122	NCL	540
37	HNRNPC	143	PGAM5	122	RPL12	522
38	YWHAQ	134	CLTC	121	ALB	520
39	BAG2	130	PABPC1L	120	HSPA7	476
40	TUBA4A	128	SSB	120	HNRNPA3	462
41	PABPC1L	121	ELAVL1	115	HNRNPA0	455
42	RPL13	121	RPS14	114	AZI2	434
43	HNRNPK	121	**YBX1**	**113**	**DCP1B**	**427**

Protein names are printed bold for the set of proteins engaged in formation of the decapping complex.

IP-MS analysis of methanol-purified N4BP1_Fc_ complexes (N4BP1_Fc met_) identified 105 N4BP1-interacting proteins, whereas purification of N4BP1_Fc_ complexes using the SP3 column (N4BP1_Fc SP3_) followed by IP-MS yielded 129 potential interactors. Proteins/peptides derived from the bait, including N4BP1 itself and IGHG sequences from the Fc tag, were excluded from downstream analysis. In the third IP-MS dataset, based on GFP-tagged N4BP1 purified using the SP3 method (N4BP1_GFP SP3_), we identified 342 proteins. Notably, high-confidence N4BP1 interactors (based on peptide counts and Mascot scores) were consistently present across three datasets. However, the N4BP1_GFP SP3_ dataset also included a broader set of cytoplasmic proteins, which may reflect non-specific binding to the GFP-Trap beads. Importantly, these background proteins were identified with low confidence, as indicated by low Mascot scores (Table 1 and Supplementary Data 1).

### N4BP1 RNase localizes to P-bodies

Two major types of cytoplasmic granules involved in mRNA turnover are processing bodies (P-bodies) and stress granules (SGs)^9,10^. Gene Ontology (GO) analysis of molecular functions of N4BP1-interacting proteins revealed their involvement in key processes such as mRNA binding, transcript decapping, antiviral response, poly-pyrimidine tract binding, single-stranded RNA binding, or interaction with various non-coding RNAs (Fig. 1D, Table 1, and Supplementary data 2). GO cellular component (GO CC) analysis based on IP-MS indicated that N4BP1-containing complexes are predominantly localized in non-membrane-bound cytoplasmic organelles, including P-bodies and SGs (Fig. 1E and Supplementary Data 2). The primary GO CC term classifying these proteins is “cytoplasmatic ribonucleoprotein granules”. Notably, we identified 12 proteins associated with P-bodies within the N4BP1 complex: EDC4, EDC3, DCP2, DCP1A, DCP1B, DDX6, IGF2BP1, IGF2BP2, IGF2BP3, ELAVL1, PATL1 and CAPRIN1 (Fig. 1F and Supplementary Data 2). These findings strongly link the molecular functions of N4BP1-associated proteins to the processing of single-stranded RNAs and mRNA turnover.

According to the parameters used in Mascot analysis, one of the major interaction partners of N4BP1 identified by IP-MS was enhancer of mRNA-decapping protein 4 (EDC4) (Fig. 1F and Table 1). This interaction is particularly noteworthy, as EDC4 serves as a scaffold protein essential for the assembly of the mRNA-decapping complex within P-bodies^11^. The canonical core of the decapping complex contains EDC4 and EDC3—both of which were identified in our IP-MS analysis (Fig. 1F and Table 1)—which function as molecular scaffolds for the recruitment of various decapping cofactors: mRNA-decapping enzyme 1A (DCP1A) and DCP1B, as well as the RNA cap hydrolase DCP2 (Fig. 1F). The co-immunoprecipitation of EDC3, EDC4, DCP1A and DCP2 with N4BP1 suggests that N4BP1 may be localized to P-bodies. To ensure that this observation was not an artifact of N4BP1 overexpression, we performed immunofluorescent staining of endogenous N4BP1 and EDC4. Confocal microscopy revealed the colocalization of the two proteins in small cytoplasmic granules (Fig. 1G, H). The colocalization of endogenous N4BP1 and EDC4 supports the existence of a functional association within P-bodies, potentially implicating N4BP1 in RNA processing or decay pathways.

We also identified a group of cytoplasmic poly(A)-binding proteins (PABPs) including PABPC1, PABPC3 and PABPC4, which bind to poly(A) tails of mRNA in the cytoplasm (Fig. 1F). PABPC1 is known to physically interact with the eIF4F translation initiation complex associated with the 5′ cap structure of mRNA^12^, thereby promoting mRNA circularization and facilitating translation. However, evidence also suggests that PABPs can inhibit decapping by directly and specifically associating with the 5′ end of capped RNAs. In turn, the affinity of PABPs for the cap structure can be reduced by enhanced DCP2 decapping activity^13^. Although association of PABPs with stress granules has been well documented, proteomic analysis of P-bodies in human T lymphocytes has also revealed the presence of PABPC1 in P-bodies^14^. Thus, the identification of PABPs among the top candidate interactors in the N4BP1 IP-MS datasets further supports the idea that N4BP1 protein localizes to P-bodies.

### N4BP1 RNase is a component of the mRNA-decapping complex

**Fig. 2:**
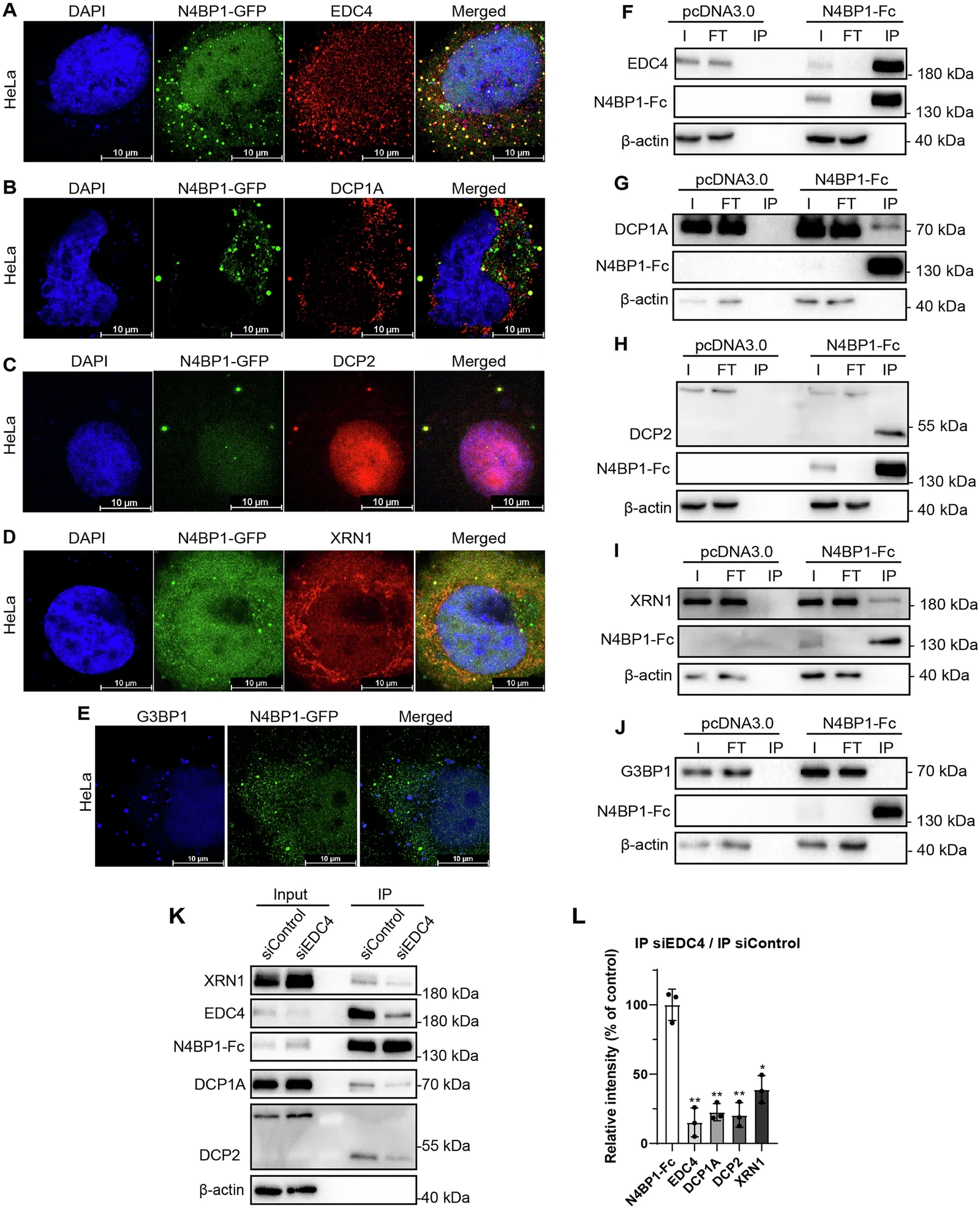
N4BP1 interacts and colocalizes with P-body markers, but not with stress granule (SGs) markers. **A–D** Confocal immunofluorescence microscopy showing colocalization of N4BP1-GFP with the indicated P-body markers in HeLa cells: **A** EDC4, **B** DCP1A, **C** DCP2, **D** XRN1. N4BP1-GFP localizes to cytoplasmic granules that overlap with these canonical P-body proteins. **E** Immunofluorescence staining of G3BP1 in N4BP1-GFP-expressing HeLa cells. **F–I** Co-immunoprecipitation (co-IP) of N4BP1-Fc with endogenous P-body proteins from HEK293T cells: **F** EDC4, **G** DCP1A, **H** DCP2, **I** XRN1. These interactions confirm N4BP1’s association with core components of the 5′ mRNA decapping machinery. **J** Co-IP of N4BP1-Fc shows that G3BP1 does not co-immunoprecipitate with N4BP1, supporting the conclusion that N4BP1 is not a component of stress granules. Input and flow-through represent 2% of total lysate, IP represents 30% of total elution volume. **K** Co-IP of N4BP1-Fc with P-body components (EDC4, DCP1A, DCP2 and XRN1) in HEK293T cells transfected with EDC4-specific siRNA or control siRNA. **L** Densitometric analysis of Western Blot signals from (**K**), shown as a percentage of the control (N4BP1-Fc co-IP with control siRNA). Data are presented as mean ± SD from 3 independent experiments. Two-sided Brown-Forsythe and Welch’s ANOVA was used for statistical analysis: *F*(4, 8,98) = 40,38, *P* < 0,0001. EDC4 vs N4BP1-Fc *t*(3,11) = 10,38, *P* = 0,0056, DCP1A vs N4BP1-Fc *t*(3,97) = 9,53, *P* = 0,0022, DCP2 vs N4BP1-Fc *t*(3,78) = 9,58, *P* = 0,0021, XRN1 vs N4BP1-Fc *t*(3,94) = 7,00, *P* = 0,007.

To validate the IP-MS results of N4BP1 complexes, we performed immunofluorescence staining of selected mRNA-decapping proteins that also serve as P-body markers (EDC4, DCP1A, DCP2, XRN1), as well as the stress granule marker G3BP1 (stress granule assembly factor 1) in HeLa cells transfected with an N4BP1-GFP plasmid. Our results show that N4BP1-GFP colocalized with endogenous EDC4 (Fig. 2A), DCP1A (Fig. 2B), DCP2 (Fig. 2C), and XRN1 (Fig. 2D). Conversely, we did not observe evidence for an overlapping signal of N4BP1-GFP with the analyzed endogenous SG marker G3BP1 (Fig. 2E), suggesting that N4BP1 does not localize in stress granules.

Overall, more than 60% of N4BP1-GFP or EDC4-positive granules were found to colocalize (Fig. S1A–C), supporting a spatial association between these two proteins. Although P-bodies and stress granules are often in spatiotemporal proximity and may exchange proteins or RNA components, our data suggest a specific localization to P-bodies. Importantly, the GFP-tagged variant of N4BP1 displayed a distinct subcellular localization pattern compared to GFP alone upon transfection into HeLa cells (Fig. S2A, B). Whereas GFP alone was diffusely distributed within the nucleus and partially accumulated in nuclear granules (Fig. S2B), N4BP1-GFP localized predominantly in cytoplasmic granules. The granule formation by N4BP1 was not only restricted to HeLa cells: similar colocalization patterns were observed in additional cell lines, including HaCaT and HEK293T (Fig. S2C, D). These control experiments confirm that N4BP1 specifically localizes to cytoplasmic P-bodies.

To validate the association of N4BP1 with components of the 5′ mRNA decapping complex, we performed co-immunoprecipitation followed by Western blot analysis using transfected HEK293T cells. Western blotting of N4BP1-Fc IP eluates confirmed association with key decapping factors, including EDC4 (Fig. 2F), DCP1A (Fig. 2G), DCP2 (Fig. 2H), and the 5′-to-3′ exonuclease XRN1 (Fig. 2I), but not with the stress granule marker G3BP1 (Fig. 2J). These findings support the conclusion that N4BP1 associates specifically with components of the decapping complex rather than with stress granules. Interestingly, a lower molecular weight band of DCP2 was observed in the IP fraction compared to the input and flow-through (Fig. 2H), which may reflect the removal of post-translational modifications during immunoprecipitation.

To confirm whether EDC4 is necessary for the interaction of N4BP1 with DCP1A, DCP2, and XRN1, we silenced the EDC4 gene using specific siRNA and analyzed the levels of the decapping components via immunoprecipitation. Both DCP proteins and XRN1 were markedly reduced in the IP samples (Fig. 2K, L), suggesting that EDC4 mediates the interaction between N4BP1 and these decay factors.

### The KH domain is mandatory for N4BP1 interaction with EDC4

**Fig. 3:**
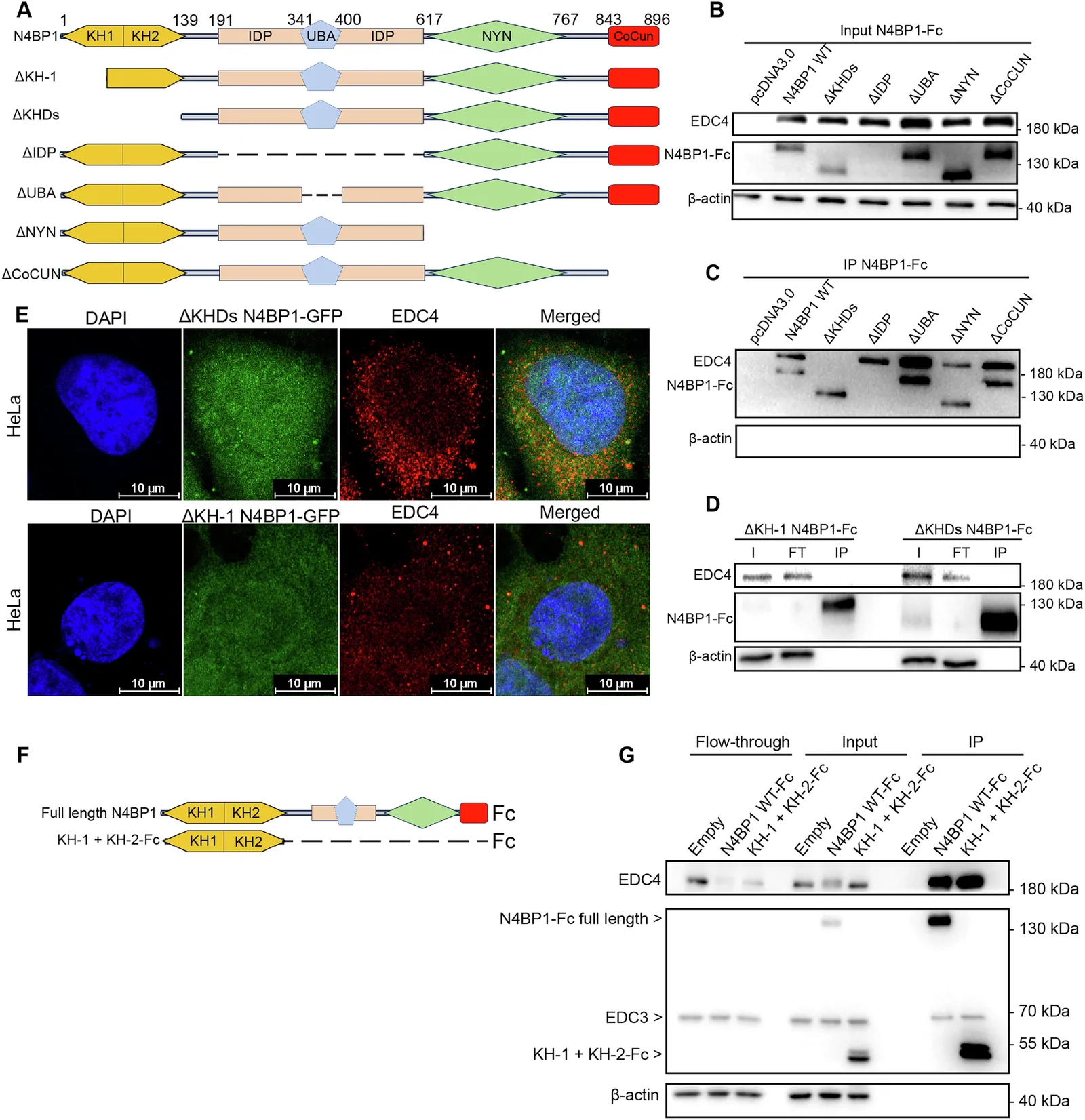
The KH domains in N4BP1 are essential for interaction with EDC4. **A** Schematic representation of the domain structure of human N4BP1 and its domain deletion variants used in the study. **B** Western blot of input fractions from co-immunoprecipitation (co-IP) experiments in HEK293T cells co-transfected with wild-type EDC4 and various N4BP1-Fc deletion constructs. Input corresponds to 2% of total lysate volume. **C** Western blot of co-IP fractions showing the interaction of wild-type EDC4 with N4BP1-Fc constructs carrying deletions of specific domains. IP corresponds to 30% of total elution volume. **D** Co-IP analysis of N4BP1-Fc variants lacking the KH-1 domain (ΔKH-1) or both KH domains (ΔKHDs) with wild-type EDC4 in HEK293T cells. Input and flow-through correspond to 2% of total lysate volume; IP represents 30% of total elution volume. **E** Immunofluorescence staining of EDC4 in HeLa cells transfected with GFP-tagged ΔKHDs or ΔKH-1 N4BP1 constructs. Both deletions result in diffuse N4BP1-GFP localization and loss of granule colocalization with EDC4, indicating impaired P-body targeting. **F** Schematic representation of full-length and KH-1 + KH-2 N4BP1-Fc mutant. **G** Western blot analysis of co-immunoprecipitation of N4BP1 and P-bodies components using KH-1 + KH-2 only N4BP1-Fc variant.

To determine which domain of N4BP1 is crucial for the interaction with EDC4, a major  scaffold of the 5′ mRNA decapping complex, we generated several genetic constructs coding for N4BP1-Fc, each carrying a truncation of a respective domain or fragment (Fig. 3A). EDC4 emerged as the top candidate protein interacting with N4BP1 in IP-MS analysis (Fig. 1F and Table 1). Therefore, we employed EDC4 in co-IP and Western blot analyses using various N4BP1 constructs. Plasmids with individual N4BP1 domain deletions were co-transfected with EDC4 into HEK293T cells, followed by co-IP and Western blotting. Transfection efficiency was high, and EDC4 was abundantly expressed in all cell lysates (Fig. 3B). Our studies revealed that deletions of most of the N4BP1 domains did not disrupt its interaction with EDC4 (Fig. 3C). Specifically, removal of the (i) central intrinsically disordered part (IDP), (ii) ribonuclease NYN domain, (iii) ubiquitin-associated domain (UBA), or (iv) CoCUN domain did not impair complex formation. It is worth noting that the anti-N4BP1 antibody used in Western blotting recognizes an N-terminal region of N4BP1 (amino acids 250-300); therefore, truncated constructs lacking this region do not produce a corresponding band. Nonetheless, for the ΔIDP-N4BP1 variant, we observed a strong EDC4 signal in the co-IP, indicating successful complex formation despite the absence of an N4BP1 band in the Western blot (Fig. 3B, C). This confirms that the ΔIDP-N4BP1 construct is present in the immunoprecipitated samples.

Next, we found that deletion of the KH domains from N4BP1 (ΔKHDs-N4BP1-Fc variant) abolished EDC4 co-IP with N4BP1 (Fig. 3C), suggesting that these domains are essential for the interaction. To further dissect the contribution of the KH domains, we generated an N4BP1 construct lacking a smaller N-terminal peptide comprising residues 1-57 (ΔKH-1-N4BP1-Fc). This truncation also disrupted complex formation with EDC4, as no interaction was observed (Fig. 3D). To complement the co-IP results, we performed colocalization studies using N4BP1-GFP constructs with deletion of either ΔKH-1 (residues 1-57) or both ΔKH domains (KH-1 and KH-2). In contrast to the punctate granule formation seen with the wild-type N4BP1, both ΔKH-1(1–57) and ΔKHDs N4BP1-GFP variants displayed diffuse cytoplasmic fluorescence without significant granule formation. Notably, the EDC4-positive granules did not colocalize with either deletion variant (Fig. 3E).

To further determine whether the tandem KH domains are sufficient for EDC4 binding, we performed pull-down analyses using constructs expressing either the tandem KH domains or the individual KH-1 and KH-2 domains. These experiments demonstrated that the tandem KH domains are sufficient for association with EDC4 (Fig. 3F, G and Supplementary Fig. S3). Moreover, a substantial fraction of endogenous EDC4 was recovered at the same level from cells ectopically expressing either full-length N4BP1 or the tandem KH domains (Fig. 3G and Fig. S4C). This indicates efficient capture of endogenous EDC4 by the overexpressed tandem KH domains of N4BP1.

This observation indicates that deletion of KH domains impairs both the subcellular localization of N4BP1 to P-bodies and its ability to interact with EDC4. Taken together, our results identify the N-terminal KH domains of N4BP1 as essential for its localization to P-bodies and for its interaction with the decapping scaffold protein EDC4.

### The structure of the N4BP1 tandem KH domains

To delineate the molecular architecture of the N4BP1 KHDs, we aimed to solve the crystal structure of the N4BP1 KHDs. We designed and purified different KH-domain variants with progressively longer C-termini (Fig. S4A, B). Furthermore, to understand the thermodynamic properties of the KH domain of N4BP1, we performed thermal melting experiments for different monomeric tandem-KH domain proteins. The N4BP1 tandem KHD^8-194^, KHD^8-224^ and KHD^8-394^ variants showed single melting transitions at 39 °C, 40.5 °C and 42.5 °C, respectively (Fig. S4C), suggesting that a globular fold exists in all three purified tandem KHDs. Next, to understand the stability of the KHD^8-194^, we performed limited proteolysis experiments. KHD^8-194^ yielded a slightly smaller protein species when incubated with trypsin and thermolysin for 2 h (Fig. S5A, B), whereas chymotrypsin treatment at 1:10,000 w/w ratio showed no observable degradation in KHD^8-194^ (Figs. S4D, 5C). Interestingly, both KHD^8-224^ and KHD^8-394^ generated a stable ~23 kDa protein species upon protease treatment (Fig. S5D–I) but failed to yield high-quality crystals.

**Fig. 4:**
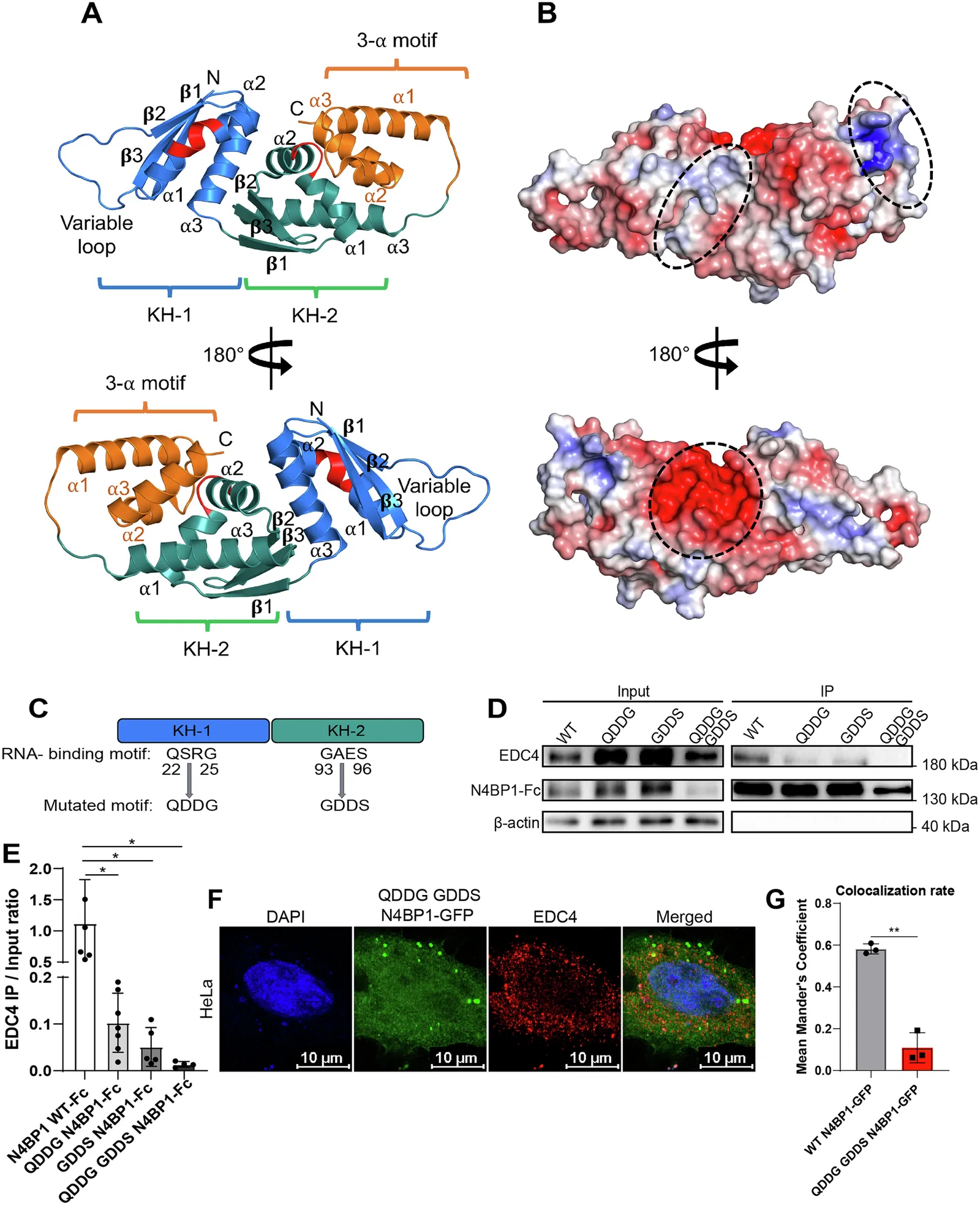
Structure of the human N4BP1 KHD8-194. **A** Cartoon representation of the KHD^8-194^ structure in two orientations. KHD^8-194^ harbors two KH domains in tandem (colored blue and teal, respectively) followed by a short motif with three helices (3-α motif colored orange). Both KH domains have typical type-1 βααββα topology. **B** Electrostatic surface of KHD^8-194^ in two orientations. KHD^8-194^ exhibits few positively charged patches and one deeply negatively charged pocket (highlighted with black ellipse) on its surface. The electrostatic surface potential is displayed in a range from -5 (red) to +5 kT/e (blue). **C** KH-1 and KH-2 RNA-binding motifs with introduced mutations weakening the interaction with RNA. **D** Co-IP of WT N4BP1-Fc and KH domain mutant variants with WT EDC4 in HEK293T cells, Input – 5% of total lysate volume, IP – 30% of total elution volume. **E** Densitometric results of Western Blot analysis are shown on Fig. 4D. Values presented are mean ± SD counted as EDC IP/Input ratio. Statistical analysis was performed using two-tailed Welch’s t-test by comparing each data set with control (N4BP1 WT-Fc). QDDG vs N4BP1 WT-Fc *t*(5,07) = 3,54, *P* = 0,0163, GDDS vs N4BP1 WT-Fc *t*(5,04) = 3,72, *P* = 0,0135, QDDG GDDS vs N4BP1 WT-Fc *t*(5,00) = 3,86, *P* = 0,0119. The exact number of replicates for each sample: N4BP1 WT-Fc *n* = 6, QDDG *n* = 7, GDDS *n* = 5, QDDG GDDS *n* = 4. **F** Immunofluorescent staining of EDC4 in HeLa cells expressing QDDG GDDS N4BP1-GFP mutant. **G** Colocalization rate of WT N4BP1-GFP/QDDG GDDS N4BP1-GFP and EDC4 calculated as Mander’s overlap coefficient (green overlapping red). Data was generated from 3 different images per condition. Data presented are mean ± SD. Two-tailed Welch’s t-test was used for statistical analysis, *t* (2,46) = 10,76, *P* = 0,0004.

Guided by the limited proteolysis experiment, we crystallized N4BP1 KHD^8-194^ with in situ proteolysis, adding chymotrypsin in 1:10,000 w/w ratio to the protein before setting up crystallization. Initial crystallization trials resulted in protein crystals within 3–4 days at 4 °C, while Se-Met derivative crystals appeared within 6–7 days. The N4BP1 KHD^8-194^ crystal diffracted to 1.88 Å and belonged to space group P6_2_ with one molecule in the asymmetric unit. For KHD^8-194^ a structure model was built into unambiguous electron density, except for the loops corresponding to aa 42–47 and aa 144–49, which showed lower-quality electron density but could still be modeled. The structure model also contains an extra Gly-Ser dipeptide at the N-terminus, which remained after TEV-site cleavage. Both individual KH domains of N4BP1 (KH-1^8–75^ and KH-2^77-144^) adopt a typical type-I KH βααββα topology with an antiparallel sheet arranged against three helices, and assembling into a globular fold (Fig. 4A). Interestingly, the C-terminal region of the protein (aa 149–193) adopts a triple-helical bundle (3α motif) and folds on top of the KH-2 domain further stabilizing the structure (Fig. 4A). The KHD^8-194^ tandem domain is decorated with electrostatically charged patches on its surface. A deep negatively charged surface is observed around the α2-α3 helices of KH-2, which is placed between the 3α motif and the KH-1 α2-α3 helices. Slightly positive patches are observed on KHD^8-194^ surface as well (Fig. 4B).

KH domains typically bind target RNA through an invariant GXXG motif located between the α1-α2 helices, as well as a variable loop between the β2-β3 strands^15^. Superimposing the N4BP1 KH domains with the NOVA1/2 KH domains^15,16^ revealed that the overall fold of the N4BP1 KHDs is typical, but the GXXG motif is mutated to QSRG in N4BP1 KH-1 and to GAES in KH-2 (Fig. S6A). Also, in contrast to the NOVA1/2 KH domains, which display a deep positively charged region on the surface for RNA binding (Fig. S6B, C), both N4BP1 KH domains lack a corresponding positively charged surface (Fig. S6D, E).

To investigate whether the changes in the KH motifs in N4BP1 affect its protein-protein interaction with EDC4, we mutated the QSRG (KH-1) and GAES (KH-2) motifs to QDDG and GDDS, respectively (Fig. 4C). Next, we performed IP using wild-type N4BP1-Fc or its mutant forms containing the QDDG and GDDS substitutions, followed by Western blot analysis of the eluted complexes. We observed that WT-N4BP1 co-eluted with EDC4, whereas the KHD mutants failed (Fig. 4D, E). Interestingly, the QDDG/GDDS mutant did not clearly colocalize with EDC4, despite forming granule-like structures in the cytoplasm (Fig. 4F). Quantitative analysis showed that colocalization of WT N4BP1-GFP with endogenous EDC4 was approximately five times higher than that of the mutant variant (Fig. 4G). Although substitution of the two internal amino acid residues with aspartic acid is not expected to disrupt the overall fold of the protein, we observed a decreased stability of the QDDG/GDDS N4BP1 mutant, likely due to enhanced proteasomal degradation compared to WT N4BP1 (Fig. S7A, B). Together, these results strongly suggest that the N4BP1 KHDs interact with EDC4 via their non-canonical GXXG-like motifs, and that alterations in these sequences disrupt this interaction.

### RNA does not contribute to the stabilization of the interaction between N4BP1 and EDC4

**Fig. 5:**
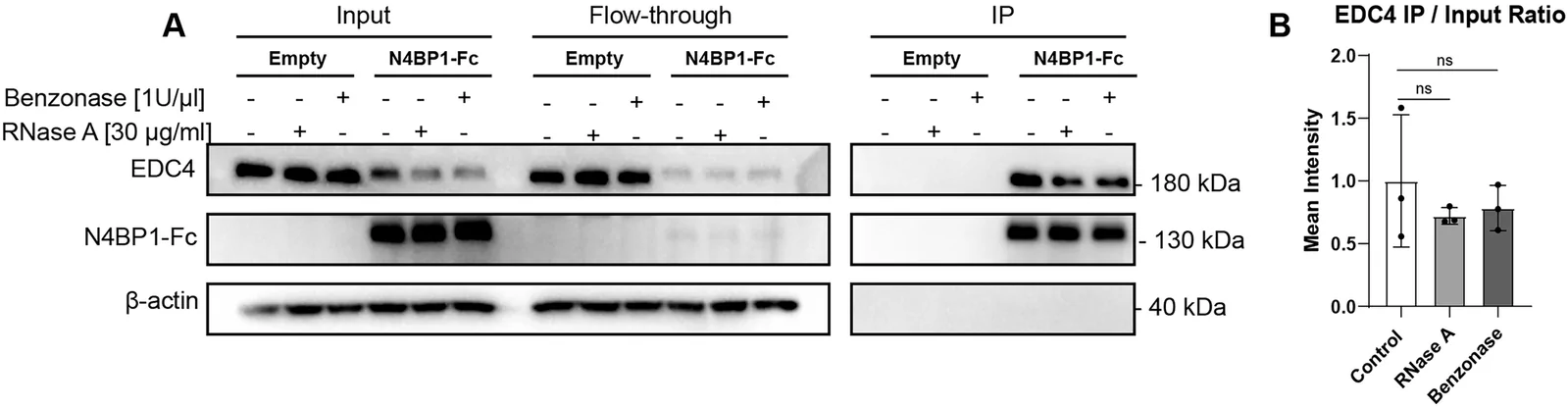
The interaction between N4BP1 and EDC4 is preserved upon RNase A or benzonase treatment. **A** Co-IP of N4BP1-Fc with EDC4 following RNase A or benzonase treatment for the indicated time points 2 h in HEK293T cells. **B** Densitometric quantification of Western blot signals from panel A, expressed as the EDC4 IP/Input ratio. Data represent mean ± SD from three independent experiments. Statistical analysis was performed using two-sided Brown-Forsythe and Welch’s ANOVA: Control vs RNase A *t*(2,06) = 0,91, *P* = 0,66, Control vs Benzonase *t*(2,46) = 0,67, *P* = 0,78.

The KH domains are known to bind the single-stranded (ss) loop regions of RNA hairpin structures^15,16^. To further investigate whether the N4BP1-EDC4 complex is stabilized by RNA, we performed immunoprecipitation experiments using HEK293T cell lysates pretreated with RNase A or benzonase for 2 h at 10 °C. As a control, 2 µg of purified RNA was incubated under the same experimental conditions as the cell lysate to verify RNase A and benzonase activity at 10 °C and RT. RNA degradation was confirmed under these conditions, demonstrating that the enzymes were active throughout the experiment (Fig. S8A). The N4BP1-EDC4 complex was then isolated using N4BP1-Fc as bait. Interestingly, we observed the presence of EDC4 in the immunoprecipitates derived from samples untreated and treated with RNase A and benzonase, indicating that RNA does not contribute to the interaction of both proteins (Fig. 5A, B). We also performed an additional experiment in which samples were treated with benzonase (Fig. S8B), followed by mass spectrometry analysis. We identified 11 proteins common to all N4BP1 pull-down datasets, including the benzonase-treated sample (Fig. S8C and Supplementary Data 1). Proteins whose association with N4BP1 remained unaffected by benzonase treatment included core components of the decapping complex, such as EDC4, DCP1A, EDC3, and DCP2. All these results indicate that the association of N4BP1 with the members of the decapping complex is largely independent of RNA-mediated interactions.

### N4BP1 regulates viral mRNAs independently of P-bodies and the decapping complex

N4BP1 is well known for its ability to degrade viral mRNAs, including those of HIV-1^4^. Recent studies have also shown that N4BP1 can suppress the replication of hepatitis B virus (HBV) by regulating viral pre-genomic RNAs (pgRNAs)^17^. To investigate the functional relevance of N4BP1’s KH domains and GXXG motifs in the suppression of HIV-1 gene expression, we co-transfected HEK293T cells with the psPAX2 vector encoding HIV-1 *gag* and *pol* genes and various N4BP1 expression constructs.

**Fig. 6:**
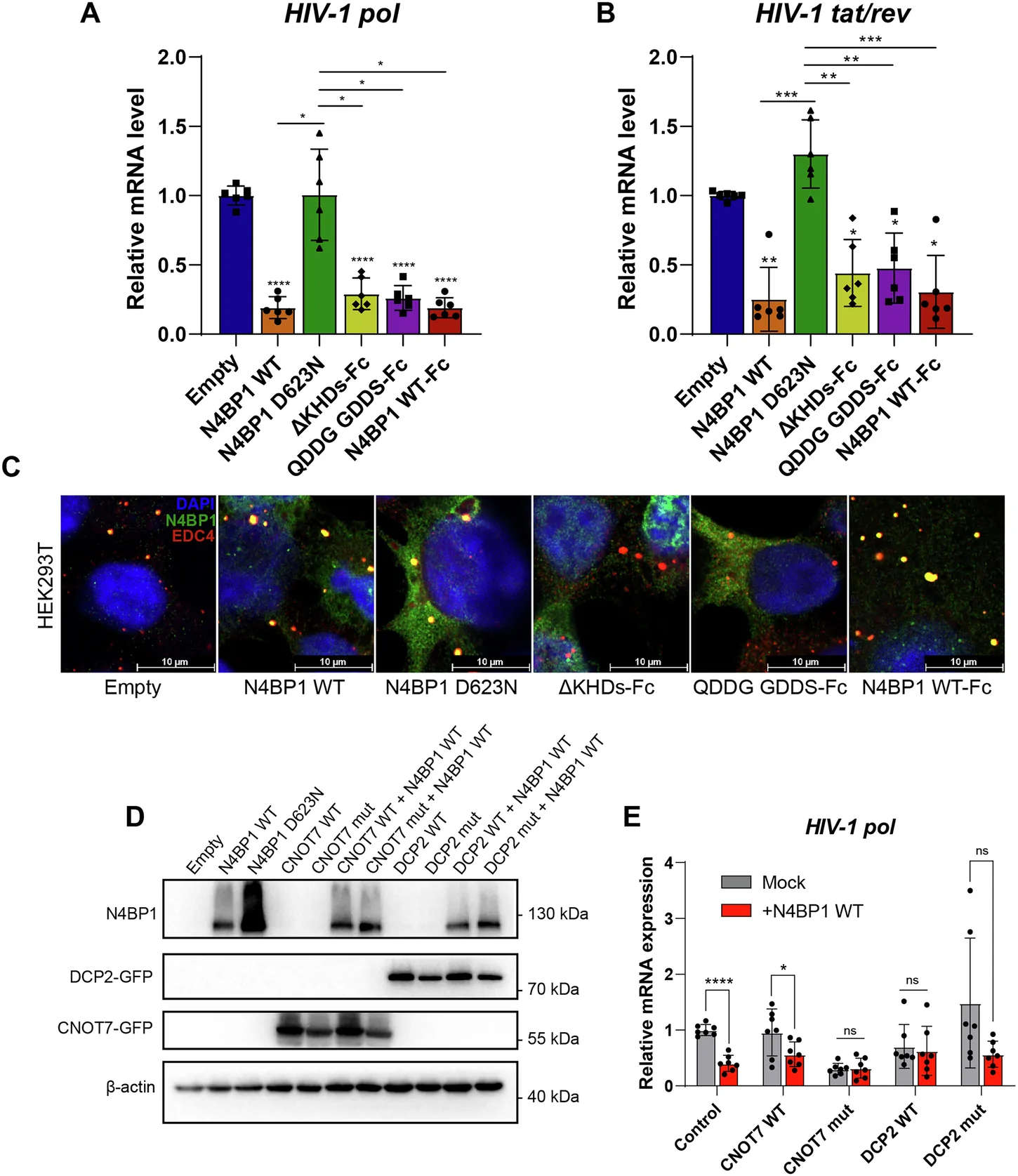
KH domains of N4BP1 are not required for degradation of HIV-1 transcripts. **A** Quantitative RT-PCR analysis of *HIV-1 pol* gene expression in HEK293T cells co-transfected with *psPAX2* and the indicated N4BP1 expression vectors at 48 h. Data presented are mean ± SD. Statistical analysis was performed using two-sided Brown–Forsythe and Welch’s ANOVA: F (5, 8.77) = 39,32; *P* < 0.0001. Significant differences were observed between control and active variants: empty vs N4BP1 WT *t*(9,84) = 18,84, *P* < 0,0001, empty vs ΔKH-Fc *t*(8,24) = 12,98, *P* < 0,0001, empty vs QDDG GDDS-Fc *t*(9,44) = 16,04, *P* < 0,0001, emp*t*y vs N4BP1 WT-Fc *t*(9,99) = 19,84, *P* < 0,0001. N4BP1 D623N vs N4BP1 WT *t*(5,57) = 5,88, *P* = 0,0109, N4BP1 D623N vs ΔKH-Fc *t*(6,19) = 5,02, *P* = 0,024, N4BP1 D623N vs QDDG GDDS-Fc *t*(5,72) = 5,34, *P* = 0,0177, N4BP1 D623N vs N4BP1 WT-Fc *t*(5,47) = 5,92, *P* = 0,0178 **B** Quantitative RT-PCR analysis of *HIV-1 tat/rev* gene expression under the same conditions as in (**B**). Data presented are mean ± SD. Two-sided Brown–Forsythe and Welch’s ANOVA: F(5, 24.95) = 21.00; *P* < 0.0001. Empty vs N4BP1 WT *t*(5,16) = 7,89, *P* = 0,0049, empty vs ΔKH-Fc *t*(5,14) = 5,62, *P* = 0,0221, empty vs QDDG GDDS-Fc *t*(5,13) = 5,03, *P* = 0,0352, empty vs N4BP1 WT-Fc *t*(5,12) = 6,43, *P* = 0,0124, N4BP1 D623N vs N4BP1 WT *t*(9,95) = 7,60, *P* = 0,0002, N4B*P*1 D623N vs ΔKH-Fc *t*(9,99) = 6,09, *P* = 0,0015, N4B*P*1 D623N vs QDDG GDDS-Fc *t*(9,99) = 5,70, *P* = 0,0026, N4BP1 D623N vs N4BP1 WT-Fc *t*(9,96) = 6,75, *P* = 0,0007. Data represents SD from independent biological replicates. Cells co-transfected with an empty pcDNA3.0 vector and *psPAX2* served as control. **C** Immunofluorescence staining of N4BP1 (green) and EDC4 (red) in HEK293T cells 48 h after co-transfection with *psPAX2* and N4BP1 expression constructs. The identity of each ectopically expressed N4BP1 variant is indicated below the respective image. **D** Western blot analysis confirming the expression of the indicated protein variants. **E** quantitative RT-PCR analysis of HIV-1 *pol* mRNA levels under the indicated conditions. Two-tailed paired t-test was used to calculate *P*-values for mock vs +N4BP1 WT comparisons. Data presented are mean ± SD. Control: *t*(6) = 9,95, *P* < 0,0001, CNOT WT: *t*(6) = 3,27, *P* = 0,0169, CNOT mu*t*: *t*(6) = 0,24, *P* = 0,815, DCP2 WT: *t*(6) = 0,45, *P* = 0,67, DCP2 mut: *t*(6) = 2,24, *P* = 0,066. Experiments were performed in 7 independent biological replicates.

Western Blot analysis and immunofluorescence staining (Fig. S9, S10) at 48 h post-transfection confirmed robust expression of wild-type (WT) N4BP1 as well as the D623N, ΔKHDs-Fc, QDDG GDDS-Fc mutants, along with Fc-tagged WT N4BP1. Overexpression of WT N4BP1 as well as WT N4BP1 tagged with Fc led to a significant reduction in HIV-1 *pol* and *tat/rev* mRNA levels at 48 h post-transfection (Fig. 6A, B), consistent with its previously described antiviral role. Similarly, both ΔKHDs and QDDG GDDS mutants of N4BP1 retained the suppressive activity, reducing viral mRNA levels to an extent similar to WT N4BP1. In contrast, the D623N point mutation within the ribonucleolytic NYN domain abolished the N4BP1 - mediated repression of HIV-1 transcripts, confirming prior findings on the nucleolytic activity of this domain of N4BP1^4^. These results indicate that the disruption of N4BP1’s KH domain integrity or loss of interaction with the key P-bodies component, EDC4, does not eliminate its antiviral activity. Furthermore, the alternation of binding motifs in KH domain leads to lack of colocalization with EDC4 and signal diffusion to the cytoplasm for either ΔKHDs or QDDG GDDS mutants of N4BP1 (Fig. 6C).

To check whether the mechanism of viral mRNA repression is deadenylation- or decapping-dependent, we performed experiments with expression of key proteins for those processes. To directly address this point, we co-expressed wild-type N4BP1 with either wild-type or catalytically inactive components of the CNOT/DCP2 - deadenylation/decapping complex and monitored HIV‑1 mRNA levels expressed from psPAX2 vector. In HEK293T cells transfected with psPAX2, we introduced either CNOT7-GFP (wild-type or catalytic mutant (D40A, E42A) or DCP2–GFP (wild-type or catalytic mutant (E148Q) with or without co-expression of wild-type N4BP1. Immunoblot analysis confirmed robust expression of all tested proteins, including WT and D623N N4BP1, DCP2-GFP, and CNOT7-GFP under all relevant conditions, with comparable loading as indicated by β‑actin (Fig. 6D).

We then quantified HIV‑1 *pol* mRNA levels expressed from psPAX2 vector by qRT-PCR. Ectopic expression of N4BP1 (red bars) led to a significant reduction in HIV‑1 *pol* mRNA levels in control samples as well as under co-expression conditions with wild-type CNOT7 (Fig. 6E).

Notably, the HIV-1 *pol* mRNA levels were similar in control cells expressing the mutant CNOT and in cells co-expressing N4BP1 with the mutant form of CNOT7. However, they were lower than in the corresponding control cells: empty or expressing only N4BP1 (Fig. 6E). In contrast, co-expression of N4BP1 with wild-type DCP2 or its catalytically inactive mutant did not affect the HIV-1 *pol* mRNA levels (Fig. 6E). These data suggest a decapping-independent mechanism of HIV-1 *pol* mRNA down-regulation.

### Regulation of eukaryotic transcripts by N4BP1

**Fig. 7:**
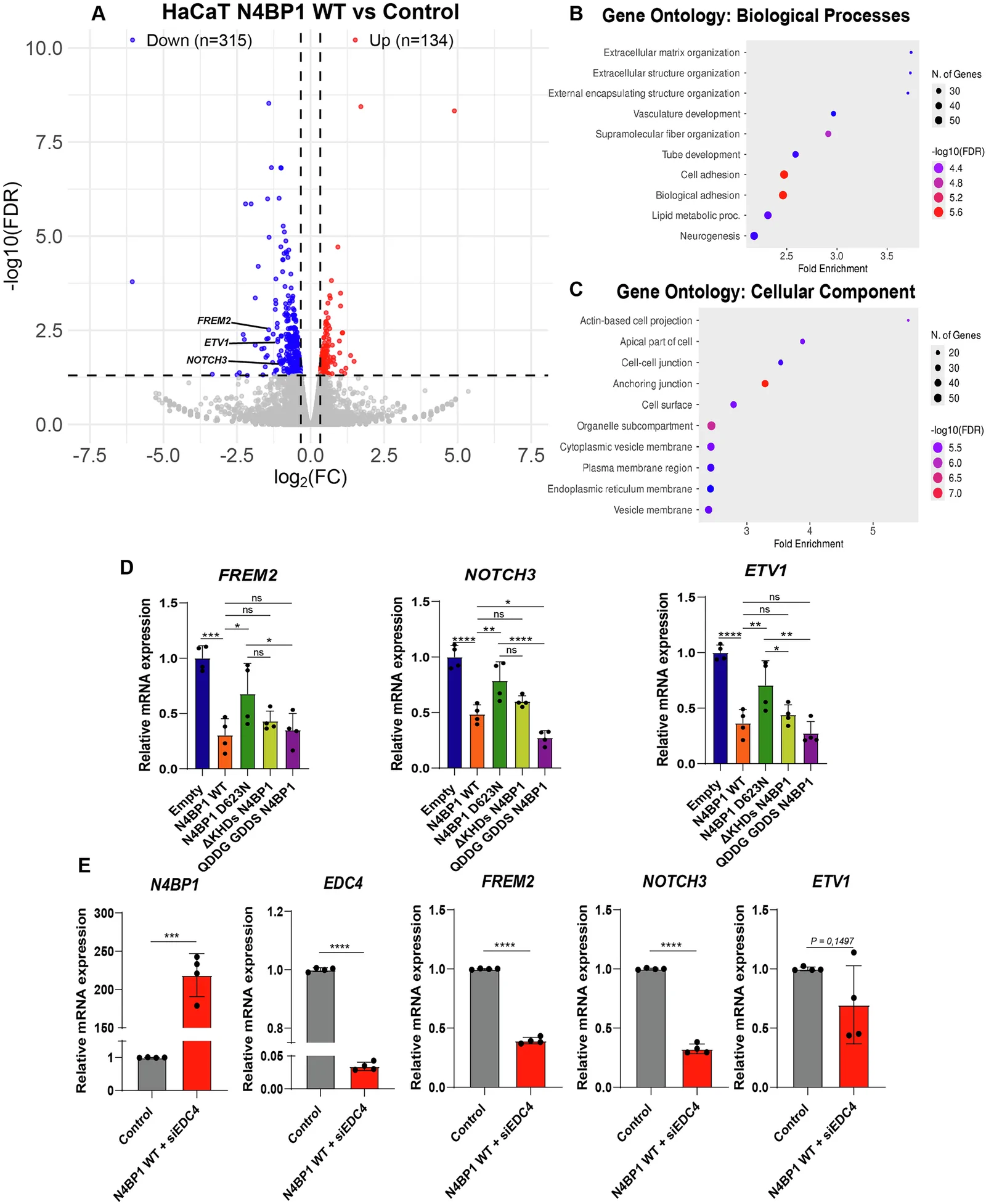
RNA-seq of HaCaT cells upon exogenous expression of wild-type (WT) N4BP1. **A** Volcano plot showing differentially expressed genes upon ectopic expression of WT N4BP1. Differential expression analysis was performed using edgeR. Statistical significance was assessed using a two‑tailed exact test with Benjamini–Hochberg correction for multiple testing. **B** Gene Ontology analysis of Biological Process terms enriched among significantly downregulated genes. **C** Gene Ontology analysis of Cellular Components terms enriched among significantly downregulated genes. Gene Ontology analysis presented at figures B and C was performed using ShinyGO v0.85. Statistical significance was assessed using a one‑tailed hypergeometric test (over‑representation analysis) with Benjamini–Hochberg correction for multiple testing. **D** Q-RT-PCR validation of selected transcripts used to confirm RNA-seq results. Data presented are mean ± SD. Statistical analysis was performed using two-sided one-way ANOVA: F(4, 8,61) = 11,73, *P* = 0,0015. *FREM2:* N4BP1 WT vs Empty *P* = 0,0001, N4BP1 WT vs N4BP1 D623N *P* = 0,023, N4BP1 WT vs ΔKHDs N4BP1 *P* = 0,68, N4BP1 WT vs QDDG GDDS N4BP1 *P* = 0,98, N4BP1 D623N vs ΔKHDs N4BP1 *P* = 0,16, N4BP1 D623N vs QDDG GDDS N4BP1 *P* = 0,049. *NOTCH3*: N4BP1 WT vs Empty *P* < 0,0001, N4BP1 WT vs N4BP1 D623N *P* = 0,0035, N4BP1 WT vs ΔKHDs N4BP1 *P* = 0,37, N4BP1 WT vs QDDG GDDS N4BP1 *P* = 0,038, N4BP1 D623N vs ΔKHDs N4BP1 *P* = 0,075, N4BP1 D623N vs QDDG GDDS N4BP1 *P* < 0,0001. *ETV1:* N4BP1 WT vs Empty *P* < 0,0001, N4BP1 WT vs N4BP1 D623N *P* = 0,0084, N4BP1 WT vs ΔKHDs N4BP1 *P* = 0,83, N4BP1 WT vs QDDG GDDS N4BP1 *P* = 0,74, N4BP1 D623N vs ΔKHDs N4BP1 *P* = 0,0412, N4BP1 D623N vs QDDG GDDS N4BP1 *P* = 0,0012. Experiment was performed in 4 independent biological replicates. **E** Q-RT-PCR of selected transcripts upon simultaneous ectopic expression of N4BP1 WT and silencing of EDC4 using specific siRNA. Data presented are mean ± SD. Two-tailed paired *t-*test was used to calculate *P*-values for Control vs N4BP1 WT + siEDC4 groups. *N4BP1 t*(3) = 15,5, *P* = 0,006, *EDC4 t*(3) = 511,7, *P* < 0,0001, *FREM2 t*(3) = 47,49, *P* < 0,0001, *NOTCH3 t*(3) = 38,37, *P* < 0,0001, *ETV1 t*(3) = 1,926, *P* = 0,1497. Experiment was performed in four independent biological replicates.

To determine whether N4BP1 also regulates eukaryotic endogenous transcripts, we performed transcriptome profiling in HaCaT keratinocytes upon ectopic expression of wild-type N4BP1, which allowed us to identify endogenous mRNAs whose abundance decreases in response to N4BP1 expression. RNA-seq analysis revealed several downregulated transcripts, including *FREM2, ETV1* and *NOTCH3*, which we prioritized as plausible N4BP1-sensitive targets based on their expression (Fig. 7A). Gene ontology (GO) analysis of biological processes revealed enrichment of differentially expressed genes (DEGs) involved in vascular development, highlighting 42 genes in this category (Fig. 7B). Other prominent processes affected included cell adhesion, cell differentiation, and intracellular transport. Complementary GO cellular component analysis linked these changes to genes associated with desmosomes, intercellular junctions, extracellular organelles, and vesicular transport structures (Fig. 7C). These data suggest a role for N4BP1 in modulating cellular connectivity and extracellular matrix dynamics, key for tissue organization and vascular function.

We then validated selected candidates by qRT-PCR and assessed their dependence on N4BP1 NYN catalytic activity and P-body localization. Specifically, we compared the effects of overexpressing wild-type N4BP1, N4BP1 with deleted KH domains (ΔKHDs N4BP1), N4BP1 with mutations in KH domains (QDDG GDDS N4BP1) and the RNase-inactive D623N mutant. In these experiments, wild-type N4BP1 significantly reduced *FREM2*, *ETV1*, and *NOTCH3* mRNA levels, whereas the D623N mutant failed to do so (Fig. 7D). Most importantly, transcript levels in cells ectopically expressing N4BP1 mutants lacking the KH domains or carrying mutations within these domains were significantly lower than in cells expressing the RNase-deficient NYN mutant of N4BP1 (D623N), and were comparable to the levels observed in cells ectopically expressing wild-type N4BP1. Additionally, we performed an experiment in which cells with ectopic expression of *N4BP1* had silenced expression of *EDC4* with specific siRNAs. As shown in Fig. 7E, the transcript levels of *FREM2*, *ETV1*, and *NOTCH3* were significantly reduced compared to cells treated with scrambled siRNA, indicating that N4BP1 degrades transcripts when EDC4 is silenced.

These results mirror our findings for transcript encoded by psPAX2 (HIV-1 *pol* and *tat/rev* mRNAs) and support the conclusion that the NYN domain is essential for N4BP1-mediated repression of selected both viral and endogenous transcripts represented by *FREM2*, *ETV1* and *NOTCH3* mRNAs, while the KH domains, which are required for the association of N4BP1 with EDC4 and localization in the P-bodies, are largely dispensable for the reduction of the analyzed mRNA.

## Discussion

Despite extensive research in recent years, many mechanistic aspects of post-transcriptional gene regulation remain poorly understood. RNA-binding proteins (RBPs), which play central roles in RNA processing, are typically characterized by specific, recurrent domains responsible for RNA recognition. These include: (i) the RNA Recognition Motif (RRM), approximately 90 amino acids in length, which interacts with 2-8 nt of single-stranded RNA, (ii) zinc finger domains, composed of about 30 amino acids that coordinate a Zn^2+^ ion or (iii) the K homology (KH) domain, approximately 70 amino acids in length, which recognizes 4-nt motifs in ssRNA or ssDNA^18^. RNA processing occurs within ribonucleoprotein (RNP) granules, which are found in various cell types and compartments. In the nucleus, these include Cajal bodies and paraspeckles, while in the cytoplasm, they encompass polysomes, processing (P-) bodies, and stress granules (SGs)^9,19^.

In this study, we identified the ribonuclease N4BP1 as a component of protein complexes residing within P-bodies. While N4BP1 was previously reported to localize predominantly to the nucleolus^20^, its involvement in diverse cellular processes—such as negative regulation of the NFκB pathway, inhibition of Itch autoubiquitylation, and suppression of HIV-1 gene expression^1,4,6,7,21^—suggests that it also functions in the cytoplasm. Using multiple experimental approaches, we confirmed the presence of N4BP1 in P-bodies. P-bodies are cytoplasmic RNP granules primarily composed of translationally repressed mRNAs and proteins involved in mRNA decay, microRNA silencing, nonsense-mediated mRNA decay, and splicing^22,23^. They share several components and stress-induced properties with stress granules. The mRNA decay process begins with deadenylation, first by the Pan2-Pan3 complex^24^ and subsequently by the CCR4-NOT deadenylase complex^25^. Following deadenylation, the DCP1-DCP2 decapping complex is recruited to hydrolyze the 5′- cap structure of mRNA. The activity of this complex is enhanced by enhancer of decapping (EDC) proteins, including EDC3 and EDC4. EDC3 is a conserved P-body marker essential for granule assembly, while EDC4 facilitates decapping by interacting with both DCP1 and DCP2^11,23,26,27^. Once decapped, mRNA is typically degraded by the 5′-to-3′ exonuclease XRN1.

We demonstrated that N4BP1 interacts with four key components of the mRNA decapping complex: EDC4, DCP1A, DCP2, and exonuclease XRN1. Using pull-down assays, we observed that siRNA-mediated knockdown of EDC4 led to a reduced association of DCP1A, DCP2, and XRN1 with the N4BP1 complex. This suggests that EDC4 is important for stabilizing the association between N4BP1 and other decapping factors.

Furthermore, IP-Western blot and immunofluorescence staining confirmed that N4BP1 does not localize to stress granules, as indicated by the lack of co-localization with the stress granule marker G3BP1. Mass spectrometry analysis of N4BP1 immunoprecipitates revealed the presence of proteins typically associated with stress granules, such as poly(A)-binding proteins (PABPs). It is important to note that these proteins also play a significant role in P-body formation, and their presence in P-bodies has been well established^13,14^. N4BP1 was reported to localize predominantly in the nucleolus and nuclear PML bodies^20^. Recent data show that N4BP1 may translocate to the cytoplasm, where it forms NEDD8-containing aggregates^28^. These aggregates, as well as P-bodies, have been shown to exhibit liquid-liquid phase separation (LLPS), suggesting a shared biophysical mechanism underlying their assembly and dynamics^28,29^.

In addition to its association with the DCP1-DCP2 decapping complex, EDC4 is known to associate with other protein complexes. For example, it interacts with the miRNA-induced silencing complex (miRISC) as well as the RNA-binding protein tristetraproline (TTP), thereby enhancing miRNA-mediated gene silencing^30–33^. More recently, EDC4 was shown to interact with the RNA-binding protein MARF1 (meiosis regulator and mRNA stability factor 1)^34–37^, which contains an NYN endonuclease domain adopting a PIN-like fold^36^ along with several RNA-binding regions^38^. EDC4 is responsible for repression of MARF1-mediated RNA decay by binding to the MARF1 C-terminus, sequestering it to P-bodies and preventing its association with target mRNAs. Interestingly, N4BP1 shares structural similarities with MARF, as it also contains a NYN domain. PIN/NYN domains are present in several other RNA-degrading enzymes, including monocyte chemotactic protein-induced protein (MCPIP1, also known as Regnase 1) and its related family members (MCPIP2, MCPIP3 and MCPIP4), all of which function as endonucleases^39–43^.

As a NYN-domain-containing endoribonuclease, N4BP1 is likely to possess an RNA-binding domain. The tandem KH domains appear to serve as a key motif responsible for direct RNA interaction^44,45^. Interestingly, the data indicate that the N4BP1 KH domains of N4BP1 are essential for its interaction with EDC4, and deletion of either KH-1 alone or both KH-1 and KH-2 completely abolishes this interaction. Moreover, loss of the KH domains alters the subcellular localization of N4BP1: the N-terminally truncated N4BP1 fails to co-localize with EDC4 in P-bodies and instead exhibits a diffuse cytoplasmic distribution.

Our structural analyses revealed that, although the KH domains of N4BP1 adopt the classical KH domain fold, they lack the conserved GXXG consensus motif typically found in RNA-binding KH domains. Specifically, the expected GXXG are mutated to QSRG in KH-1 and GAES in KH-2, suggesting that N4BP1’s tandem KH domains may be incapable of direct RNA binding. Notably, for N4BP1’s paralog KHNYN, it has been demonstrated that the KH domains do not bind RNA but instead serve as a potential protein interaction site^46^. Previous studies have shown that KH domains lacking the canonical GXXG loop lose the nucleic-acid-binding capacity and instead engage in extensive intramolecular protein–protein interactions^47,48^. Consistent with this, we found that mutating the cryptic GXXG motif in N4BP1, substituting QSRG with QDDG in KH-1 and GAES with GDDS in KH-2, disrupted the interaction between N4BP1 and EDC4. In contrast, wild-type N4BP1 readily interacts with EDC4. These findings suggest that the degenerate GXXG-like motif in N4BP1’s KH domains has evolved to support protein-protein interactions, particularly with other RNPs, rather than direct RNA recognition. It is likely that these interactions facilitate the recruitment of N4BP1 to RNA-containing complexes and mediate its association with target transcripts indirectly.

An AlphaFold-Multi^49^ predicted structural model for the N4BP1 tandem KH domains spanning 8–140 residues that are bound to EDC4 suggests that the KH-2 domain of N4BP1 docks onto the C-terminal helical fold (C-fold) of EDC4 (Fig. S11A–C). Three out of five predicted models (Fig. S12) converged to the presented structure model with pTM of 0.54 and ipTM of 0.25 and calculated Q-score of 0.28^50^. The EDC4 C-fold is electrostatically charged and occupies a spatial position analogous to the 3α motif in N4BP1. Notably, the GAES motif within KH-2 is oriented toward a negatively charged pocket on the EDC4 C-fold. Moreover, the N4BP1 residue Phe137 is predicted to engage in hydrophobic interactions with EDC4 Leu1390 (Fig. S11D). Our functional data—specifically, the disruption of the N4BP1-EDC4 interaction upon GAES-to-GDDS mutation—support the validity of the predicted structural model. Although our immunoprecipitation assays show KH-1 dependency on EDC4 interaction (Fig. 4D), the AlphaFold predicted model shows only the KH-1 QSRG motif pointing towards the EDC4 C-fold, without direct interaction. It is likely that the KH-1-EDC4 interface is modulated in the presence of other decapping complex components or other structural elements in EDC4 itself. The low Q-score and ipTM suggest that the predictions are not high confidence and require further investigation using an experimental structural approach.

It is now widely accepted that in the assembly of ribonucleoprotein (RNP) granules or condensates, such as P-bodies and stress granules, RNA plays a central role, acting as a key scaffold in the regulation of cellular networks. Owing to its molecular properties, RNA can simultaneously bind multiple proteins: while a 100-amino-acid polypeptide typically interacts with one or two proteins, an RNA molecule of 100 nucleotides can engage 5–20 proteins, making it an ideal platform for multivalent interactions^51–53^. Thus, we investigated whether RNA functions as a scaffold in the formation of the N4BP1-EDC4 association. In both cases, treatment of cell lysates with RNase A or benzonase prior to immunoprecipitation did not impair the association between core decapping proteins and N4BP1.

N4BP1-encoding gene expression is inducible by IFN-α and has been shown to selectively restrict HIV-1 infection of human T-cells and macrophages in a manner dependent on its NYN RNase domain^4^. In this study, we demonstrated that N4BP1 mutants lacking the ability to interact with decapping factors still reduce HIV-1 mRNA levels to a similar extent as wild-type N4BP1. This suggests that the antiviral activity of N4BP1 is independent of its interaction with canonical mRNA decapping components, such as EDC4, and does not require localization to P-bodies. Similarly, this pattern is observed for the analyzed eukaryotic transcripts. As demonstrated by our RNA-seq analysis, N4BP1 regulates numerous transcripts, like other NYN domain - containing proteins^40,41^ As with HIV-1 mRNA, this negative regulation is independent of interaction with EDC4 or the KH domains and depends only on the nucleolytic activity of the N4BP1 NYN domain. It is widely accepted that KH domains bind RNA and can function in RNA recognition^54^. However, our findings demonstrate that N4BP1 retains its nucleolytic function even when its KH domains are mutated or deleted, highlighting that recognition may occur through alternative mechanisms involving RNA-binding cofactors. One such candidate is, for example, TRIM25, which may contribute to selective targeting of viral transcripts^55^. An alternative scenario, requiring further investigation, is that EDC4 binding sequesters the KH domains of N4BP1, preventing RNA motif recognition. Upon dissociation of EDC4, the KH domains may become available for RNA binding, thereby enabling target RNA reduction. This scenario is consistent with the RNase A and benzonase experiment, where RNA degradation in protein lysates did not disrupt the association of N4BP1 with EDC4 and other core decapping proteins. Furthermore, this interaction may have a protective effect on transcripts regulated by N4BP1.

In summary, our findings demonstrate that N4BP1 is associated with the protein EDC4, which mediates a complex with the key components of the mRNA degradation machinery localized in P-bodies, including DCP1A, DCP2, and XRN1. The interaction between N4BP1 and EDC4 is dependent on the presence of both KH domains (KH-1 and KH-2) in N4BP1. Notably, the KH domains of N4BP1 lack the conserved amino acid residues required for canonical single-stranded nucleic acid binding, suggesting that their primary role may lie in mediating protein-protein interaction, but this interaction depends on the localization and complex composition. N4BP1 – mediated reduction of endogenous *FREM2, ETV1*, and *NOTCH3* transcripts as well as HIV mRNA is dependent on the NYN domain, and interaction with EDC4 via the KH domains does not affect this process. Further studies are essential to elucidate the role of RNA as a scaffold for the assembly of N4BP1 complex.

## Materials and methods

### Generation of N4BP1-Fc and N4BP1-GFP plasmids

cDNA isolated from HEK293T (human embryonic kidney) cells was used as a template to amplify the full-length N4BP1 coding sequence (NM_153029.4) using the following primers (forward 5′ TAGCTAGCGCAGCGGCAGCAAGCTGAG 3′ and reverse 5′ TTGGATCCTCCTCCATCCAACACCATGGCAGA 3′). The amplified product was ligated into Fc (Addgene number #141183) and GFP (Addgene number #141184) vectors, both based on a pcDNA3.1 backbone. Untagged N4BP1 expression constructs were generated using the pcDNA3.0 vector (Addgene number #2092). For all constructs, both vector and insert were digested with NheI and BamHI (NEB), gel-purified, and ligated overnight using T4 DNA ligase (NEB). Deletion and mutant constructs were generated using the N4BP1 WT-Fc pcDNA3.1 plasmid as a template. PCR amplification of N4BP1 fragments containing specific deletions or point mutations was performed using primers listed in Supplementary Table S1. PCR products were treated with DpnI (NEB) at 37 °C for 2 h to eliminate template DNA, phosphorylated using T4 polynucleotide kinase T4 (PNK) (NEB) at 37 °C for 30 min, gel-purified, and ligated overnight.

### Cell culture

HEK293T cells (ATCC) and HaCaT cells were cultured in Dulbecco’s Modified Eagle Medium (DMEM, Lonza) containing 4.5 g/l glucose and supplemented with 10% fetal bovine serum (FBS, Biowest). Cells were passaged 2–3 times per week depending on confluency, and the medium was refreshed every 2–3 days. HeLa cells were maintained in 25 cm^2^ flasks in DMEM containing 1 g/l glucose, supplemented with 10% FBS. HeLa cells were passaged twice weekly, with medium changed every 2–3 days. Biological replicates were collected after a minimum of three cell passages between biological experimental repeats.

### Immunoprecipitation

2 days before immunoprecipitation, HEK293T cells were seeded on 6-well plates (350,000 cells per well). 24 h later cells were transfected using respective plasmids (coding for GFP-tagged N4BP1 or Fc-tagged N4BP1) and the jetPRIME reagent (Polyplus). For identification of the interaction region, cells were co-transfected with N4BP1-Fc and EDC4 pcDNA3.0 at 2:1 plasmid ratio. For silencing of *EDC4*, 50 nM final concentration of adequate siRNA (Dharmacon) was used. After 24 h immunoprecipitation was performed according to the GFP-Trap Dynabeads (Chromotek) protocol. Cells were harvested using 200 µl of lysis buffer (10 mM Tris HCl pH 7.5, 150 mM NaCl, 0.5 mM EDTA, 0.5% Nonidet P40 Substitute, supplemented with 1 mM PMSF and protease inhibitor cocktail) per well and incubated for 30 min on ice. Lysates were transferred to Eppendorf tubes followed by centrifugation (17,000 g, 10 min, 4 °C) and diluted with 100 µl of dilution buffer (10 mM Tris HCl pH 7.5, 0.5 mM EDTA, supplemented with 1 mM PMSF and protease inhibitor cocktail). The input fraction was collected, and the lysates were transferred to a tube containing Dynabeads G (Invitrogen) or GFP-Trap magnetic beads (Chromotek), respectively, for Fc-tagged and GFP-tagged samples. Before adding lysates, immunoprecipitation beads were pre-washed with 300 µl of wash buffer (10 mM Tris HCl pH 7.5, 150 mM NaCl, 0.05% Nonidet P40 Substitute, 0.5 mM EDTA). Samples were rotated for 2 h at 4 °C, the flow-through fraction was collected, and the magnetic beads were washed three times with 300 µl of wash buffer. Complexes were eluted using SDS-based elution with 70 µl of SDS buffer (120 mM Tris-HCl, 20% glycerol, 4% SDS, 10% β-mercaptoethanol) for the GFP-tagged N4BP1 samples or by acidic elution with 70 µl of acidic elution buffer (200 mM glycine pH 2.5), neutralized with 7 µl of neutralization buffer (1 M Tris pH 10.4) for the Fc-tagged N4BP1 samples. Eluted complexes were extracted for subsequent mass spectrometry or preserved at −80 °C for Western blot analysis. Target sequences of the siRNAs are listed in Supplementary. Table 1.

### Protein extraction for MS

Four volumes of methanol were added to the samples, followed by the addition of 1 volume of chloroform. Samples were vortexed and three volumes of H_2_O were added. After 5 min of vortexing, samples were centrifuged (20,000 g, 1 min, RT) and the upper organic phase. Subsequently, three volumes of methanol were added. Following centrifugation (20,000 g, 5 min, RT), pellets were air-dried for 15 min and resuspended in phosphate-buffered saline (PBS). All volumes refer to the initial volume of each sample.

### Mass spectrometry analysis

High-resolution MS measurements of trypsin-treated samples after IP of N4BP1 complexes (N4BP1_Fc met_, N4BP1_Fc SP3_, N4BP1_GFP SP3_) were made using an Ultra-Performance Liquid Chromatography (UPLC) system combined with an LTQ-Orbitrap Velos mass spectrometer (Thermo Fisher Scientific). The obtained liquid chromatography-MS (LC-MS) data were analyzed using the MASCOT server (v. 2.5.1, Matrix Science), and results were used for peptide identification against the SwissProt database, restricted to *Homo sapiens* taxonomy. The quantitative analysis aimed at the determination of changes at the level of modified peptides was performed based on a comparison of the intensities of the extracted ion chromatograms between the label-free UPLC-MS measurements of immunoprecipitated samples. The sample background was removed by subtraction of MS data after IP against N4BP1 with the sample containing background from IP prepared using control HEK293T cells transfected with an empty vector. An additional immunoprecipitated samples, obtained after treatment of N4BP1 complexes with benzonase for 2 h in 4 °C (N4BP1_benzonase Fc SP3_), along with a control background immunoprecipitation, were subjected to SP3 resin purification. Subsequently both samples were suspended in 0.1% formic acid and peptide mixture was injected for measurement with Orbitrap Astral mass spectrometer coupled to Vanquish Neo UHPLC (Thermo Fisher Scientific). Peptides were separated using Aurora Ultimate XT 25 cm × 75 µm C18 column (IonOpticks) in acetonitrile. Gene Ontology analysis of detected by IP-MS complexes of N4BP1 was performed using g: Profiler^56^. For identification of GO association for N4BP1 complexes, we used the common set identified in all three datasets of IP-MS (N4BP1_Fc met_, N4BP1_Fc SP3_, N4BP1_GFP SP3_). This list contains 54 proteins.

### Immunoblotting

BCA measurements were performed on 96-well plates using bicinchoninic acid (Sigma) and copper sulfate solution (Sigma) (50:1 ratio). For SDS-PAGE, IP samples were mixed with 6x loading buffer supplemented with 20% β-mercaptoethanol. Proteins were resolved on 8% polyacrylamide gel, 20 min, 90 V, then 60 min, 120 V) and transferred to Immobilon-PVDF membranes (Merck Millipore) (1.5 h transfer, 100 V). After blocking the membranes for 1 h in 5% milk in Tris-buffered saline supplemented with 0.05% Tween20 (TBST), proteins were labeled by overnight incubation at 4 °C with primary antibodies against N4BP1 (rabbit, 1:3000, ab197079, Abcam, rabbit 1:2000, #A304-628A, Bethyl or rabbit mAb 1:1000, #E8H5E, Cell Signaling), EDC4 (rabbit, 1:1000, #2548S, Cell Signaling or rabbit, 1:1000, ab72408, Abcam), DCP1A (rabbit, 1:1000, A303-590A, Bethyl), DCP2 (rabbit, 1:1000, A302-597A, Bethyl), XRN1 (rabbit, 1:1000, A300-443A, Bethyl), G3BP1 (mouse, 1:1000, 66486-1-Ig, Proteintech), or β-actin (mouse, 1:3000, A1978, Sigma). Membranes were then washed three times using TBST and incubated with secondary antibodies (goat anti-mouse IgG HRP, SC-2005, Sigma or goat anti-rabbit IgG HRP, A0545, Sigma) for 1 h at RT. Signals were detected using an ImageLab imaging system (BioRad) with exposure stopped before signal oversaturation. Band intensities were measured using the Volume tools option in the ImageLab program. Graphs and statistical analysis were created using GraphPad Prism 8. Statistical significance is shown by asterisks in the graphs and is defined as follows: ∗*P* < 0.05, ∗∗*P* < 0.01, ∗∗∗*P* < 0.001, and ∗∗∗∗*P* < 0.0001. Exact *P*-values as well as names of the statistical tests used to generate *P*-values are provided in the figure legends.

### Immunofluorescent staining

Cells were seeded on 12-well plates at a density of 75,000 cells per well on glass coverslips and transfected 1 day after seeding with either empty pcDNA3.0 plasmid or N4BP1-GFP pcDNA3.0 variants. 24 h after transfection cells were washed twice with PBS for 5 min and fixated with 4% paraformaldehyde in PBS for 15 min. After fixation cells were washed three times with PBS and permeabilized with 5% BSA with 0.3% Triton X-100 solution in PBS for 1 h. After that cells were washed again three times and incubated overnight with primary antibodies in 1% BSA with 0.3% Triton X-100 in PBS at 4 °C. Primary antibodies used for endogenous protein staining were as follows: N4BP1 (rabbit, 1:200, #PA5-59323, Invitrogen), EDC4 (mouse, 1:200, sc-374211, Santa Cruz Biotechnology), DCP1A (rabbit, 1:200, A303-590A, Bethyl), DCP2 (rabbit, 1:50, A302-597A, Bethyl), XRN1 (rabbit, 1:50, A300-443A, Bethyl), G3BP1 (mouse, 1:50, 66486-1-Ig, Proteintech). Cells were then washed three times in PBS and incubated with the secondary antibodies (goat anti-mouse Alexa FluorTM 350 IgG (H + L), A11045, Invitrogen, goat anti-rabbit Alexa FluorTM 488 IgG (H + L), A11008, Invitrogen, goat anti-mouse Alexa FluorTM 546 IgG (H + L), A11003, Invitrogen or goat anti-rabbit Alexa FluorTM 546 IgG (H + L), A11035, Invitrogen) for 1 h in 1% BSA with 0.3% Triton X-100 in PBS at a 1:500 ratio. The cells were then washed three times with water and mounted on glass slides using ProLong Diamond Antifade Mountant with DAPI (Invitrogen). Microscope images were taken using Leica Stellaris 5 confocal microscope (HC PL APO CS2 63x/1.40 OIL objective, Power HyD S detectors). Threshold values for presented images were adjusted in Leica Application Suite (LAS X) software. Fluorescence pixel-by-pixel intensity measurement was done using ImageJ and data were calculated as mean intensity for 20 colocalizing granules (*N* = 3). MOC (Mander’s Overlap Coefficient) for granules was calculated using the BIOP JACoP plugin for FIJI ImageJ^57,58^. Details on MOC calculations are summarized in Supplementary Data 3.

### Cloning, expression and recombinant protein purification

Various cDNA constructs encoding the *Homo sapiens* (Hs) N4BP1 KH domain were subcloned into pQLinkH^59^ to express the KH domain with a N-terminal 7xHis tag, cleavable by TEV protease, in *E. coli* Rosetta 2 (DE3) cells (Novagen). Cultures were grown in Terrific Broth (TB) medium at 37 °C to an OD_600_ of ~1.0, then induced with 0.5 mM IPTG and incubated at 18 °C for 16 h before harvesting by centrifugation. The purification protocol consisted of the following steps, all performed at 4 °C: 1) Ni-affinity chromatography using a 5 ml HisTrap HP column (Cytiva), 2) cleavage of the 7×His-tag using TEV protease, 3) a second Ni-affinity chromatography to remove uncleaved protein and free tag, and 4) size-exclusion chromatography (SEC) using a Superdex 75 16/60 column (Cytiva).

Harvested *E. coli* pellets were resuspended in lysis buffer (1 X PBS pH 7.4, 500 mM NaCl, 5% (v/v) glycerol, 2.5 mM β-mercaptoethanol (β-Me)) supplemented with 1 μg/ml DNase I (Roche), 1 U/ml Benzonase (Merck) and one tablet of EDTA-free Complete Protease Inhibitor (Roche). Cells were disrupted using a microfluidizer (Microfluidics), and then lysate was separated by centrifugation at 45,000 g for 1 h. The supernatant was filter-sterilized through a 0.2 µm filter and applied to a 5 ml HisTrap HP column (Cytiva) pre-equilibrated with buffer 1 (25 mM HEPES pH 7.5, 200 mM NaCl, 10 mM imidazole, 2.5 mM β-Me). The column was washed with 20 column volumes (CV) of buffer 2 (25 mM HEPES pH 7.5, 500 mM NaCl, 25 mM imidazole, 2.5 mM β-Me), and bound protein was eluted with 5–10 CV of buffer 3 (25 mM HEPES pH 7.5, 200 mM NaCl, 250 mM imidazole, 2.5 mM β-Me). The eluate was dialyzed overnight against buffer 1 supplemented with 5% glycerol, 1 mM MgCl_2_ in the presence of 6×His-tagged-TEV protease (1 mg per 10 mg of target protein) to cleave the His-tag. Following cleavage, the sample was reapplied on a 5 ml HisTrap HP column to remove the cleaved tag and 6×His-TEV protease. The flow-through containing the tag-free protein was concentrated and subjected to the size-exclusion chromatography (SEC) using buffer 4 (25 mM HEPES pH 7.5, 150 mM NaCl, 1 mM DTT). Peak fractions were pooled, concentrated to ~9 mg/ml, flash-frozen in liquid nitrogen and stored at -80 °C after.

Selenomethionine (Se-Met) -labeled KH domain was expressed in *E. coli* B834(DE3) cells (Novagen) in LB media. The cultures were grown to an OD_600_ of ~0.6 and 125 mg/l Se-Met (Sigma) was supplemented in the *E. coli* culture for 30 min before inducing protein expression at 18 °C by adding 0.5 mM IPTG. *E. coli* cells were harvested 16 h post induction and Se-Met incorporated KH domain 8-194 protein was purified similarly as the native KH domain protein.

### Protein thermal-melting assay

Protein thermal-melting assays were done as described in Garg et al.^60^. In brief, 40 μg of different KH domain proteins were mixed with the Protein Thermal Shift Dye (ThermoScientific) in thermal melting buffer (25 mM HEPES pH 7.5, 150 mM NaCl, 1 mM DTT and 5% glycerol). Protein melting was measured over the temperature range from 20 °C to 94 °C using the Bio-RAD CFX96 Real-Time qPCR system.

### Protein-limited proteolysis

Limited proteolysis experiments were done using the trypsin, thermolysin or chymotrypsin proteases. Purified KHD proteins (5 μg) were incubated with the protease in a 1:100, 1:1000 and 1:10,000 w/w ratio and incubated at room temperature for up to 2 h. Samples were collected at 30 min, 1 h and 2 h time points, mixed with SDS-PAGE loading dye, and analyzed by SDS-PAGE.

### In-situ proteolysis and protein crystallization

Initial crystallization trials yielded tiny clusters of protein crystals for KHD^8-194^ but failed to yield single crystals after extensive optimization trials. Purified HsN4BP1 KH domain 8-194 protein was added with chymotrypsin protease in 1:10,000 w/w ratio immediately before setting up crystallization at 4 °C. KHD^8-194^ was crystallized using the sitting-drop vapor-diffusion method by mixing equal volumes of protein-chymotrypsin solution and reservoir buffer in a drop volume of 200 nl. Crystals of KHD^8-194^ were obtained by mixing 9 mg/ml protein (25 mM HEPES pH 7.5, 150 mM NaCl, 1 mM DTT) with 1.9 M sodium malonate (pH 5.0) as reservoir solution. No further optimization was performed for native KH domain crystals. Se-Met derivative KHD^8-194^ was similarly crystallized at 4 °C but using hanging-drop vapor-diffusion in a drop volume of 400 nl. All crystals were harvested and cryo-protected using reservoir solution supplemented with 20% ethylene glycol and flash-frozen in liquid nitrogen.

### Data collection, structure determination and refinement

X-ray diffraction data were collected at beamline BL14.1 operated by the Helmholtz-Zentrum Berlin (HZB) at the BESSY II electron storage ring, Berlin, Germany^61^ at wavelengths of 0.9184 Å and 0.9794 Å for native and derivative crystal, respectively. Data processing was performed using XDSAPP^62^, and the phase problem was solved by the single-wavelength anomalous dispersion (SAD) method using the Se-Met derivative dataset. After an initial round of automatic model building by Autobuild^63^, WinCoot^64^ was used for visualization and further model building, which was further refined using Phenix.Refine or CCP4.Refmac5^65^. All atom contacts and the geometry of the atomic models were evaluated using the Molprobity server^66,67^, and molecular drawings were generated using the PyMOL molecular graphics system (Version 2.4.1, Schrodinger, LLC). The APBS tool^68^ was used for calculating the electrostatic surface potentials. The data collection and refinement statistics for all structures are summarized in Table 2.

**Table 2:** Crystallographic data collection and refinement of crystal structure of KHD^8-194^ of N4BP1

Data collection statistics		
	Se-Met KHD^8-194^	Native KHD^8-194^
Space group	P6_2_ (171)	P6_2_ (171)
a, c [Å]	108.70, 27.64	108.69, 27.73
Resolution [Å] ^*a*^	47.07–1.95 (2.07–1.95)	47.06–1.88 (2.00–1.88)
No. of reflections	201,330	101,279
Unique reflections	26,371	15,568
R_meas_ [%] ^*b*^	8.1 (113.8)	6.3 (83.4)
<I/σ(I)>	15.54 (1.79)	19.78 (1.93)
CC_1/2_^*c*^	0.99 (0.79)	1.0 (0.74)
Completeness [%]	99.7 (98.8)	99.7 (99.1)
Multiplicity	7.63	6.51
**Refinement statistics for native KHD**^**8-194**^		
R_work_/R_free_^*d*^	0.181 / 0.226	
No. of protein atoms	1462	
No. of heteroatoms	0	
No. of solvents	106	
RMSD bond angles (°)	1.10	
RMSD bond lengths (Å)	0.005	
Ramachandran favored/outliers (%) ^*e*^	98 / 0.0	
Rotamer outliers (%)	0.0	

^a^Values in parentheses correspond to the highest resolution shell.

^b^R_meas_ = $$\frac{{\sum }_{{hkl}}\sqrt{\frac{n}{n-1}}{\sum }_{j=1}^{n}\left|{I}_{{hkl},j}-\left\langle {I}_{{hkl}}\right\rangle \right|}{{\sum }_{{hkl}}{\sum }_{j}{I}_{{hkl},j}}$$

^c^CC_1/2_ as defined by Karplus and Diederichs^74^

^d^R = Σ_hkl_ | Fo–Fc | )/Σ_hkl_ | Fo| for all reflections, where Fo and Fc are observed and calculated structure factors, respectively. R_free_ is calculated analogously for the test reflections, randomly selected and excluded from the refinement.

^e^As defined by Molprobity^66^.

### RNA isolation

For Q-RT-PCR assays HEK293T cells were seeded on 12-well plates at the density of 250,000 cells per well. 24 h later, the cells were transfected using the JetPRIME reagent (Polyplus) according to the producer’s protocol. For co-transfection experiments, 250 ng of psPAX2 plasmid (Addgene number #12260) and N4BP1 expression constructs (all cloned into the pcDNA3.0 backbone) were used per well. Additionally, for co-transfections with CNOT7 / DCP2 encoding vectors (Addgene numbers: 146890 and #147650) 250 ng of plasmids were added to the transfection mix. For silencing of *EDC4* expression specific *EDC4* siRNA (Dharmacon) (50 nM final concentration) was used. At 24–72 h post-transfection, the cells were lysed in 400 µl of Fenozol (A&A Biotechnology) per well and incubated on ice for 10 min. Chloroform was then added in 1:4 ratio. The samples were vortexed vigorously for 2 × 30 s followed by centrifugation at 12,000 g for 20 min at 4 °C. The upper phase was transferred to pre-chilled Eppendorf tubes, mixed with an equal volume of isopropanol and stored at −20 °C overnight. The next day, samples were centrifuged 14,000 g for 30 min at 4 °C. RNA pellets were washed twice with 70% ethanol prepared in DEPC-treated water, with centrifugation (14,000 g, 5 min, 4 °C) between washes. Pellets were air-dried and resuspended in 30 µl RNase-free water. To avoid plasmid DNA contamination, RNA samples were treated with DNase I (New England Biolabs) prior to reverse transcription. Sequences of used siRNAs are listed in Supplementary Table 1.

### Reverse transcription and quantitative PCR

For cDNA synthesis, 1000 ng of total RNA sample was mixed with 0.5 µl of 1 mg/ml oligo(dT) primers and incubated at 65 °C for 5 min. Reverse transcription was carried out by adding 5x M-MLV RT buffer (to a final 1x concentration), dNTPs (final concentration 0.5 mM each) and M-MLV reverse transcriptase (Promega, 100 units). Samples were incubated at 42 °C for 1 h, followed by enzyme inactivation at 70 °C for 15 min. cDNA was diluted 5-fold before use in qPCR. Amplification was performed using the 2× SYBR A RT PCR Mix (A&A Biotechnology) and 0.2 µM of each primer (Sigma-Aldrich) on a QuantStudio3 Real-Time PCR System (Thermo Fisher Scientific). 28S rRNA was used as a reference gene for normalization. Relative gene expression levels were calculated using a 2^-ΔΔCt^ method^69^. The primer sequences are listed in Supplementary. Table 1. Graphs and statistical analyses were generated using GraphPad Prism 8. Statistical significance is indicated in the graphs as follows: ∗*P* < 0.05, ∗∗*P* < 0.01, ∗∗∗*P* < 0.001, and ∗∗∗∗*P* < 0.0001.

### RNase A / Benzonase treatment

HEK293T cells were seeded at the density of 400,000 cells per well on 6-well plates. After 24 h cells were transfected with N4BP1-Fc pcDNA3.1 or an empty vector using JetPRIME reagent. Cells were lysed as described above (see Methods: Immunoprecipitation). After diluting the lysate with dilution buffer, RNase A (EURx) or Benzonase (Millipore) was added to a final concentration of 30 µg/ml or 1 U/µl respectively. Samples were incubated at 10 °C or RT for 2 h. Afterwards, the samples were processed according to the immunoprecipitation protocol using EDTA-free buffers supplemented with 2 mM Mg²⁺ ions instead of the standard EDTA-containing buffers. In the IP-MS experiment (Supplementary Fig. 8) Mg²⁺ ions were not supplemented and Benzonase activity therefore relied on endogenous divalent cations present in the lysate.

### Transcriptomics

RNA-seq was performed based on RNA isolated from HaCaT cell line. We used cells transduced with WT-N4BP1 or the empty PURO vector as control. To induce N4BP1 expression, doxycycline (Bioshop, 1 µg/ml) was added to the cells 24 h before harvesting. Additionally, cells were stimulated by 500 ng/ml PAM3CSK4 (Invivogen) for 2 h before RNA isolation. The RNA was isolated using Fenozol (A&A Biotechnology) according to the manufacturer's protocol. Subsequently, RNA was sent to the BGI Group where the DNBseq sequencing platform was used. RNAseq was performed using a library obtained from the polyA tails of transcripts (DNBSEQ). Paired sequencing was performed with read lengths of 150 bp (PE150), sequencing depth was 30 million reads - 6 GB of data per sample. RNAseq was performed on biological triplicates. The data was filtered for sequencing quality using SOAPnuke software^70^. Data after filtering the reads (fastq files) was analyzed on the Galaxy server using HISAT2^71^ that associated reads with specific genes, and next we used FeatureCounts software to quantify the number of reads per gene. Differential expression analysis comparing WT-N4BP1 or the control PURO systems was performed using edgeR^72^. The edgeR based on data provided by FeatureCounts, calculates the Fold Change (FC) value, which indicates the multiplicity of the change in the amount of a given transcript in the tested sample compared to the control sample. edgeR calculates the FDR (False Discovery Rate), which is the expected proportion of false discoveries among all results. The FDR level of 0,05 was adopted as the threshold for statistical significance in analyzed transcriptome data. The raw RNA-seq data were uploaded to NCBI Gene Expression Omnibus (GEO). The processed transcriptomic data after HISAT2 followed by FeatureCounts analysis were aggregated in the supplementary table (Supplementary Data 4), together with edgeR data showing differentially expressed genes that are dependent on N4BP1 overexpression in HaCaT cells. Genes that were classified as downregulated DEGs were subjected to Gene Ontology analysis using ShinyGo software (version 0.77; FDR cutoff 0,05)^73^.

## Supplementary information

Transparent Peer Review file

Supplementary Information

Description of Additional Supplementary files

Supplementary Data 1

Supplementary Data 2

Supplementary Data 3

Supplementary Data 4

## Data Availability

The atomic coordinates and structure factors for the crystal structure of HsN4BP1 KH domain^8-194^ have been deposited in the Protein Data Bank under accession code 6Q3V. Raw data obtained during IP-MS analysis of N4BP1 complexes are available in the MassIVE repository associated with the ProteomeXchange Consortium, under accession number: MassIVE MSV000094023, or PXD049196 [DOI:10.25345/C5F47H51H]. Biological replicates of immunocytochemistry images, Western blots, as well as raw membrane images and Q-RT-PCR calculations are accessible via Zenodo repository: https://doi.org/10.5281/zenodo.20107160. Access to RNAseq data has been publicly established on Gene Expression Omnibus server at NCBI. GEO Accession number: GSE282898. To review GEO accession please go to https://www.ncbi.nlm.nih.gov/geo/query/acc.cgi?acc=GSE282898. Raw, uncut Western blot membranes are presented in Figure. S13 in Supplementary Information file. Access to biological replicates of microscopy images, Western blot membranes, raw crystallography data and Q-RT-PCR calculations is available for reviewers using the following Zenodo repository link: https://zenodo.org/records/21900667?token=eyJhbGciOiJIUzUxMiJ9.eyJpZCI6IjBmYmI0NjI2LTJlOTAtNGMyOC04YzY4LWIwMTA1NjZkYzJjZiIsImRhdGEiOnt9LCJyYW5kb20iOiIwM2I4ZGQ3OTRiMTM2ZmRkYjU5MmUxYzMzNzMxNzhhZiJ9.rEqpROFPdqz9tTStng1fjooZMBdByLyDwnxAOsjTeUBqBaSfWlecxdy5WrcO0n1JLUyG82C_76LPV_0HBUb1PA.
