# Supplementary material for: N4BP1 uses tandem KH domains to associate with EDC4 and mRNA decapping factors in P-bodies
Source: Commun Biol. 2026 Sep 17;9:1215. doi: 10.1038/s42003-026-10938-x (PMC13586276; doi:10.1038/s42003-026-10938-x)
Supplement: Supplementary file 1 [file 42003_2026_10938_MOESM1_ESM.pdf]

nature portfolio

Peer Review File

N4BP1 uses tandem KH domains to associate with EDC4 and mRNA decapping factors in P-bodies

Corresponding Author: Professor Jolanta Jura

This file contains all reviewer reports in order by version, followed by all author rebuttals in order by version.

Version 0:

Reviewer comments:

Reviewer #1

(Remarks to the Author)
Reviewer Comments

My expertise is in RNA degradation, RNA biochemistry, and structural biology.

Summary:
Pilat and colleagues present an interesting study on N4BP1, showing that it interacts with decapping complex components, localises to P-bodies, and requires its KH1 domains for binding to EDC4. The structural analysis of the KH domains is carefully executed; however, several points remain unresolved. In particular, the direct versus RNA-mediated interactions of N4BP1 are not clarified, as IP–MS was not performed under nuclease treatment, and the sufficiency of the KH domains for binding is not established. The AlphaFold modeling is useful but requires additional validation (PAE plots, iPTM, or Q-scores) and does not fully align with the experimental findings regarding KH1. Further biochemical testing, such as EMSAs, would help establish whether the KH domains bind RNA or mediate protein–protein interactions.

Overall, the work is promising but requires further experiments to clearly define the molecular mechanism (direct vs indirect) of N4BP1 interactions.

Detailed comments:

In Figures 1 and 2, the authors determine and validate interaction partners of N4BP1, identifying several proteins from the decapping complex, including EDC4, DCP1/2, and XRN1. They further show that N4BP1 localises to P-bodies, consistent with its association with decapping complex components. In contrast, N4BP1 does not localise to stress granules. The authors then perform a structure–function analysis to identify which domains of N4BP1 are required for co-immunoprecipitation (co-IP) with EDC4. They demonstrate convincingly that the tandem KH1 domains at the N-terminus of N4BP1 are required, and that deletion of KH1 alone abolishes co-IP with EDC4. The structural analysis of the tandem KH domains is well executed, with sound refinement statistics.

Interestingly, the N4BP1 KH domains lack the canonical GXXG RNA-binding motifs. The authors test mutations of related motifs and assess their effect on EDC4 binding. Mutations in KH1 or KH2 impair co-IP with EDC4, while the double mutant destabilizes the protein, making interpretation difficult. For this reason, I would caution against drawing strong conclusions from the double-mutant experiment. In addition, for the localisation experiment in Figure 4F, the use of single mutants would have been more informative.

Major concern:
My main concern is that the direct interaction partners of N4BP1 remain unclear. The IP–MS experiments were not performed in the presence of RNase or Benzonase, meaning RNA-mediated interactions may be part of the observed network. Indeed, the authors' own data (Figures 5A/B) support this possibility. The reduction of co-IP with DCP1/2 and XRN1 upon EDC4 knockdown also suggests these interactions are indirect. It would also be important to know whether the authors considered performing IP–MS in the presence of Benzonase. Finally, the rationale for focusing on EDC4 would

benefit from further explanation. Was EDC4 chosen as a representative factor because it serves as a scaffold for recruiting DCP1/2 and XRN1?

The authors show that KH domains are required for interaction with EDC4, but they do not establish that these domains are sufficient. This experiment should be performed with KH1, KH2, and KH1+2 constructs.

The AlphaFold modeling is a valuable addition, but further details are required. Were the five models convergent? Please provide PAE plots and report the iPTM value. Since these are human proteins, it would also be useful to test them against the Walter lab's predictome resource, which provides Q-scores more sensitive than iPTM. https://predictomes.org/tools/spoc/

The AlphaFold prediction indicates that KH1 does not form direct contact with EDC4, which conflicts with the experimental data showing that deletion or mutation of KH1 disrupts binding. This discrepancy should be addressed. MOreoever, AlphaFold predicts the interface, which should then be validated from both sides: EDC4 mutants and N4BP1 mutants.

The surface properties of the KH domains suggest they may not bind RNA, but this remains speculative. A simple EMSA experiment would clarify whether the N4BP1 KH domains bind RNA or primarily function in protein–protein interactions.

Minor comments
In Figure 4A, it would be helpful to highlight the two loops that were mutated.

Reviewer #2

(Remarks to the Author)
The manuscript examines the role of N4BP1 in P-bodies and presents new observations. The data indicate that N4BP1 associates and colocalizes with decapping factors in cells while avoiding confusion with stress granules. The interaction weakens under RNase treatment, which suggests nucleic-acid mediated or nucleic-acid stabilized association. Functionally, suppression of HIV-1 transcripts requires the NYN catalytic activity and does not track with P-body localization. These findings are valuable, yet several gaps should be addressed before acceptance.

Current evidence makes a direct N4BP1–EDC4 interaction uncertain because the co-association is highly sensitive to RNase, which is more consistent with RNA or third-party protein bridging. When the KH region is compromised or the association with EDC4 is reduced, N4BP1 still lowers HIV-1 transcripts, whereas an RNase-dead NYN mutant loses activity. This pattern suggests that localization to P-bodies may not be required and that catalytic activity is central. If so, the key partners are more likely positioned along deadenylation and downstream decay pathways. These points call for a small, function-oriented set of additions.

Major:

-Clarify dependence on the 5′-to-3′ decay axis
Co-transfect catalytic-inactive CNOT7 or CNOT8 and assess whether N4BP1-mediated repression of HIV-1 or a standard reporter is attenuated as a readout for deadenylation dependence. Test a catalytic-inactive DCP2 or short-term XRN1 inhibition to see whether the N4BP1 effect is rescued, addressing decapping and 5'-3' exonucleolysis. Sensitivity at any node would support pathway-level coupling.

-Identify the operative partner rather than assuming EDC4
Perform a small co-IP screen under RNase conditions across CCR4-NOT core and catalytic subunits CNOT1, CNOT2 or CNOT3, CNOT6 or CNOT6L, CNOT7 or CNOT8, and CNOT9 to CNOT11. Include common P-body bridging factors such as EDC3, DDX6, Lsm14A and PatL1. RNase-resistant associations would be strong candidates for a protein bridge worth validating.

-Extend N4BP1 domain-function mapping beyond KH
Add targeted truncations or point mutants in the N-ter, the central low-complexity region, and the C-ter. Test interaction and localization with candidates emerging from the screen above to separate a localization platform role from interfaces that support functional partners.

-Provide a minimal endogenous validation set
Generate an N4BP1 knockout and perform RNA-seq to nominate two to three upregulated, biologically plausible targets. Measure half-life by actinomycin D time course or a simplified 4sU-pulse to confirm stabilization upon knockout. Conduct complementation with WT, ΔKH, and the RNase-dead D623N. Rescue by WT but not by D623N, with partial rescue by ΔKH, would extend the HIV-1 findings to endogenous transcripts. As a low-burden link to RNA substrates, RIP-qPCR for these candidates would suffice without high-throughput mapping.

Minor:

Adopt a cautious description of the N4BP1–EDC4 relationship and refer to it as RNA mediated or RNA stabilized until RNA bridging and third-party proteins are excluded. Introduce an alternative model in the Discussion that N4BP1 may act through CCR4-NOT or other P-body factors rather than relying on P-body localization per se. Present the quantification of P-body

colocalization more clearly in the main text and include input and integrity controls for RNase and knockdown conditions. If space permits, add a non-viral reporter such as a tethering assay or a representative 3' UTR to show that NYN dependence and KH dispensability generalize beyond the viral system.

In summary, this manuscript provides noteworthy information on localization and interactions and highlights the central role of N4BP1 catalytic activity in function. Completing the experiments above would elevate the current correlational evidence to a pathway-level argument and clarify N4BP1 true partners and biological scope.

Reviewer #3

(Remarks to the Author)
Pilat and co-authors found that N4BP1 interacts with EDC4 and other P-body proteins, The authors solved crystal structure of the KH domains of N4BP1, and found the interaction between the KH domain and EDC4 requires ssRNA. However, the KH domains were dispensable for N4BP1-mediated suppression of .HIV-1 RNAs.

Although the structural analysis of KH domains is the novel finding, the biological importance of the KH domain was not proposed in this study. The authors claim that N4BP1 predominantly localizes in P-body, the evidence that endogenous N4BP1 localizes in this granule is weak.
Further specific comments are following.

Major Comments
1. The authors show that endogenous N4BP1 interacts with EDC4 in cytoplasmic granule (Fig. 1G). However, the picture shows that N4BP1 is mostly localized in the nucleus with the granular pattern. The authors should precisely describe the results. If so, there are discrepancy between endogenous N4BP1 staining (Fig. 1G) and GFP-tagged one (Fig. 2A). The authors need to demonstrate if the expression pattern of N4BP1-GPF is not the overexpression artifact.
2. Also, the authors should validate the anti-N4BP1 antibody specifically precipitate this protein.
3. Recent studies demonstrated that association of N4BP1 with ubiquitin suppresses canonical NF-kB activation by suppressing NEMO activation or activation of non-canonical IKKs. Do the N4BP1-EDC4 interaction contribute to this activity? Furthermore, they should explore whether ubiquitination influences N4BP1’s interaction with EDC4 and P-bodies.
4. The structural study shown in Fig. 4 does not contribute to the study's conclusions and should be presented in a separate paper. The absence of the GXXG motif could be concluded without structural information.
5. The biological meaning of the interaction between N4BP1, P-bodies, decapping enzymes (including EDC4), and mRNA degradation is not clear.

Version 1:

Reviewer comments:

Reviewer #1

(Remarks to the Author)
Major Point 1
The experiments with RNase A treatment are difficult to interpret. Over time, N4BP1 pulls down less EDC4. This effect is stronger in the presence of RNase A. One explanation is that the interaction is indeed mediated by RNA.
However, the experiment with Benzonase does not show the same result. How do the authors know that Benzonase was working under these conditions?
The discrepancy between the RNase A and Benzonase experiments could be due to the following reasons. Benzonase needs Mg^{2+} as an essential cofactor for its activity. The optimal range is about 1 to 2 mM $MgCl_2$. The Lysis and IP buffer used here does not contain any Mg^{2+}. Instead, it contains 0.5 mM EDTA. EDTA chelates divalent cations and most likely blocks the activity of Benzonase. RNase A is a metal-independent enzyme, so it is not affected by this.
For these reasons, the experiments do not allow a clear conclusion. They need to be repeated.
The section title "RNA may not be necessary for stabilizing the interaction between N4BP1 and EDC4" is not a good choice. It is so vague that readers do not really know what to take away from it.

Major Point 2
These experiments clearly suggest that the KH domains 1 and 2 are required for the binding to EDC4. So this is done, great.

Points 3 and 4, AlphaFold
The authors mention that some models converge, but they do not mention how many. This information is only partly helpful. The authors should show all five models, or all five PAE plots. Then the reader can judge the data and does not need to run the prediction again.
Was AF2 or AF3 used here? The plots look more like AF3.
The authors also misunderstood what I asked for. The Walter lab offers several resources. First, they predicted a large number of protein-protein interactions. One can browse or screen these to see if a favorite prediction has already been reported. Second, they offer a validation server. This server calculates a Q-score based on the AF2 prediction. The Q-score is a better metric than the iPTM.

Another way to get a better measure than the iPTM is to use the LIVIA Prediction Analysis. It analyses AF3 predictions directly and provides the LIS score.
For the iPTM, no clear cutoff has been defined. As a rough rule, the IPTM should be above 0.5 for an interaction. The authors observe an iPTM of 0.25 for their best model. This suggests that there is no interaction, or that the predicted model is of very low confidence.
The authors should therefore use one of the two servers to cross-validate their results. In the current form, the AlphaFold analysis is not of sufficient quality to draw a conclusion. The LIS score analyses the five models individually and together. This allows a more robust judgement.
If the prediction confidence remains this low, it must be clearly stated in the text so readers are aware of it.

Point 5, RNA binding
The strongest point of the manuscript is the structure of the KH1-2 domains. The authors show that this domain tandem also mediates the binding to EDC4.
The authors determined the crystal structure of KH1-2. So they already established the production of the recombinant protein and it should be available.
I did not suggest an extensive systematic RNA binding analysis. I only suggested to test whether the protein can bind RNA at all. This would be very interesting, also with respect to Major Point 1, which still remains unclear. But I will no insist on this point and leaves this up to the editor to decide whether this is an important point or not.

Reviewer #2

(Remarks to the Author)
I think the revised manuscript is substantially improved. The revision has addressed most of my previous concerns. In particular, the new domain-mapping data and the benzonase-treated IP-MS experiment help clarify the relationship between N4BP1, EDC4, and other decapping factors. The added RNA-seq and qRT-PCR analyses also make the functional part of the study more complete.

The revised data now support a more nuanced model than the original manuscript. The tandem KH domains appear to mediate the association of N4BP1 with EDC4 and P-body-associated decapping factors, whereas the reduction of HIV-1 transcripts and selected endogenous transcripts is mainly linked to the NYN domain. This distinction is important and should remain clear throughout the manuscript.

1. I suggest softening the wording that describes N4BP1 as a component of the mRNA decapping complex. The data show association and colocalization with decapping factors, but they do not establish that N4BP1 is a core or functional component of the decapping machinery. For example, the section title “N4BP1 RNase is a component of mRNA-decapping complex” could be revised to something more neutral, such as “N4BP1 associates with mRNA-decapping factors.”

2. The endogenous transcript analysis should also be described with some caution. Because the RNA-seq experiment is based on ectopic N4BP1 expression, and because transcript half-life or direct RNA binding was not tested, FREM2, ETV1, and NOTCH3 are better described as N4BP1-sensitive transcripts rather than direct endogenous targets. Along similar lines, “transcript reduction” or “N4BP1-mediated repression” may be more appropriate than “degradation” in places where RNA stability was not directly measured.

3. The RNase A and benzonase results should be interpreted carefully. The RNase A experiment suggests that RNA may contribute to the N4BP1–EDC4 association, whereas the benzonase experiment indicates that at least part of the N4BP1-associated decapping-factor complex is resistant to nuclease treatment. These results are useful, but they do not by themselves establish a direct binary interaction between N4BP1 and EDC4. The text should therefore continue to distinguish between direct binding and association within a larger protein or RNP complex.

I would support publication after these textual clarifications.

Version 2:

Reviewer comments:

Reviewer #1

(Remarks to the Author)
The Authors have repeated the experiment with Benzonase. Now the picture is more clear.
What is unclear to me: Was the IP mass spec experiment with Benzonase repeated in presence of Mg2+ ions or is the experiment, which was moved to the supplment (Suppl. Figure 8) the old one? The Materials and Methods section would, to me, suggest that IP-MS is the same, but the experiment for Figure 5 is new.

If so, the caveat should be clearly stated.

The discussion section AF is good now; it clearly shows the limitations of the prediction for the readers.

So overall I am ok.

Open Access This Peer Review File is licensed under a Creative Commons Attribution 4.0 International License, which permits use, sharing, adaptation, distribution and reproduction in any medium or format, as long as you give appropriate credit to the original author(s) and the source, provide a link to the Creative Commons license, and indicate if changes were made.
In cases where reviewers are anonymous, credit should be given to 'Anonymous Referee' and the source.
The images or other third party material in this Peer Review File are included in the article's Creative Commons license, unless indicated otherwise in a credit line to the material. If material is not included in the article's Creative Commons license and your intended use is not permitted by statutory regulation or exceeds the permitted use, you will need to obtain permission directly from the copyright holder.
To view a copy of this license, visit https://creativecommons.org/licenses/by/4.0/

Our responses to the reviewers are presented below in black. Changes made in the manuscript are highlighted in red. Likewise, revisions to the supplementary data submitted as separate files are also indicated in red.

ANSWERS TO THE REVIEWER #1:

We would like to sincerely thank the Reviewer for all the questions and comments. We have conducted additional experiments, which we hope will help clarify any doubts and improve the scientific message.

Major concern:

1. My main concern is that the direct interaction partners of N4BP1 remain unclear. The IP–MS experiments were not performed in the presence of RNase or Benzonase, meaning RNA-mediated interactions may be part of the observed network. Indeed, the authors' own data (Figures 5A/B) support this possibility. The reduction of co-IP with DCP1/2 and XRN1 upon EDC4 knockdown also suggests these interactions are indirect. It would also be important to know whether the authors considered performing IP–MS in the presence of Benzonase. Finally, the rationale for focusing on EDC4 would benefit from further explanation. Was EDC4 chosen as a representative factor because it serves as a scaffold for recruiting DCP1/2 and XRN1?

There were two reasons why EDC4 was selected for further analysis: the number of reads in the MS analysis and the fact that it serves as a scaffold for recruiting DCP1/2 and XRN1. We performed additional experiments to further evaluate whether the association between N4BP1 and the decapping complex is maintained under RNase-treated conditions, thereby minimizing potential RNA-mediated interactions. In line with the Reviewer's suggestion, we included benzonase during N4BP1 immunoprecipitation (**Fig, 1A, B**) and performed an additional mass spectrometry analysis. The resulting data revealed a stable core N4BP1-associated complex that was resistant to benzonase treatment

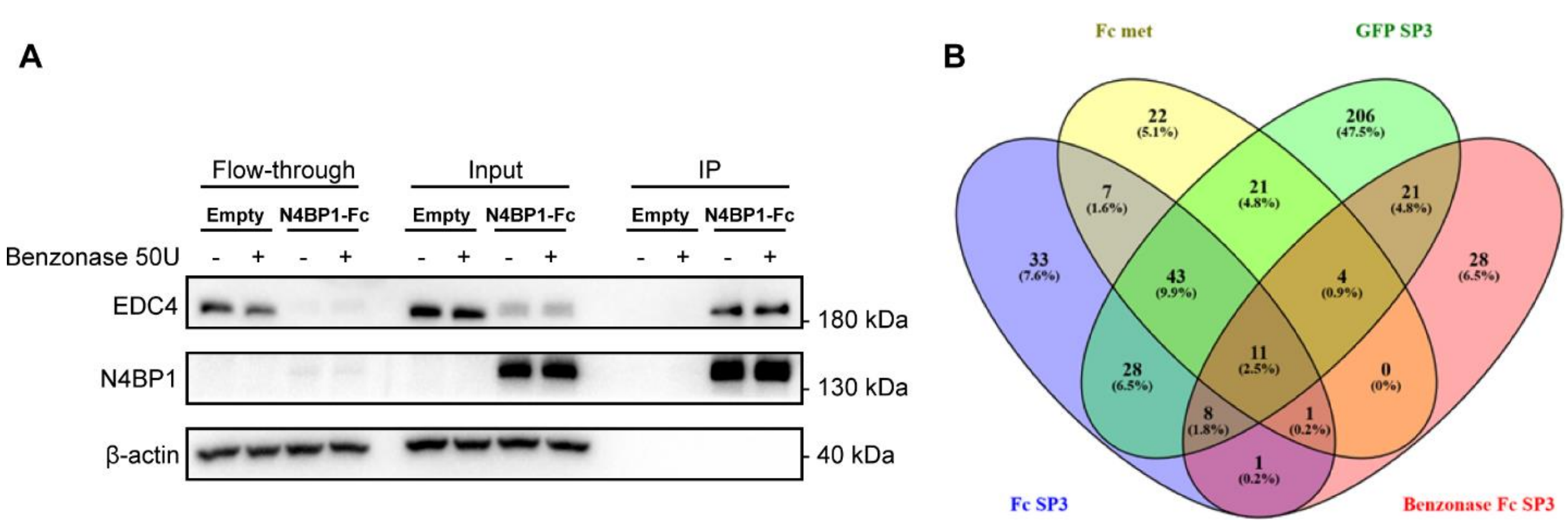

Fig. 1 (Fig. 5C in the manuscript and Fig. S8 in Supplementary information). Benzonase treatment does not abolish the interaction between N4BP1 and EDC4.

A. Western blot analysis of samples treated with benzonase prior to immunoprecipitation.

B. Venn diagram illustrating number of proteins identified by mass spectrometry under all tested conditions.

On the basis of obtained results we changed the title of the section: "**RNA is required for the interaction between N4BP1 and EDC4" into "RNA may not be necessary for stabilizing the interaction between N4BP1 and EDC4**". We also modified the text and added more information to the main text of the manuscript based on obtained results (page 9 in the manuscript, lines 289-302):

To further investigate whether the N4BP1-EDC4 complex is stabilized by RNA molecules, we performed immunoprecipitation experiments using HEK293T cell lysates pretreated with RNase A for 0.5 and 2 h. The N4BP1-EDC4 complex was then isolated using N4BP1-Fc as bait. Interestingly, we observed a time-dependent decrease in EDC4 levels in the immunoprecipitates in both untreated and RNase A - treated samples relative to the control (0 h). However, this effect was more pronounced in the RNase A - treated samples. At the 2-hour time point, the difference between samples with and without RNase A was statistically significant (Fig. 5A, B). These results suggest that RNA may contribute to the interaction between N4BP1 and EDC4.

*To further examine this observation, we performed an additional experiment in which samples were treated with benzonase, followed by mass spectrometry analysis. We identified 11 proteins common to all N4BP1 pull-down datasets (Fig. 1C), including the benzonase-treated sample, indicating that their association with N4BP1 is largely independent of RNA-mediated interactions (**Fig. S8, Suppl. Table 2**). Proteins whose interaction with N4BP1 remained unaffected by benzonase treatment included core components of the decapping complex, such as EDC4, DCP1A, EDC3, and DCP2.*

Besides changes in the text we made the following modifications: **Fig. 5 in the manuscript** has been expanded and contains Western blot results from experiment with benzonase; **Supplementary Table 2** has been updated with IP-MS results after benzonase treatment; **Supplementary Fig. S8** has been added to show Venn diagram illustrating number of proteins identified by mass spectrometry under all tested conditions including benzonase treated sample.

2. The authors show that KH domains are required for interaction with EDC4, but they do not establish that these domains are sufficient. This experiment should be performed with KH1, KH2, and KH1+2 constructs.

The second major point raised by Reviewer concerned whether the tandem KH domain of N4BP1 is sufficient to mediate recognition of EDC4 and association with the decapping complex. To address this, we generated expression constructs encoding the tandem KH domain, as well as the individual KH1 and KH2 domains, and established a pull-down system to test their interaction profiles. Fc-tag-based pull-down experiments demonstrated that, despite the multidomain architecture of N4BP1, the N-terminal region containing the tandem KH domains is sufficient to independently mediate association with EDC4 and the

core proteins of decapping complex (**Fig. 2**). On the other hand, EDC4 failed to co-immunoprecipitate with N4BP1's single KH-1 or KH-2 domains (**Fig. 3A, B**). These highly valuable results extend our previous observation based on mutagenesis of the GXXG binding motifs in KH domains that are mandatory for association with EDC4 (**Fig. 4D, E in the manuscript**).

In the subsection: "**The KH domain is mandatory for N4BP1 interaction with EDC4" of the Results of the manuscript the following text was added** (page 7 in the manuscript, lines 220-227)**:**

*To further determine whether the tandem KH domains are sufficient for EDC4 binding, we performed pull-down analyses using constructs expressing either the tandem KH domains or the individual KH1 and KH2 domains. These experiments demonstrated that the tandem KH domains are sufficient for association with EDC4 as well as with components of the decapping complex (**Fig. 3F, G, Suppl. Fig. S3**). Moreover, a substantial fraction of endogenous EDC4 was recovered from cells ectopically expressing either full-length N4BP1 or the tandem KH domains, as evidenced by reduced levels of EDC4 in the flow-through fraction following pull-down (**Fig. 3G, Suppl. Fig. S3**). This indicates efficient capture of endogenous EDC4 by the overexpressed tandem KH domains of N4BP1.*

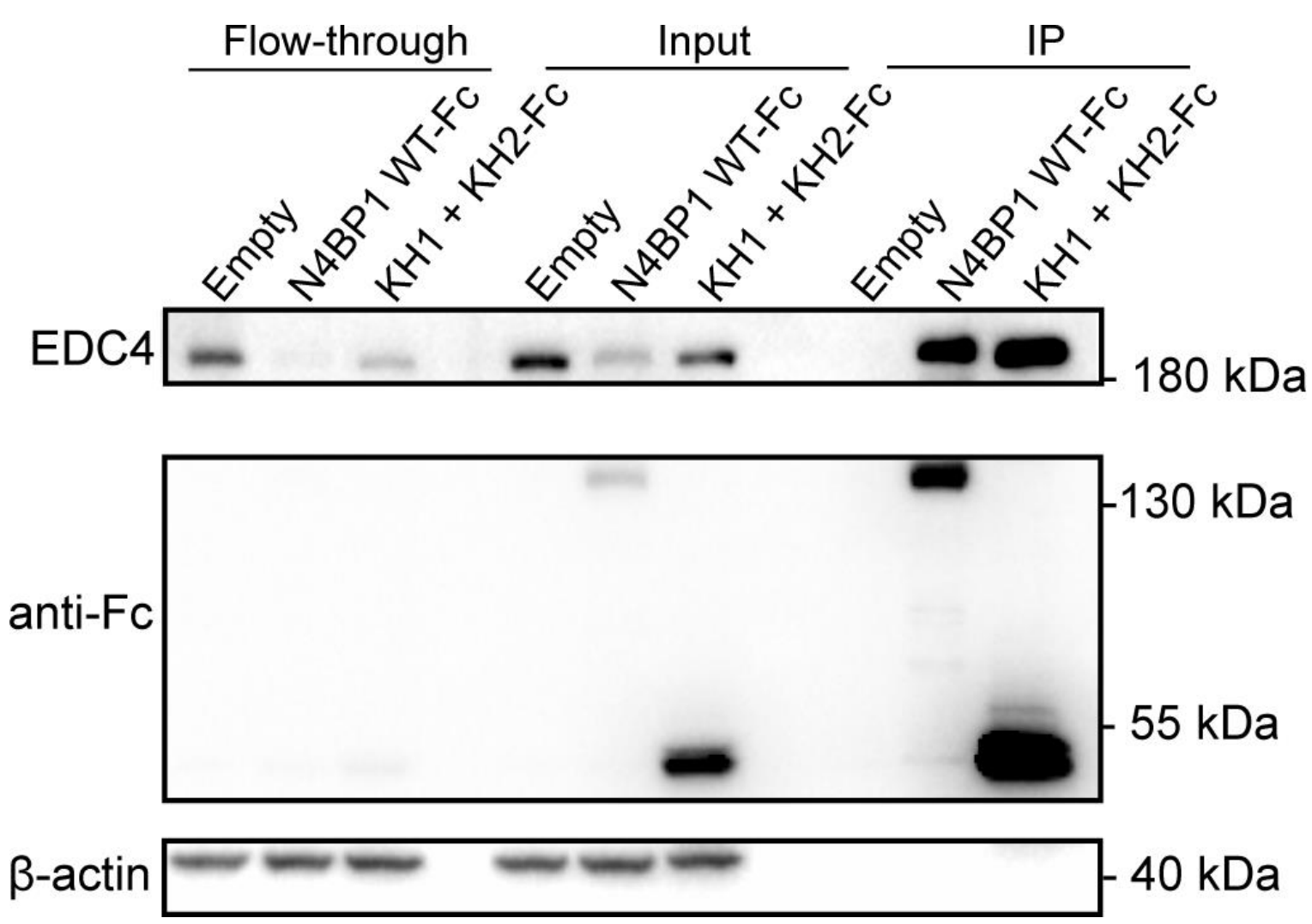

Figure 2 (Fig. 3G of the manuscript). Immunoprecipitation assay of full length N4BP1 and its tandem KH domain respectively, followed by Western blot analysis. Presented results are representative of 3 biological repetitions.

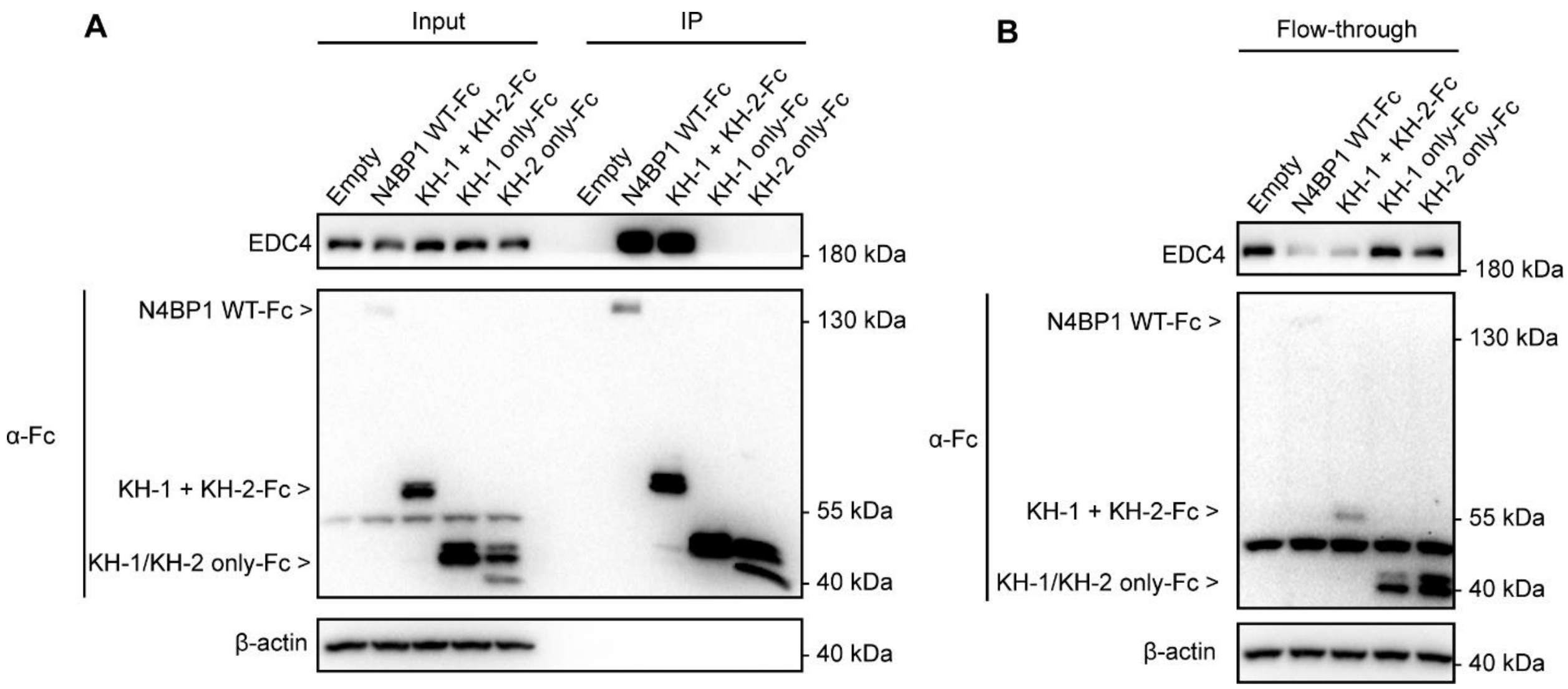

Fig. 3 (Fig. S3 in the Supplementary information): Analysis of the impact of the KH domains of N4BP1 on its association with the decapping complex, represented by EDC4. A. Pull-down assay of full length N4BP1 and its N-terminal fragments (tandem KH domains, KH1 and KH2), followed by Western-blot analysis. **B.** Western blot analysis of flow-through fractions from the pull-down assay of N4BP1s fragments. Presented results are representative of 3 biological replicates.

Figure 3 in the manuscript has been updated to clearly demonstrate that tandem KH domains are sufficient for interaction with EDC4 (**Fig. 3F, G in the manuscript**).

Supplementary figure has been added as follows.

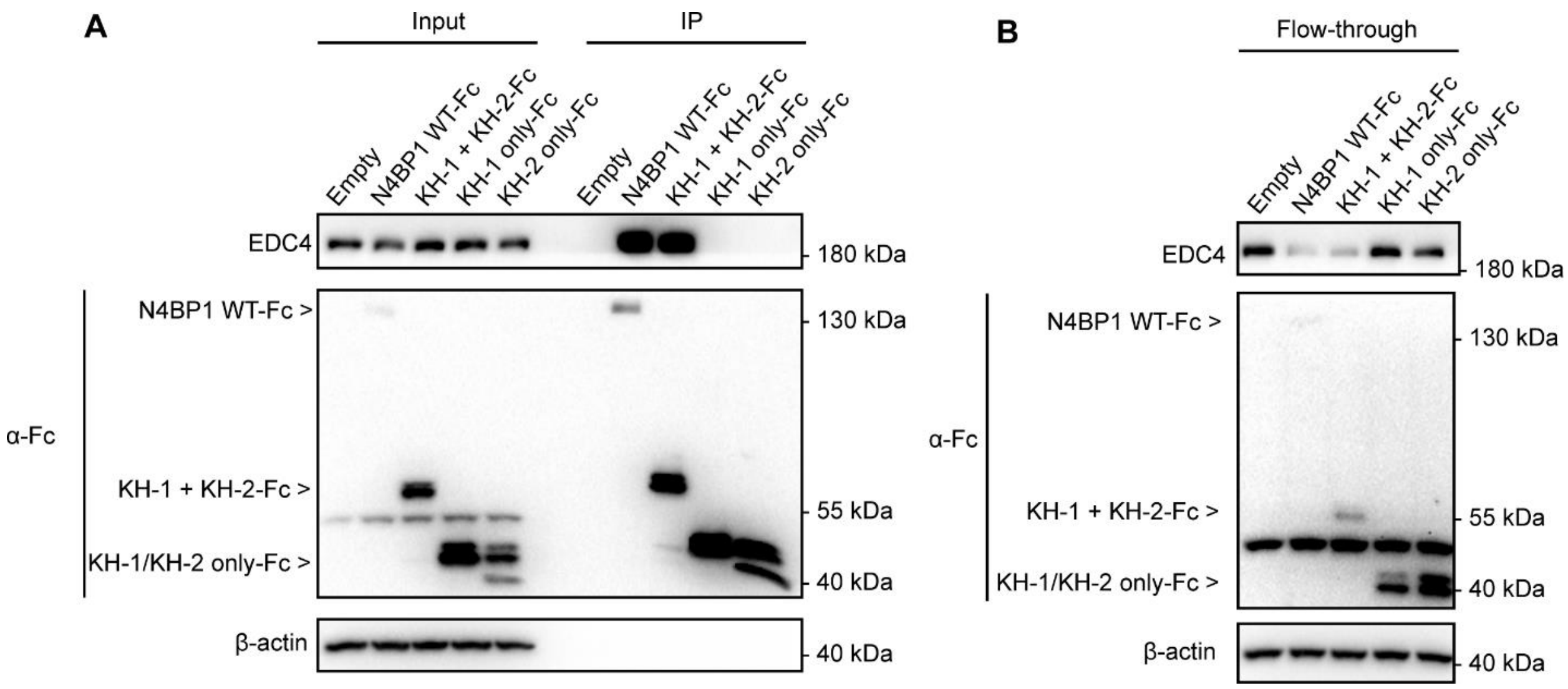

Fig. 4: (Suppl.Fig.S3 in the manuscript) Analysis of the impact of the KH domains of N4BP1 on its association with the decapping complex, represented by EDC4. A. Pull-down assay of full length N4BP1 and its N-terminal fragments (tandem KH domains, KH1 and KH2), followed by western-blot analysis. **B.** Western blot analysis of flow-through fractions from the pull-down assay of N4BP1s fragments. Presented results are representative of 3 biological replicates.

3.The AlphaFold modeling is a valuable addition, but further details are required. Were the five models convergent? Please provide PAE plots and report the iPTM value. Since these are human proteins, it would also be useful to test them against the Walter lab's predictome resource, which provides Q-scores more sensitive than iPTM. https://predictomes.org/tools/spoc/

In our AlphaFold prediction, several predicted models converged to the presented structure and showed a similar interaction pattern between KH domains and EDC4 C-terminal domain. The PAE plot image is added as figure **S11B**. The observed pTM and ipTM values for the prediction are also added to the section describing the Alphafold structure. In Walter lab's predictome, we did not find EDC4 as a strong interaction partner of N4BP1 and vice versa, so we have not mentioned about it in the manuscript.

4. The AlphaFold prediction indicates that KH1 does not form direct contact with EDC4, which conflicts with the experimental data showing that deletion or mutation of KH1 disrupts binding. This discrepancy should be addressed. Moreover, AlphaFold predicts the interface, which should then be validated from both sides: EDC4 mutants and N4BP1 mutants.

As we also described in point 2 of reviewer remarks, both the tandem KH domains are important for EDC4 interaction (Figure 2). However, several AlphaFold predicted structure models converge to the presented model (Fig S11), which exhibits a direct protein-protein interaction for KH2 and EDC4, but only predicts KH1 QSRG motif pointing towards the EDC4. It is likely that the interface between KH1 and EDC4 could be influenced by other decapping complex components or other elements in EDC4 itself. We plan to pursue a cryoEM structure of the whole complex to characterize this interface in our future study, and for the scope of this manuscript we intended to use AlphaFold analysis to support observations from our cell-based assays. We have now clearly mentioned about the discrepancy about KH1-EDC4 interface in the manuscript with a possible explanation.

5. The surface properties of the KH domains suggest they may not bind RNA, but this remains speculative. A simple EMSA experiment would clarify whether the N4BP1 KH domains bind RNA or primarily function in protein–protein interactions.

The detailed binding properties of the isolated KH domains, both with respect to nucleic acid recognition and interaction with EDC4, are currently being investigated in a separate project using complementary biophysical and structural biology approaches, including SAXS, cryo-EM, and crystallography. We have successfully produced recombinant KH domains and EDC4 fragments in E. coli, and these proteins are plan to being used in ongoing biophysical studies aimed at further dissecting the N4BP1 interaction mechanism.

We did not perform EMSA-based analysis of nucleic acid binding for the present manuscript, as such experiments would extend beyond of the scope of the current study and are being pursued as part of a dedicated follow-up project, including determinations of dissociation constants for the relevant complex. The nucleic acid-binding properties of the KH domains, as well as the relationship between RNA binding and protein-protein interactions, represent important questions that we plan to address in future biophysical studies. Notably, the tandem KH domain of N4BP1 shows structural similarity to KH domains from NOVA1/2 (**Suppl. Fig 5**), for which RNA-bound structures have been solved, including complexes with stem-loop RNA elements. This structural resemblance is consistent with a potential RNA-binding role for the N4BP1 KH domains, although this hypothesis remains to be experimentally validated.

Minor comments

In Figure 4A, it would be helpful to highlight the two loops that were mutated.

Figure 4A has been changed according to Reviewer's suggestion. Mutated loops are shown now in red.

Because we performed additional experiments to the manuscript the Abstract has been updated accordingly to reflect the newly generated results. The new part of the abstract is written in Italics (page 2 in the manuscript, lines 60-71):

Processing bodies (P-bodies) are cytoplasmic, non-membrane-bound structures involved in mRNA decay. EDC4 serves as a key scaffold for the decapping complex within P-bodies. Here, we demonstrate that N4BP1 interacts with EDC4, as well as with DCP1A, DCP2, and XRN1 – key components of 5′-cap hydrolysis. Endogenous N4BP1 colocalizes with EDC4 in P-bodies, requiring both of its KH domains. Structural analysis revealed that N4BP1 contains a type-I KH fold but lacks the canonical GXXG motif required for single-stranded RNA binding. Deletion or mutation of KH domains non-canonical GXXG motifs disrupts the N4BP1-EDC4 complex.
N4BP1 reduces HIV-1 transcript levels independently of its P-body localization or association with decapping components, implying the involvement of other host factors in regulating viral mRNAs. Similarly, for N4PB1-dependend negative regulation of endogenous transcripts in HaCaT keratinocytes, EDC4 is not essential. For both, HIV-1 and endogenous transcripts, the reduction is associated with the presence of the PIN domain.

ANSWERS TO THE REVIEWER #2:

We would like to sincerely thank the Reviewer for all the questions and comments. We have conducted additional experiments, which we hope will help clarify any doubts and improve the scientific message.

1. Clarify dependence on the 5′-to-3′ decay axis
Co-transfect catalytic-inactive CNOT7 or CNOT8 and assess whether N4BP1-mediated repression of HIV-1 or a standard reporter is attenuated as a readout for deadenylation dependence. Test a catalytic-inactive DCP2 or short-term XRN1 inhibition to see whether the N4BP1 effect is rescued, addressing decapping and 5'-3' exonucleolysis. Sensitivity at any node would support pathway-level coupling.

First of all we would like to thank for all questions and suggestions. To answer this question we performed additional experiment and added the following text to the subsection: "**N4BP1 regulates viral mRNAs independently of P-bodies"** of the Results in the manuscript (page 10 in the manuscript, lines 324-344):

To check, whether the mechanism of viral mRNA repression is deadenylation or decapping dependent we performed experiments with expression of key proteins for those processes. Although we did not detect CNOT7 as a direct interaction partner of N4BP1 in N4BP1 immunoprecipitates (MS results), we examined whether this protein, which is responsible for the deadenylation process, plays a significant role in the N4BP1-dependent negative regulation of HIV-1 pol mRNA levels. To directly address this point, we co-expressed wild-type N4BP1 with either wild-type or catalytically inactive components of the CNOT/DCP2 - deadenylation/decapping complex and monitored HIV-1 mRNA levels expressed from psPAX2 vector. In HEK293T cells transfected with psPAX2, we introduced either CNOT7-GFP (wild-type or catalytic mutant (D40A, E42A) or DCP2–GFP (wild-type or catalytic mutant (E148Q) with or without co-expression of wild-type N4BP1. Immunoblot analysis confirmed robust

*expression of all tested proteins, including WT and D623N N4BP1, DCP2-GFP and CNOT7-GFP under all relevant conditions, with comparable loading as indicated by β-actin (**Fig. 6D**).*

*We then quantified HIV-1 pol mRNA levels expressed from psPAX2 vector by qRT-PCR. Ectopic expression of N4BP1 (red bars) led to a significant reduction in HIV-1 pol mRNA levels in control samples as well as under co-expression conditions with wild-type CNOT7 (**Fig. 6E**).*

*Notably, the HIV-1 pol mRNA levels were similar in control cells expressing the mutant CNOT and in cells co-expressing N4BP1 with the mutant form of CNOT7. However, they were lower than in the corresponding control cells: empty or expressing only N4BP1 (**Fig. 6E**). In contrast, co-expression of N4BP1 with wild-type DCP2 or its catalytically inactive mutant did not affect the HIV-1 pol mRNA levels (**Fig. 6E**). These data suggest a decapping-independent mechanism of HIV-1 pol mRNA down-regulation.*

Fig. 6 of the manuscript has been changed as follows:

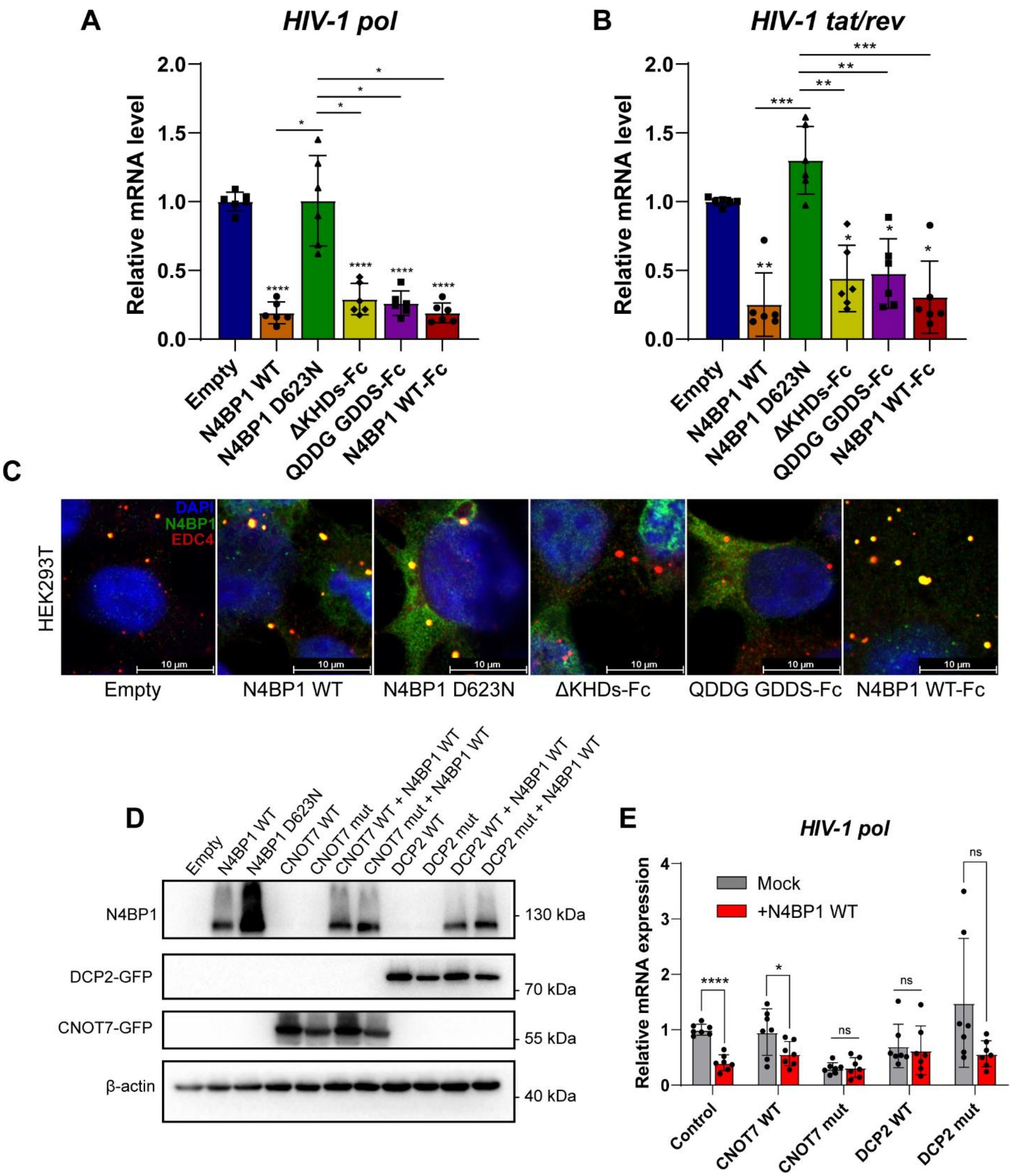

Fig. 1 (Figure 6 of the manuscript). KH domains of N4BP1 are not required for degradation of HIV-1 transcripts. A. Quantitative RT-PCR analysis of *HIV-1 pol* gene expression in HEK293T cells co-transfected with *pspax2* and the indicated N4BP1 expression vectors at 48 h. Statistical analysis was performed using Brown–Forsythe and Welch's ANOVA: F(5, 8.77) = 39,32; $P < 0.0001$. Significant differences were observed between control and active variants: empty vs N4BP1 WT $t(9,84) = 18,84$, $P < 0,0001$, empty vs ΔKH-Fc $t(8,24) = 12,98$, $P < 0,0001$, empty vs QDDG GDDS-Fc $t(9,44) = 16,04$,

P < 0,0001, empty vs N4BP1 WT-Fc *t*(9,99) = 19,84, *P* < 0,0001. N4BP1 D623N vs N4BP1 WT *t*(5,57) = 5,88, *P* = 0,0109, N4BP1 D623N vs ΔKH-Fc *t*(6,19) = 5,02, *P* = 0,024, N4BP1 D623N vs QDDG GDDS-Fc *t*(5,72) = 5,34, *P* = 0,0177, N4BP1 D623N vs N4BP1 WT-Fc *t*(5,47) = 5,92, *P* = 0,0178 **B.** Quantitative RT-PCR analysis of *HIV-1 tat/rev* gene expression under the same conditions as in panel B. ANOVA: F(5, 24.95) = 21.00; *P* < 0.0001. Empty vs N4BP1 WT *t*(5,16) = 7,89, *P* = 0,0049, empty vs ΔKH-Fc *t*(5,14) = 5,62, *P* = 0,0221, empty vs QDDG GDDS-Fc *t*(5,13) = 5,03, *P* = 0,0352, empty vs N4BP1 WT-Fc *t*(5,12) = 6,43, *P* = 0,0124, N4BP1 D623N vs N4BP1 WT *t*(9,95) = 7,60, *P* = 0,0002, N4BP1 D623N vs ΔKH-Fc *t*(9,99) = 6,09, *P* = 0,0015, N4BP1 D623N vs QDDG GDDS-Fc *t*(9,99) = 5,70, *P* = 0,0026, N4BP1 D623N vs N4BP1 WT-Fc *t*(9,96) = 6,75, *P* = 0,0007. Data represents SD from independent biological replicates. Cells co-transfected with an empty pcDNA3.0 vector and *pspax2* served as control. **C.** Immunofluorescence staining of N4BP1 (green) and EDC4 (red) in HEK293T cells 48 hours after co-transfection with *pspax2* and N4BP1 expression constructs. The identity of each ectopically expressed N4BP1 variant is indicated below the respective image. **D.** Western blot analysis confirming the expression of the indicated protein variants. **E.** quantitative RT-PCR analysis of HIV-1 *pol* mRNA levels under the indicated conditions. Paired t-test was used to calculate *P*-values for mock vs +N4BP1 WT comparisons. Control: *t*(6) = 9,95, *P* < 0,0001, CNOT WT: *t*(6) = 3,27, *P* = 0,0169, CNOT mut: *t*(6) = 0,24, *P* = 0,815, DCP2 WT: *t*(6) = 0,45, *P* = 0,67, DCP2 mut: *t*(6) = 2,24, *P* = 0,066. Experiments were performed in 7 independent biological replicates.

2.Identify the operative partner rather than assuming EDC4
Perform a small co-IP screen under RNase conditions across CCR4-NOT core and catalytic subunits CNOT1, CNOT2 or CNOT3, CNOT6 or CNOT6L, CNOT7 or CNOT8, and CNOT9 to CNOT11. Include common P-body bridging factors such as EDC3, DDX6, Lsm14A and PatL1. RNase-resistant associations would be strong candidates for a protein bridge worth validating.

We performed additional experiments to further evaluate whether the association between N4BP1 and the decapping complex is maintained under RNase-treated conditions, thereby minimizing potential RNA-mediated interactions. In line with the Reviewer's suggestion, we included benzonase during N4BP1 immunoprecipitation (**Fig. 2A B**) and performed an additional mass spectrometry analysis. Besides core components of the decapping complex (EDC4, DCP1A, EDC3, and DCP2) MS data revealed several ribosomal proteins (RPLP2, RPS3, RPS8, and RPS3A) as well as PATL1 but not components of CCR4-NOT complex. However, based on our pull-down data (**Suppl. Table 2**), PATL1 and CNOT7 were detected in IP fraction also in the sample where Fc tagged N4BP1 was not expressed (as shown below), thus we conclude such result as non-specific (**Fig. 2C**).

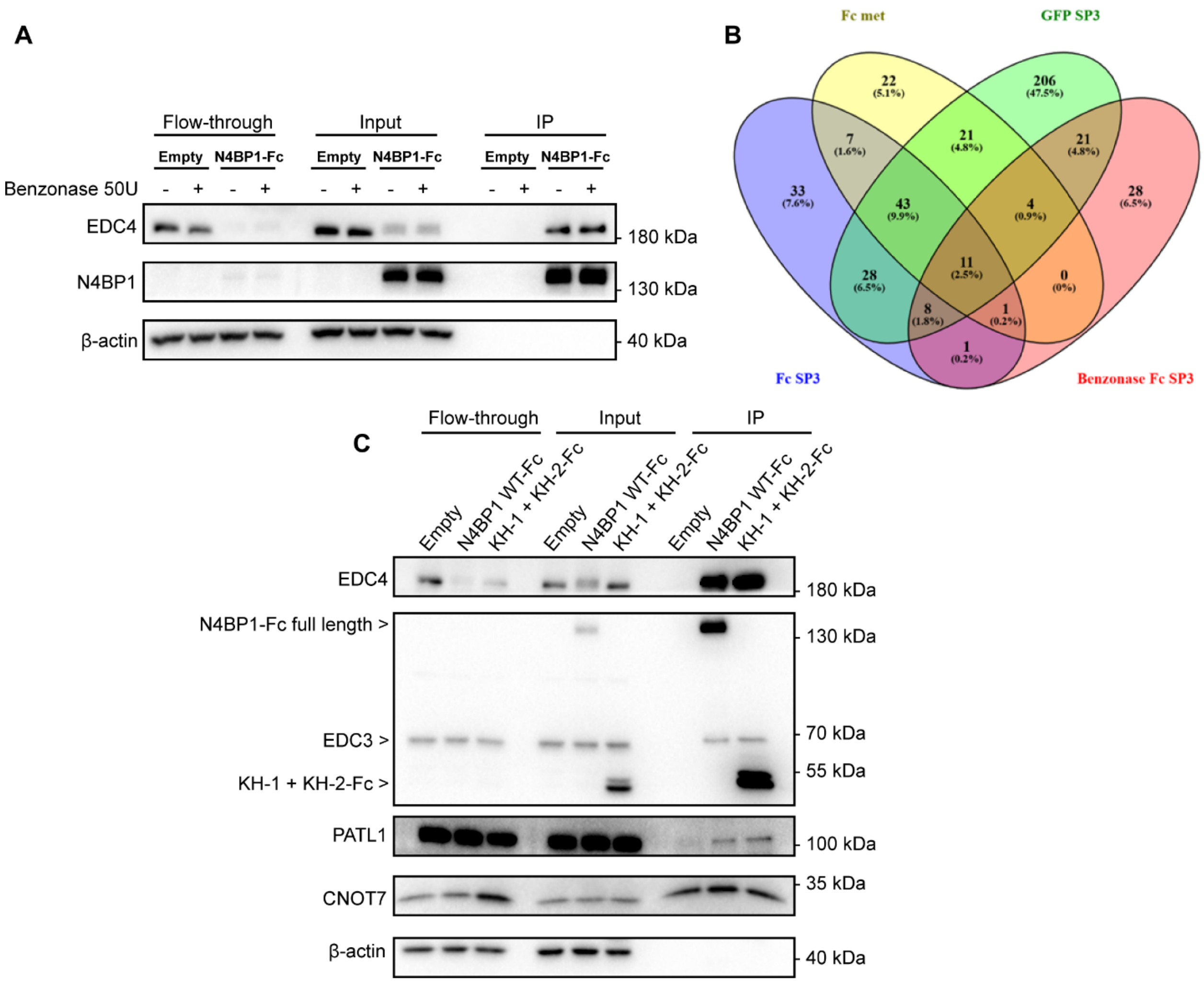

Fig. 2. Benzonase treatment does not abolish the interaction between N4BP1 and EDC4. A. Western blot analysis of samples treated with benzonase prior to immunoprecipitation. **B.** Venn diagram illustrating number of proteins identified by mass spectrometry under all tested conditions. **C.** Immunoprecipitation assay of full length N4BP1 and its tandem KH domain respectively, followed by western blot analysis.

On the basis of obtained results we changed the title of the subsection: "**RNA is required for the interaction between N4BP1 and EDC4" into "RNA molecule may not be necessary for stabilizing the interaction between N4BP1 and EDC4**". We also modified the text and added more information to the main text of the manuscript based on obtained results (page 9 in the manuscript, lines 289-302):

To further investigate whether the N4BP1-EDC4 complex is stabilized by RNA molecules, we performed immunoprecipitation experiments using HEK293T cell lysates pretreated with RNase A for 0.5 and 2 h. The N4BP1-EDC4 complex was then isolated using N4BP1-Fc as bait. Interestingly, we observed a time-dependent decrease in EDC4 levels in the immunoprecipitates in both untreated and RNase A - treated samples relative to the control

(0 h). However, this effect was more pronounced in the RNase A - treated samples. At the 2-hour time point, the difference between samples with and without RNase A was statistically significant (Fig. 5A, B). These results suggest that RNA may contribute to the interaction between N4BP1 and EDC4.

*To further examine this observation, we performed an additional experiment in which samples were treated with benzonase, followed by mass spectrometry analysis. We identified 11 proteins common to all N4BP1 pull-down datasets (Fig. 1C), including the benzonase-treated sample, indicating that their association with N4BP1 is largely independent of RNA-mediated interactions (**Fig. S8, Suppl. Table 2**). Proteins whose interaction with N4BP1 remained unaffected by benzonase treatment included core components of the decapping complex, such as EDC4, DCP1A, EDC3, and DCP2.*

Fig. 5 in the manuscript has been changed to show Western blot analysis of samples treated with benzonase prior to immunoprecipitation as **Fig. 5C**.

Supplementary Table 2 has been updated with IP-MS results after benzonase treatment.

Supplementary Fig. S8 has been added to show Venn diagram illustrating number of proteins identified by mass spectrometry under all tested conditions including benzonase treated sample.

3. Extend N4BP1 domain-function mapping beyond KH
Add targeted truncations or point mutants in the N-ter, the central low-complexity region, and the C-ter. Test interaction and localization with candidates emerging from the screen above to separate a localization platform role from interfaces that support functional partners.

We would like to thank the Reviewer for this insightful comment and agree that introducing point mutations within individual domains would provide valuable additional insight. Trying to find the region responsible for interaction of N4BP1 with EDC4, we constructed a panel of N4BP1 mutants with deletions, including: (i) the central intrinsically disordered region (IDP), (ii) the UBA domain, (iii) the NYN/PIN RNase domain and (iv) the C-terminal CoCUN ubiquitin-binding domain, as well as the N-terminal KH region (including deletion of residues 1–57 and deletion of both KH1 and KH2) (**Fig. 3**). Co-immunoprecipitation experiments with co-expressed EDC4 showed that deletion of the central low-complexity region, the NYN domain, the UBA domain, or the C-terminal CoCUN domain did not disrupt N4BP1-EDC4 complex formation. In contrast, removal of the N-terminal KH region (either the first 57 amino acids or the entire tandem KH module) completely abolished the interaction with EDC4.

Consistent with the biochemical data, immunofluorescence analyses revealed that deletion of the KH domains prevented localization of N4BP1 to EDC4-positive granules, resulting in

diffuse cytoplasmic staining. Together, these data support a model in which the N-terminal KH region serves as the primary localization platform for recruitment of N4BP1 to P-body via association with EDC4, whereas other domains contribute to catalytic activity (NYN) or to additional regulatory interactions (e.g. ubiquitin binding) that are not essential for EDC4 binding.

In addition, to show that KH domains are key for association with EDC4, we generated expression constructs encoding the tandem KH domain, as well as the individual KH1 and KH2 domains, and established a pull-down system to test their interaction profiles. Fc-tag-based pull-down experiments demonstrated that, despite the multidomain architecture of N4BP1, the N-terminal region containing the tandem KH domain is sufficient to independently mediate association with EDC4 and the decapping core complex (**Fig. 3A, B**). On the other hand, EDC4 failed to co-immunoprecipitate with N4BP1's single KH-1 or KH-2 domains (**Fig. 3A, B**). These results extend our previous observation based on mutagenesis of the GXXG binding motifs in KH domains.

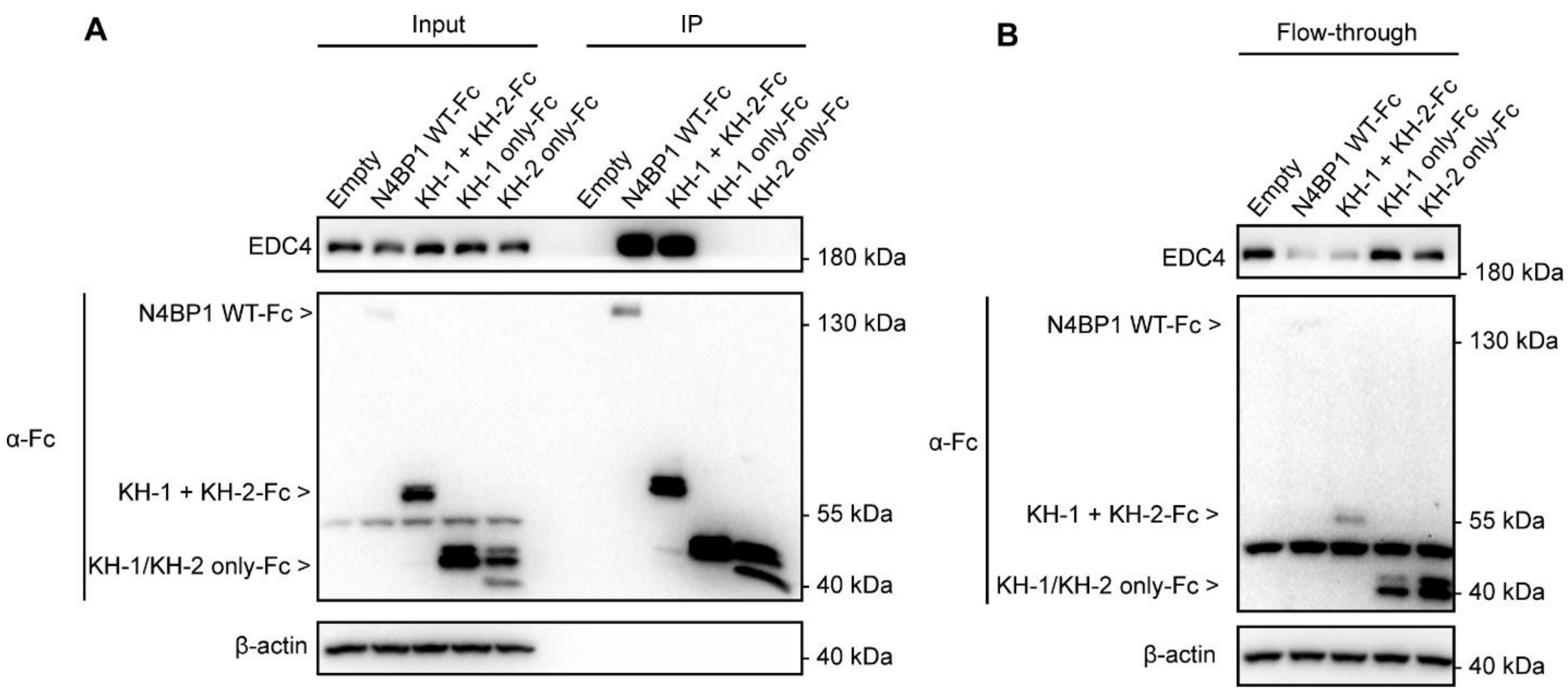

Fig. 3 (Fig. S3 in the Supplementary information): Analysis of the impact of the KH domains of N4BP1 on its association with the decapping complex, represented by EDC4. A. Pull-down assay of full length N4BP1 and its N-terminal fragments (tandem KH domains, KH1 and KH2), followed by western-blot analysis. **B.** Western blot analysis of flow-through fractions from the pull-down assay of N4BP1s fragments. Presented results are representative of 3 biological replicates.

In the current version of **the manuscript, the Fig. 3** has been expanded to show that both KH domains of N4BP1 are required for association with EDC4, whereas neither domain alone is sufficient for this interaction as shown below. In addition, the following text to the subsection: "**The KH domain is mandatory for N4BP1 interaction with EDC4**" of the Results was added (page 7 in the manuscript, lines 220-227):

To further determine whether the tandem KH domains are sufficient for EDC4 binding, we performed pull-down analyses using constructs expressing either the tandem KH domains or

*the individual KH1 and KH2 domains. These experiments demonstrated that the tandem KH domains are sufficient for association with EDC4 as well as with components of the decapping complex (**Fig. 3F,G, Suppl. Fig. S3**). Moreover, a substantial fraction of endogenous EDC4 was recovered from cells ectopically expressing either full-length N4BP1 or the tandem KH domains, as evidenced by reduced levels of EDC4 in the flow-through fraction following pull-down (**Fig. 3G, Suppl. Fig. S3**). This indicates efficient capture of endogenous EDC4 by the overexpressed tandem KH domains of N4BP1.*

4. Provide a minimal endogenous validation set
Generate an N4BP1 knockout and perform RNA-seq to nominate two to three upregulated, biologically plausible targets. Measure half-life by actinomycin D time course or a simplified 4sU-pulse to confirm stabilization upon knockout. Conduct complementation with WT, ΔKH, and the RNase-dead D623N. Rescue by WT but not by D623N, with partial rescue by ΔKH, would extend the HIV-1 findings to endogenous transcripts. As a low-burden link to RNA substrates, RIP-qPCR for these candidates would suffice without high-throughput mapping.

This was an excellent suggestion to examine changes in the levels of endogenous transcripts. We performed additional experiments and in the main text of the manuscript we added **Fig. 7** and subsection: "**Regulation of eukaryotic transcripts by N4BP1**" (page 11 in the manuscript, lines 347-376):

*To determine whether N4BP1 regulates also eukaryotic endogenous transcripts we performed transcriptome profiling in HaCaT keratinocytes upon ectopic expression of wild-type N4BP1, which allowed us to identify endogenous mRNAs whose abundance decreases in response to N4BP1 expression. RNA-seq analysis revealed several downregulated transcripts, including FREM2, ETV1 and NOTCH3, which we prioritized as plausible N4BP1-sensitive targets based on their expression (**Fig. 7A**). Gene ontology (GO) analysis of biological processes revealed enrichment of differentially expressed genes (DEGs) involved in vascular development, highlighting 42 genes in this category (**Fig. 7B**). Other prominent processes affected included cell adhesion, cell differentiation, and intracellular transport. Complementary GO cellular component analysis linked these changes to genes associated with desmosomes, intercellular junctions, extracellular organelles, and vesicular transport structures (**Fig. 7C**). These data suggest a role for N4BP1 in modulating cellular connectivity and extracellular matrix dynamics, key for tissue organization and vascular function.*

*We then validated selected candidates by qRT-PCR and assessed their dependence on N4BP1 NYN catalytic activity and P-body localization. Specifically, we compared the effects of overexpressing wild-type N4BP1, N4BP1 with deleted KH domains (ΔKHDs N4BP1), N4BP1 with mutations in KH domains (QDDG GDDS N4BP1) and the RNase-inactive D623N mutant. In these experiments, wild-type N4BP1 significantly reduced FREM2, ETV1 and NOTCH3 mRNA levels, whereas the D623N mutant failed to do so (**Fig. 7D**). Most importantly, transcript levels in cells ectopically expressing N4BP1 mutants lacking the KH domains or carrying mutations within these domains were significantly lower than in cells expressing the*

RNase-deficient PIN/NYN mutant of N4BP1 (D623N), and were comparable to the levels observed in cells ectopically expressing wild-type N4BP1. Additionally, we performed an experiment in which cells with ectopic expression of N4BP1 had silenced expression of EDC4 with specific siRNAs. As shown in ***Fig. 7E****, the transcript levels of FREM2, ETV1, and NOTCH3 were significantly reduced compared to cells treated with scrambled siRNA, indicating that N4BP1 degrades transcripts when EDC4 is silenced.*

These results mirror our findings for transcript encoded by psPAX2 (HIV-1 pol and tat/rev mRNAs) and support the conclusion that the NYN domain is essential for N4BP1-mediated repression of selected both viral and endogenous transcripts represented by FREM2, ETV1 and NOTCH3 mRNAs, while the KH domains, which are required for the association of N4BP1 with EDC4 and localization in the P-bodies, are largely dispensable for the degradation of the

analyzed mRNA.

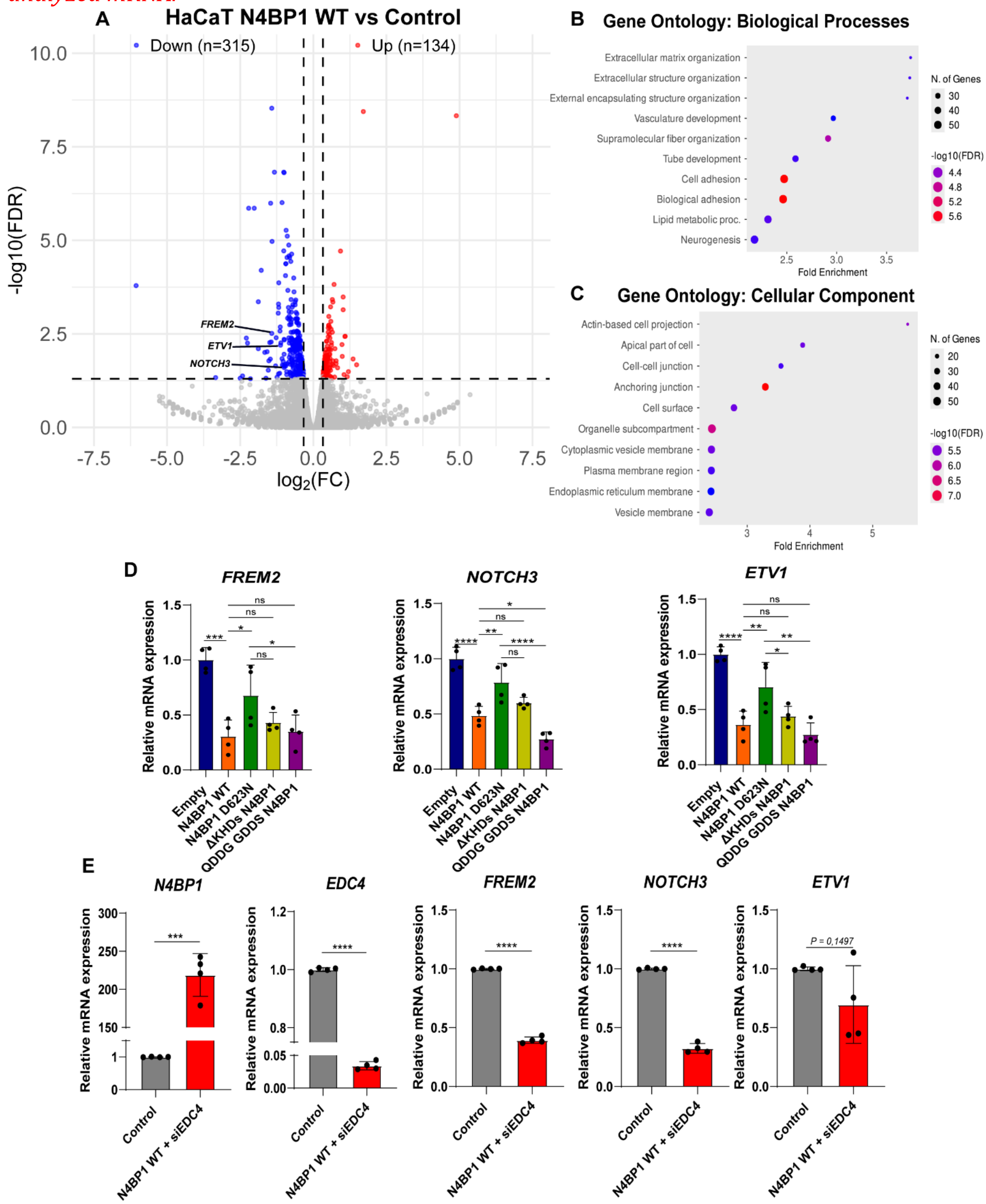

Fig. 4 (Fig. 7 in the manuscript). RNA-seq of HaCaT cells upon exogenous expression of wild-type (WT) N4BP1. A. Volcano plot showing differentially expressed genes upon ectopic expression of WT N4BP1. **B.** Gene Ontology analysis of Biological Process terms enriched among significantly

downregulated genes. **C.** Gene Ontology analysis of Cellular Components terms enriched among significantly downregulated genes. **D.** Q-RT-PCR validation of selected transcripts used to confirm RNA-seq results. Statistical analysis was performed using one-way ANOVA: F(4, 8,61) = 11,73, P = 0,0015. *FREM2:* N4BP1 WT vs Empty P = 0,0001, N4BP1 WT vs N4BP1 D623N P = 0,023, N4BP1 WT vs ΔKHDs N4BP1 P = 0,68, N4BP1 WT vs QDDG GDDS N4BP1 P = 0,98, N4BP1 D623N vs ΔKHDs N4BP1 P = 0,16, N4BP1 D623N vs QDDG GDDS N4BP1 P = 0,049. *NOTCH3*: N4BP1 WT vs Empty P < 0,0001, N4BP1 WT vs N4BP1 D623N P =0,0035, N4BP1 WT vs ΔKHDs N4BP1 P = 0,37, N4BP1 WT vs QDDG GDDS N4BP1 P = 0,038, N4BP1 D623N vs ΔKHDs N4BP1 P = 0,075, N4BP1 D623N vs QDDG GDDS N4BP1 P < 0,0001. *ETV1:* N4BP1 WT vs Empty P < 0,0001, N4BP1 WT vs N4BP1 D623N P = 0,0084, N4BP1 WT vs ΔKHDs N4BP1 P = 0,83, N4BP1 WT vs QDDG GDDS N4BP1 P = 0,74, N4BP1 D623N vs ΔKHDs N4BP1 P = 0,0412, N4BP1 D623N vs QDDG GDDS N4BP1 P = 0,0012. Experiment was performed in 4 independent biological replicates. **E.** Q-RT-PCR of selected transcripts upon simultaneous ectopic expression of N4BP1 WT and silencing of EDC4 using specific siRNA. Paired t-test was used to calculate P-values for Control vs N4BP1 WT + siEDC4 groups. *N4BP1* $t(3)$ = 15,5, P = 0,006, *EDC4* $t(3)$ = 511,7, P < 0,0001, *FREM2* $t(3)$ = 47,49, P < 0,0001, *NOTCH3* $t(3)$ = 38,37, P < 0,0001, *ETV1* $t(3)$ = 1,926, P = 0,1497. Experiment was performed in 4 independent biological replicates.

Minor:

Adopt a cautious description of the N4BP1–EDC4 relationship and refer to it as RNA mediated or RNA stabilized until RNA bridging and third-party proteins are excluded. Introduce an alternative model in the Discussion that N4BP1 may act through CCR4-NOT or other P-body factors rather than relying on P-body localization per se. Present the quantification of P-body colocalization more clearly in the main text and include input and integrity controls for RNase and knockdown conditions. If space permits, add a non-viral reporter such as a tethering assay or a representative 3' UTR to show that NYN dependence and KH dispensability generalize beyond the viral system.

Using RNase A or benzonase, we obtained contrasting results. As described in point 2 and in the manuscript, the experiment with RNase A did not yield clear conclusions, because even incubation of samples prepared for immunoprecipitation - when pre-incubated at 37 °C in the same way as the RNase-treated samples - led to a reduction of EDC4 in the immunoprecipitates. This effect was more pronounced in the samples incubated with RNase. After two hours of incubation, the EDC4 signal in the western blot was almost undetectable.

In contrast, the addition of benzonase prior to immunoprecipitation did not affect the composition of the complex, and decapping proteins were still detected in N4BP1 immunoprecipitates. Therefore, our interpretation regarding the role of RNA in the interaction with EDC4 has been substantially softened in the revised version of the manuscript.

As described and shown in the point above and in the current version of the manuscript, the degradation of selected eukaryotic transcripts by N4BP1 depends on the active PIN

domain rather than the KH domain, and the presence of EDC4 is not essential for the efficient nucleolytic activity of N4BP1.

Because we performed additional experiments. the Abstract has been updated accordingly to reflect the newly generated results. The new part of the abstract is written in Italics (page 2 in the manuscript, lines 60-71):

Processing bodies (P-bodies) are cytoplasmic, non-membrane-bound structures involved in mRNA decay. EDC4 serves as a key scaffold for the decapping complex within P-bodies. Here, we demonstrate that N4BP1 interacts with EDC4, as well as with DCP1A, DCP2, and XRN1 – key components of 5′-cap hydrolysis. Endogenous N4BP1 colocalizes with EDC4 in P-bodies, requiring both of its KH domains. Structural analysis revealed that N4BP1 contains a type-I KH fold but lacks the canonical GXXG motif required for single-stranded RNA binding. Deletion or mutation of KH domains non-canonical GXXG motifs disrupts the N4BP1-EDC4 complex.
N4BP1 reduces HIV-1 transcript levels independently of its P-body localization or association with decapping components, implying the involvement of other host factors in regulating viral mRNAs. Similarly, for N4PB1-dependend negative regulation of endogenous transcripts in HaCaT keratinocytes, EDC4 is not essential. For both, HIV-1 and endogenous transcripts, the reduction is associated with the presence of the PIN domain.

ANSWERS TO THE REVIEWER #3:

We would like to sincerely thank the Reviewer for all the questions and comments. We have conducted additional experiments, which we hope will help clarify any doubts and improve the scientific message.

1. The authors show that endogenous N4BP1 interacts with EDC4 in cytoplasmic granule (Fig. 1G). However, the picture shows that N4BP1 is mostly localized in the nucleus with the granular pattern. The authors should precisely describe the results. If so, there are discrepancy between endogenous N4BP1 staining (Fig. 1G) and GFP-tagged one (Fig. 2A). The authors need to demonstrate if the expression pattern of N4BP1-GPF is not the overexpression artifact.

To be sure that our results are not artificial, we repeated endogenous N4BP1 immunofluorescence staining in HeLa cells using an another anti-N4BP1 antibody (anti-N4BP1, Cell Signalling, E8H5E). These experiments revealed a clear cytoplasmic granular pattern of endogenous N4BP1 that colocalizes with EDC4-positive P-bodies. While some

nuclear staining is also observed, the prominent signal overlapping with EDC4 is in cytoplasmic granules. Representative images from these new experiments are included in (**Fig. 1**). In experiments involving ectopically expressed N4BP1-GFP, the signal was detected directly via the GFP tag, thereby avoiding background associated with the anti-N4BP1 antibodies used to stain endogenous N4BP1. This may also account for the slightly different subcellular localization patterns observed between these two approaches.

Additional control experiments were performed to ensure that N4BP1-GFP localization is not an overexpression artifact and are presented in Supplementary Information (**Fig. S2**). We compared cells expressing N4BP1-GFP with cells expressing GFP alone. GFP alone exhibited a diffuse distribution with prominent nuclear accumulation and no specific colocalization with EDC4. In contrast, N4BP1-GFP formed discrete cytoplasmic granules that extensively overlapped with EDC4 and other decapping markers. This localization pattern was consistent across multiple cell lines, including HeLa, HaCaT and HEK293T cells.

Together, these data support the conclusion that both endogenous and GFP-tagged N4BP1 localize to P-bodies, and that the N4BP1-GFP pattern is not an overexpression artifact but reflects the physiological recruitment of N4BP1 to these granules.

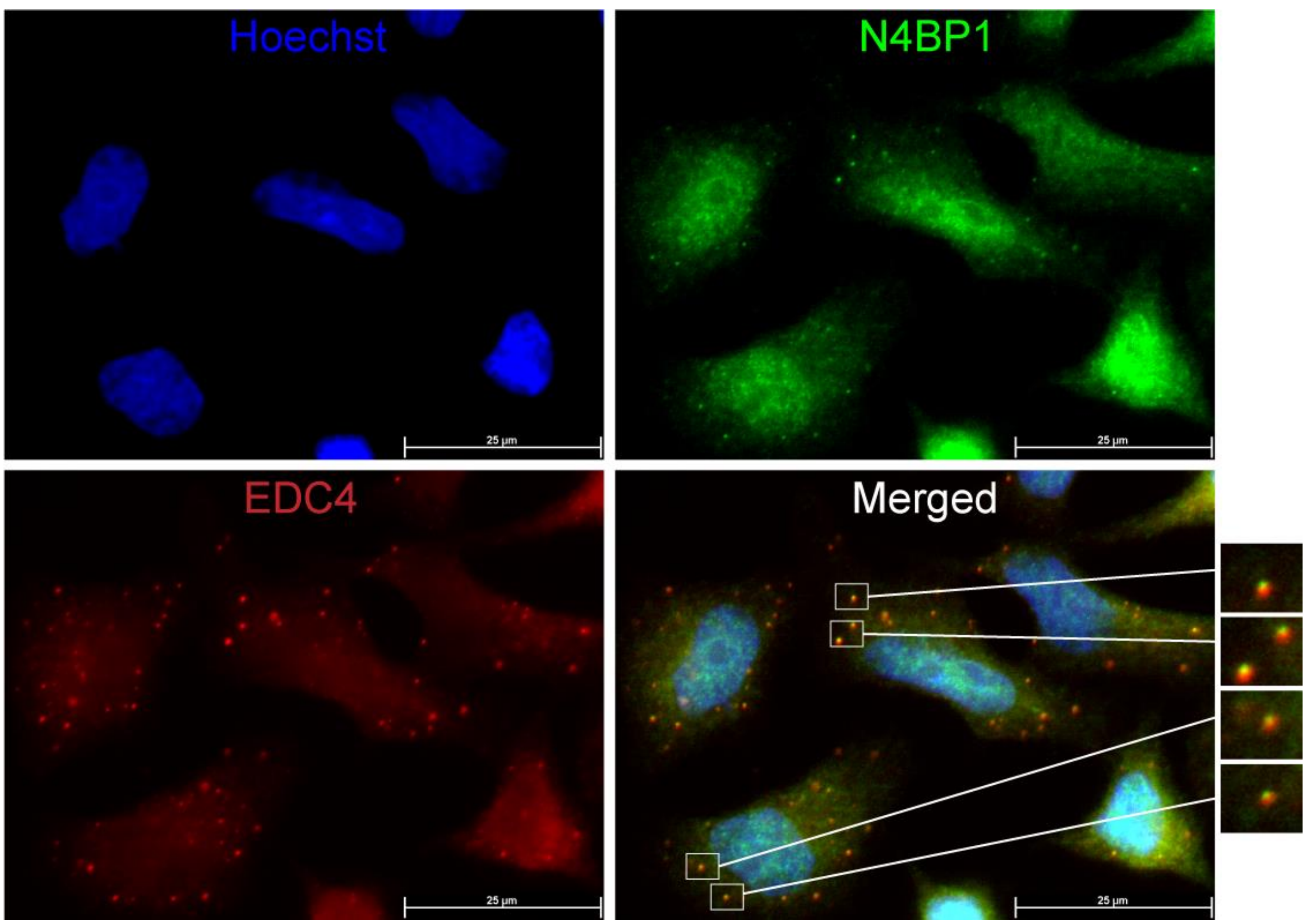

Fig. 1. Immunofluorescent staining of endogenous N4BP1 and EDC4 in HeLa cells using specific primary antibodies (anti-N4BP1, Cell Signalling, E8H5E; anti-EDC4, Abcam, ab72408). Images are representatives of 3 independent biological replicates.

2. Also, the authors should validate the anti-N4BP1 antibody specifically precipitate this protein.

To address this, we attempted to perform co-immunoprecipitation of endogenous N4BP1 and EDC4 using the commercial anti-N4BP1 antibody (Cell Signalling, E8H5E) and anti-EDC4 antibody (Abcam, ab72408). While we were able to observe a band corresponding to N4BP1 after anti-N4BP1 IP (**Fig. 2A**), we observed that a band at the expected molecular weight of EDC4 was present even when the membrane was incubated only with secondary HRP-conjugated antibodies (**Fig. 2B**), indicating that this signal was not specific to the primary anti-EDC4 antibody in this setup. This led us to conclude that our endogenous IP conditions with the available polyclonal antibodies were not sufficiently specific to reliably detect an N4BP1–EDC4 complex. These IP results are not included in the revised manuscript or supplementary materials due to insufficient specificity, but are presented here to demonstrate that we systematically tested and ruled out endogenous antibody-based IP as a reliable approach for this study.

In light of these observations, we chose to rely on tag-based immunoprecipitation approaches (N4BP1–Fc and N4BP1–GFP) for interaction studies. These systems allowed us to capture N4BP1 with high efficiency and low background using well-characterized affinity reagents (Protein G for Fc and GFP-Trap nanobodies), and to validate the presence of EDC4 and other decapping factors in N4BP1 complexes by Western blot. We show that both tagging strategies yield overlapping sets of interactors, including EDC4, DCP1A, DCP2 and XRN1 (**Fig. 2 in the manuscript**).

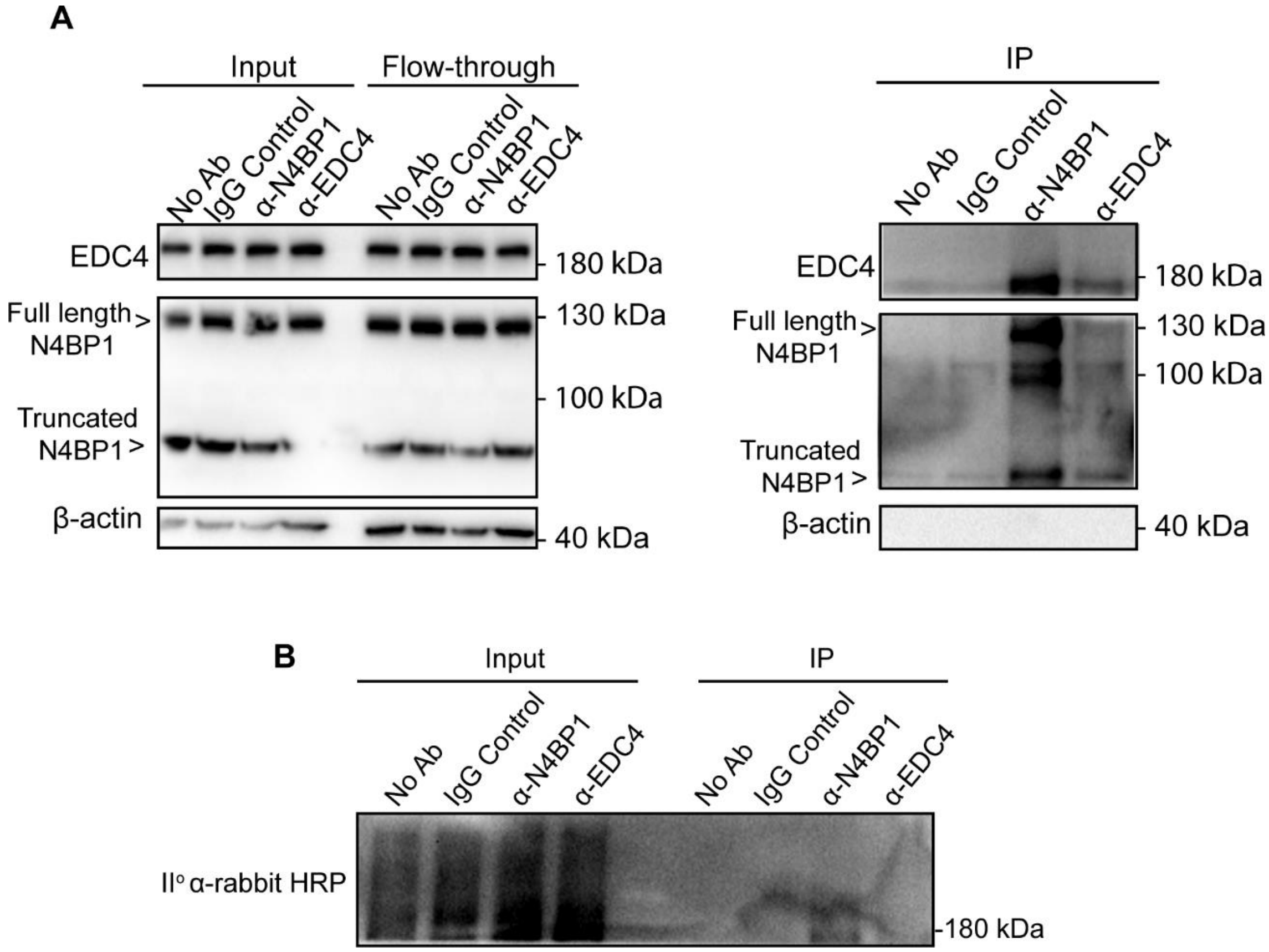

Fig. 2. Co-immunoprecipitation of N4BP1 and EDC4 using specific primary antibodies. A. Western blot analysis of immunoprecipitation samples obtained using specific antibodies anti-N4BP1 (Cell Signalling, E8H5E) and anti-EDC4 (Abcam, ab72408). **B.** Western blot analysis of immunoprecipitation samples detected with secondary anti-rabbit HRP-conjugated antibodies.

To further validate the specificity of one of the anti-N4BP1 antibodies (Cell Signalling, E8H5E) used for immunofluorescence, we performed staining in wild-type HEK293 cells and two independent N4BP1 knockout HEK293 cell lines (two clones). In wild-type cells, N4BP1 staining revealed cytoplasmic granules that colocalize with EDC4. In both N4BP1 knock-out lines, no cytoplasmic N4BP1 signal was detectable, confirming the antibody's specificity for endogenous N4BP1 (**Fig. 3**).

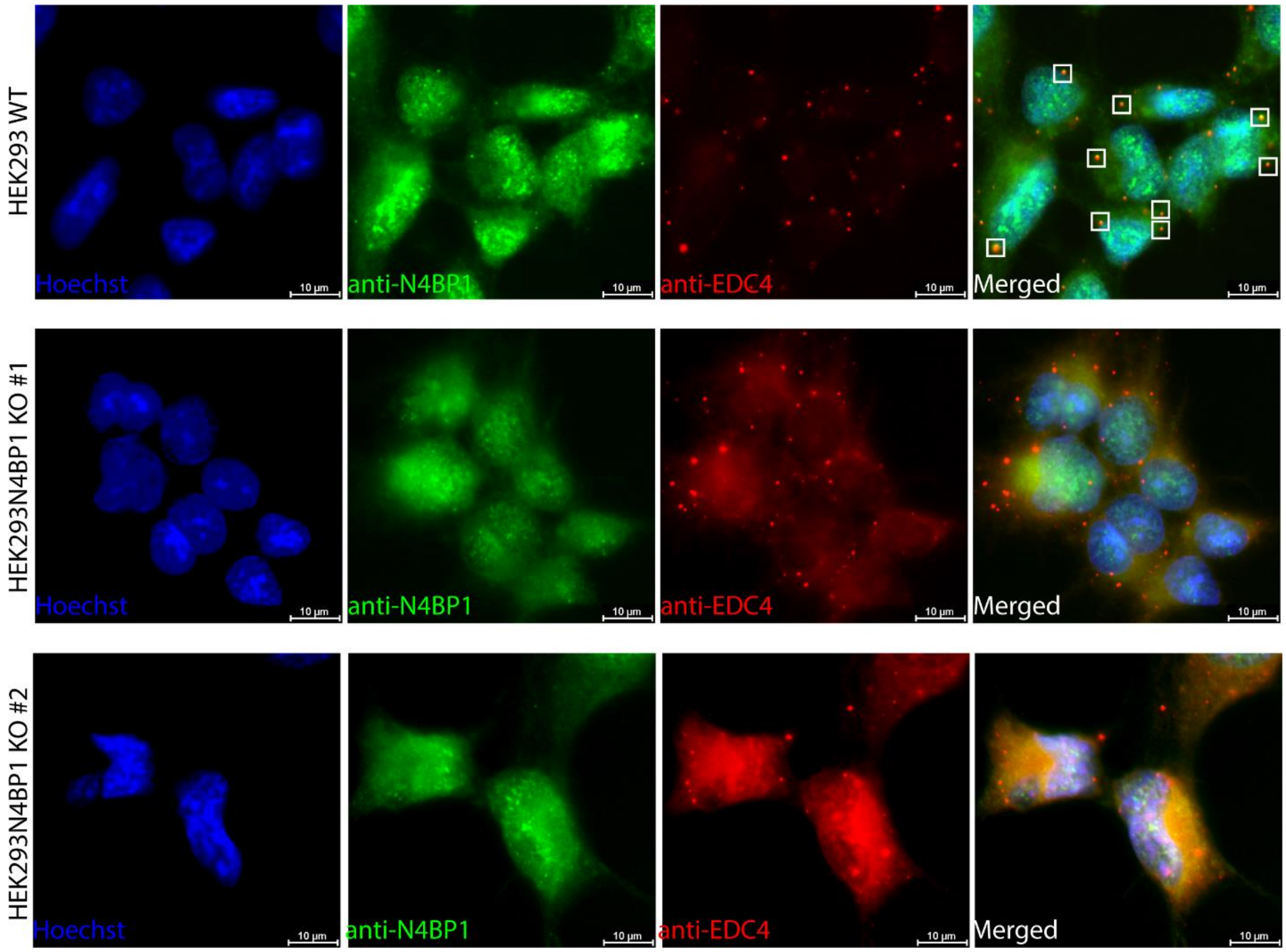

Fig. 3. Immunofluorescent staining of endogenous N4BP1 and EDC4 using specific primary antibodies (anti-N4BP1: Cell Signalling, E8H5E and anti-EDC4: Abcam, ab72408) in HEK293 WT cell line and two N4BP1-knockout cell lines. Images are representatives of 3 biological repetitions

3. Recent studies demonstrated that association of N4BP1 with ubiquitin suppresses canonical NF-kB activation by suppressing NEMO activation or activation of non-canonical IKKs. Do the N4BP1-EDC4 interaction contribute to this activity? Furthermore, they should explore whether ubiquitination influences N4BP1's interaction with EDC4 and P-bodies.

This is very interesting question, however, NF-κB signaling was not a primary focus of the present work, and we did not investigate regulation of this complex by ubiquitination, primarily due to the lack of overrepresentation of ubiquitin or ubiquitin ligases in N4BP1 pull-down samples analyzed by MS (**Suppl. Table 2**). We fully agree that the potential link between N4BP1 function in P-bodies and NF-κB-dependent cytokine regulation represents an important question for future investigation. Addressing this issue will likely require more physiologically relevant immune cell systems. In this regard, we are currently

developing a new project to examine the role of N4BP1 in Jurkat cells; however, these plans are still preliminary and therefore beyond the scope of the present manuscript.

4. The structural study shown in Fig. 4 does not contribute to the study's conclusions and should be presented in a separate paper. The absence of the GXXG motif could be concluded without structural information.

We agree with the Reviewer that the presence of the conserved GXXG motif could have been inferred from sequence homology and structural prediction. However, the value of the structural work extends beyond confirmation of this motif. Crystallographic studies provide direct experimental evidence that the tandem KH domain of N4BP1 adopts a defined three-dimensional fold. In the absence of structural data we could only assume that this domain would fold similarly to other homologous KH domains. Importantly, we determined the crystal structure of the human N4BP1 tandem KH domain, which provides a valuable framework for future molecular modeling and, potentially, for structure-guided inhibitor design. We therefore consider the structural characterization itself to represent a substantial scientific contribution.

Based on discussion of the structure, we performed additional studies on the properties of the KH tandem domain, which ultimately proved the impact of these domains on association with EDC4. Accordingly, **Figure 4 in the manuscript** has been expanded to include new biochemical data demonstrating that the tandem KH domains of N4BP1 are sufficient for EDC4 binding (**Fig. 4 in the current file**) while results showing that the individual KH domains alone are not sufficient to mediate this interaction are shown in Supplementary Information (**Fig. S3)**. The following text was added to the subsection: "**The KH domain is mandatory for N4BP1 interaction with EDC4" of the Results** (page 7 in the manuscript, lines 220-227)**:**

*To further determine whether the tandem KH domains are sufficient for EDC4 binding, we performed pull-down analyses using constructs expressing either the tandem KH domains or the individual KH1 and KH2 domains. These experiments demonstrated that the tandem KH domains are sufficient for association with EDC4 as well as with components of the decapping complex (**Fig. 3F,G, Suppl. Fig. S3**). Moreover, a substantial fraction of endogenous EDC4 was recovered from cells ectopically expressing either full-length N4BP1 or the tandem KH domains, as evidenced by reduced levels of EDC4 in the flow-through fraction following pull-down (**Fig. 3G, Suppl. Fig. S3**). This indicates efficient capture of endogenous EDC4 by the overexpressed tandem KH domains of N4BP1.*

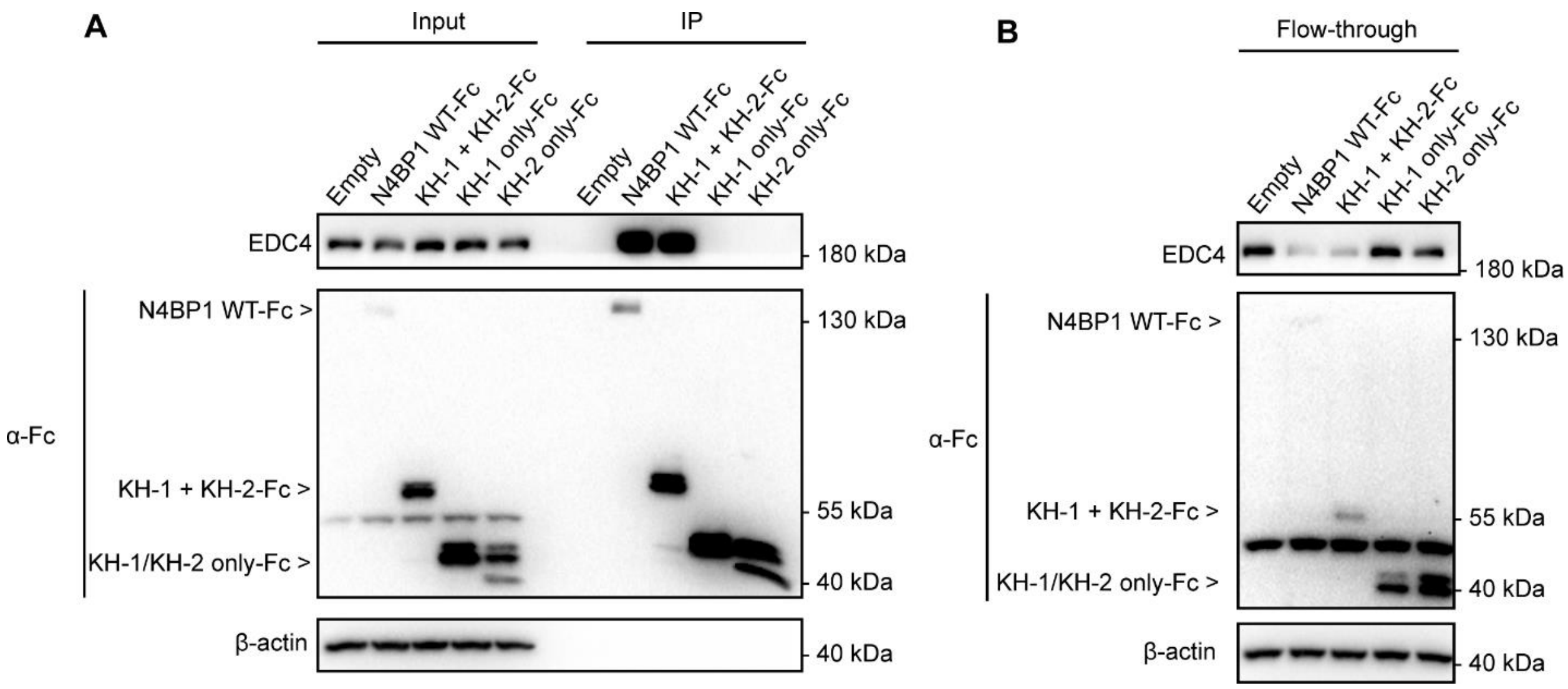

Fig. 4. (Fig. S3 in the Supplementary information): Analysis of the role of the N4BP1 KH domain in its association with the decapping complex represented by EDC4. A. Pull-down assay of full-length N4BP1 and its fragments (KH-tandem, KH1, and KH2), followed by western blot analysis. **B.** Western blot analysis of flow-through fraction from the pull-down assay of N4BP1s fragments. Experiments were performed in 3 independent biological replicates.

5. The biological meaning of the interaction between N4BP1, P-bodies, decapping enzymes (including EDC4), and mRNA degradation is not clear.

We agree with the Reviewer that the biological significance of the interaction between N4BP1, P-bodies, decapping enzymes (including EDC4), and mRNA degradation is not yet fully understood. As also indicated in our response to Reviewer comment 3, additional studies will be required to determine whether the interaction of N4BP1 with the decapping complex primarily contributes to mRNA degradation and/or mRNA storage within P-bodies. However, the present manuscript clearly establishes the mechanistic basis of the association between EDC4 and the tandem KH domain of N4BP1.

We performed transcriptome profiling in HaCaT keratinocytes upon ectopic expression of wild-type N4BP1, which allowed us to identify endogenous mRNAs whose abundance decreases in response to N4BP1 expression. RNA-seq analysis revealed several downregulated transcripts, including *FREM2*, *ETV1* and *NOTCH3*, which we prioritized as plausible N4BP1-sensitive targets based on their expression (**Fig. 5 in the current file, Fig. 7 in the manuscript**). We then validated chosen candidates by qRT-PCR and assessed their dependence on N4BP1 NYN catalytic activity and P-body localization. We added new figure and subsection to the Results (page 11 in the manuscript, lines 347-376):

"Regulation of eukaryotic transcripts by N4BP1"

To determine whether N4BP1 regulates also eukaryotic endogenous transcripts we performed transcriptome profiling in HaCaT keratinocytes upon ectopic expression of wild-type N4BP1, which allowed us to identify endogenous mRNAs whose abundance decreases in response to

*N4BP1 expression. RNA-seq analysis revealed several downregulated transcripts, including FREM2, ETV1 and NOTCH3, which we prioritized as plausible N4BP1-sensitive targets based on their expression (**Fig. 7A**). Gene ontology (GO) analysis of biological processes revealed enrichment of differentially expressed genes (DEGs) involved in vascular development, highlighting 42 genes in this category (**Fig. 7B**). Other prominent processes affected included cell adhesion, cell differentiation, and intracellular transport. Complementary GO cellular component analysis linked these changes to genes associated with desmosomes, intercellular junctions, extracellular organelles, and vesicular transport structures (**Fig. 7C**). These data suggest a role for N4BP1 in modulating cellular connectivity and extracellular matrix dynamics, key for tissue organization and vascular function.*

*We then validated selected candidates by qRT-PCR and assessed their dependence on N4BP1 NYN catalytic activity and P-body localization. Specifically, we compared the effects of overexpressing wild-type N4BP1, N4BP1 with deleted KH domains (ΔKHDs N4BP1), N4BP1 with mutations in KH domains (QDDG GDDS N4BP1) and the RNase-inactive D623N mutant. In these experiments, wild-type N4BP1 significantly reduced FREM2, ETV1 and NOTCH3 mRNA levels, whereas the D623N mutant failed to do so (**Fig. 7D**). Most importantly, transcript levels in cells ectopically expressing N4BP1 mutants lacking the KH domains or carrying mutations within these domains were significantly lower than in cells expressing the RNase-deficient PIN/NYN mutant of N4BP1 (D623N), and were comparable to the levels observed in cells ectopically expressing wild-type N4BP1. Additionally, we performed an experiment in which cells with ectopic expression of N4BP1 had silenced expression of EDC4 with specific siRNAs. As shown in **Fig. 7E**, the transcript levels of FREM2, ETV1, and NOTCH3 were significantly reduced compared to cells treated with scrambled siRNA, indicating that N4BP1 degrades transcripts when EDC4 is silenced.*

These results mirror our findings for transcript encoded by psPAX2 (HIV-1 pol and tat/rev mRNAs) and support the conclusion that the NYN domain is essential for N4BP1-mediated repression of selected both viral and endogenous transcripts represented by FREM2, ETV1 and NOTCH3 mRNAs, while the KH domains, which are required for the association of N4BP1 with EDC4 and localization in the P-bodies, are largely dispensable for the degradation of the analyzed mRNA.

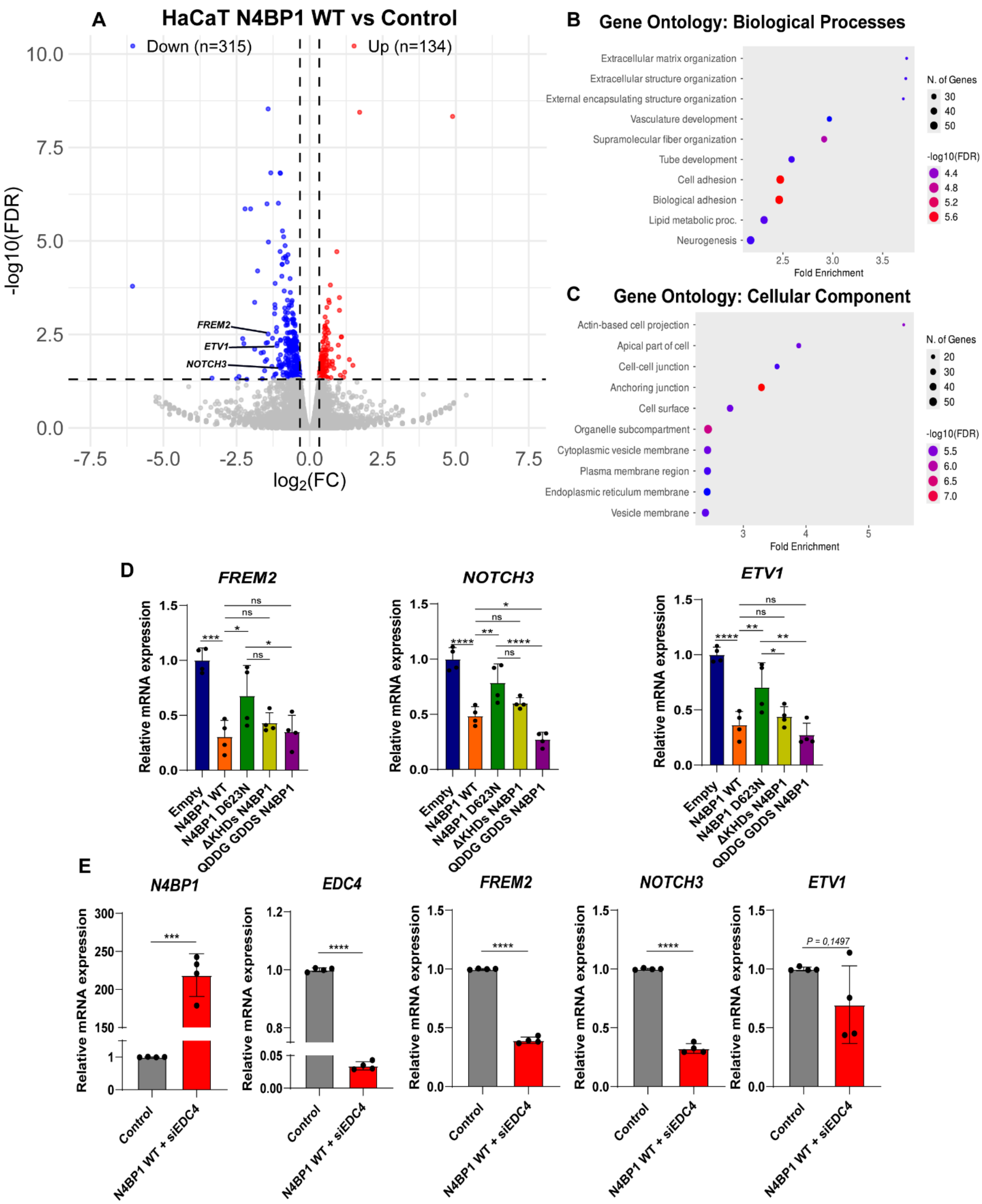

Fig. 5. (Fig. 7 in the main manuscript). RNA-seq of HaCaT cells upon exogenous expression of wild-type (WT) N4BP1. A. Volcano plot showing differentially expressed genes upon ectopic expression of WT N4BP1. **B.** Gene Ontology analysis of Biological

Process terms enriched among significantly downregulated genes. **C.** Gene Ontology analysis of Cellular Components terms enriched among significantly downregulated genes. **D.** Q-RT-PCR validation of selected transcripts used to confirm RNA-seq results. Statistical analysis was performed using one-way ANOVA: F(4, 8,61) = 11,73, *P* = 0,0015. *FREM2:* N4BP1 WT vs Empty *P* = 0,0001, N4BP1 WT vs N4BP1 D623N *P* = 0,023, N4BP1 WT vs ΔKHDs N4BP1 *P* = 0,68, N4BP1 WT vs QDDG GDDS N4BP1 *P* = 0,98, N4BP1 D623N vs ΔKHDs N4BP1 *P* = 0,16, N4BP1 D623N vs QDDG GDDS N4BP1 *P* = 0,049. *NOTCH3*: N4BP1 WT vs Empty *P* < 0,0001, N4BP1 WT vs N4BP1 D623N *P* =0,0035, N4BP1 WT vs ΔKHDs N4BP1 *P* = 0,37, N4BP1 WT vs QDDG GDDS N4BP1 *P* = 0,038, N4BP1 D623N vs ΔKHDs N4BP1 *P* = 0,075, N4BP1 D623N vs QDDG GDDS N4BP1 *P* < 0,0001. *ETV1:* N4BP1 WT vs Empty *P* < 0,0001, N4BP1 WT vs N4BP1 D623N *P* = 0,0084, N4BP1 WT vs ΔKHDs N4BP1 *P* = 0,83, N4BP1 WT vs QDDG GDDS N4BP1 *P* = 0,74, N4BP1 D623N vs ΔKHDs N4BP1 *P* = 0,0412, N4BP1 D623N vs QDDG GDDS N4BP1 *P* = 0,0012. Experiment was performed in 4 independent biological replicates. **E.** Q-RT-PCR of selected transcripts upon simultaneous ectopic expression of N4BP1 WT and silencing of EDC4 using specific siRNA. Paired t-test was used to calculate *P*-values for Control vs N4BP1 WT + siEDC4 groups. *N4BP1* $t(3)$ = 15,5, *P* = 0,006, *EDC4* $t(3)$ = 511,7, *P* < 0,0001, *FREM2* $t(3)$ = 47,49, *P* < 0,0001, *NOTCH3* $t(3)$ = 38,37, *P* < 0,0001, *ETV1* $t(3)$ = 1,926, *P* = 0,1497. Experiment was performed in 4 independent biological replicates.

Because we performed additional experiments, the Abstract has been updated accordingly to reflect the newly generated results. The new part of the abstract is written in Italics (page 2 in the manuscript, lines 60-71):

Processing bodies (P-bodies) are cytoplasmic, non-membrane-bound structures involved in mRNA decay. EDC4 serves as a key scaffold for the decapping complex within P-bodies. Here, we demonstrate that N4BP1 interacts with EDC4, as well as with DCP1A, DCP2, and XRN1 – key components of 5′-cap hydrolysis. Endogenous N4BP1 colocalizes with EDC4 in P-bodies, requiring both of its KH domains. Structural analysis revealed that N4BP1 contains a type-I KH fold but lacks the canonical GXXG motif required for single-stranded RNA binding. Deletion or mutation of KH domains non-canonical GXXG motifs disrupts the N4BP1-EDC4 complex.
N4BP1 reduces HIV-1 transcript levels independently of its P-body localization or association with decapping components, implying the involvement of other host factors in regulating viral mRNAs. Similarly, for N4PB1-dependend negative regulation of endogenous transcripts in HaCaT keratinocytes, EDC4 is not essential. For both, HIV-1 and endogenous transcripts, the reduction is associated with the presence of the PIN domain.

Answers to Reviewers' comments:

Reviewer #1 (Remarks to the Author):

Major Point 1

The experiments with RNase A treatment are difficult to interpret. Over time, N4BP1 pulls down less EDC4. This effect is stronger in the presence of RNase A. One explanation is that the interaction is indeed mediated by RNA.

However, the experiment with Benzonase does not show the same result. How do the authors know that Benzonase was working under these conditions?

The discrepancy between the RNase A and Benzonase experiments could be due to the following reasons. Benzonase needs Mg^{2+} as an essential cofactor for its activity. The optimal range is about 1 to 2 mM $MgCl_2$. The Lysis and IP buffer used here does not contain any Mg^{2+}. Instead, it contains 0.5 mM EDTA. EDTA chelates divalent cations and most likely blocks the activity of Benzonase. RNase A is a metal-independent enzyme, so it is not affected by this.

For these reasons, the experiments do not allow a clear conclusion. They need to be repeated.

The section title "RNA may not be necessary for stabilizing the interaction between N4BP1 and EDC4" is not a good choice. It is so vague that readers do not really know what to take away from it.

We would like to sincerely thank the reviewer for this valuable suggestion. Increasing the Mg^{2+} ion concentration, removing EDTA from the buffer, and lowering the reaction temperature during the experiment allowed us to obtain consistent and reproducible results. Accordingly, the title and the subsection "RNA may not be necessary for stabilizing the interaction between N4BP1 and EDC4" were changed in the current version of the manuscript as well. **Fig. 5** is now showing the association between N4BP1 and EDC4 that persists upon RNase A and benzonase treatment at given conditions. Additionally, IP-MS results upon benzonase treatment have been moved to Supplementary Information file (**Fig. S8**).

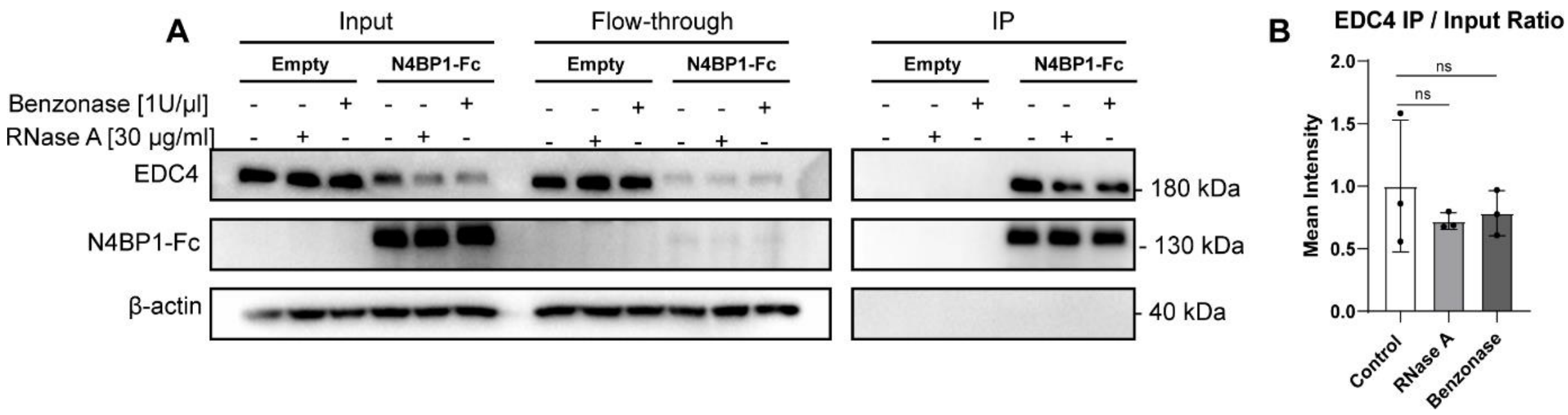

Figure 1 (Figure 5 in the manuscript). The interaction between N4BP1 and EDC4 is preserved upon RNase A or benzonase treatment. A. Co-IP of N4BP1-Fc with EDC4 following RNase A or benzonase treatment for the indicated time points 2h in HEK293T cells. **B.** Densitometric quantification of Western blot signals from panel A, expressed as the EDC4 IP/Input ratio. Data represent mean ± SD from 3 independent experiments. Statistical analysis was performed using Brown-Forsythe and Welch's ANOVA: Control vs RNase A $t(2{,}06) = 0{,}91$, $P = 0{,}66$, Control vs Benzonase $t(2{,}46) = 0{,}67$, $P = 0{,}78$.

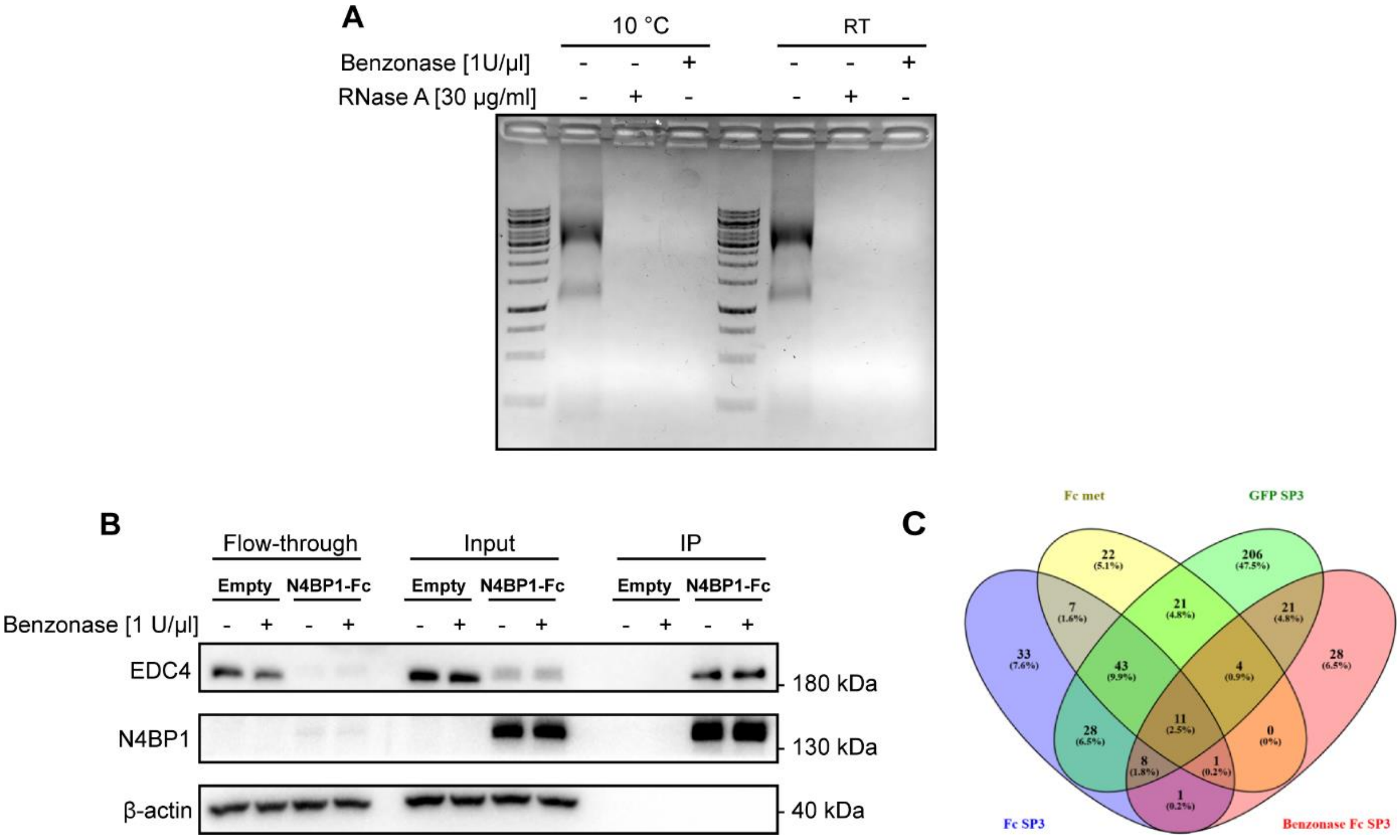

Figure 2 (Figure S8 in Supplementary Information). A. Electrophoresis of 2 µg RNA samples untreated or treated with RNase A or benzonase at 10 °C or room tempeature. All samples were supplemented with 2 mM $MgCl_2$ in order to ensure optimal conditions for benzonase activity. **B.** Western blot analysis of samples subjected to immunoprecipitation following benzonase treatment for 2h at 4 °C. **C.** Venn diagram illustrating number of proteins identified by mass spectrometry under all tested conditions including benzonase treated samples.

Major Point 2
These experiments clearly suggest that the KH domains 1 and 2 are required for the binding to EDC4. So this is done, great.

Thank you!

Points 3 and 4, AlphaFold
The authors mention that some models converge, but they do not mention how many. This information is only partly helpful. The authors should show all five models, or all five PAE plots. Then the reader can judge the data and does not need to run the prediction again.

In our AF2 multi analysis, 3 out of 5 models converged to a similar prediction. We have now mentioned that and included a supplementary figure with all 5 models (**Fig. S12**).

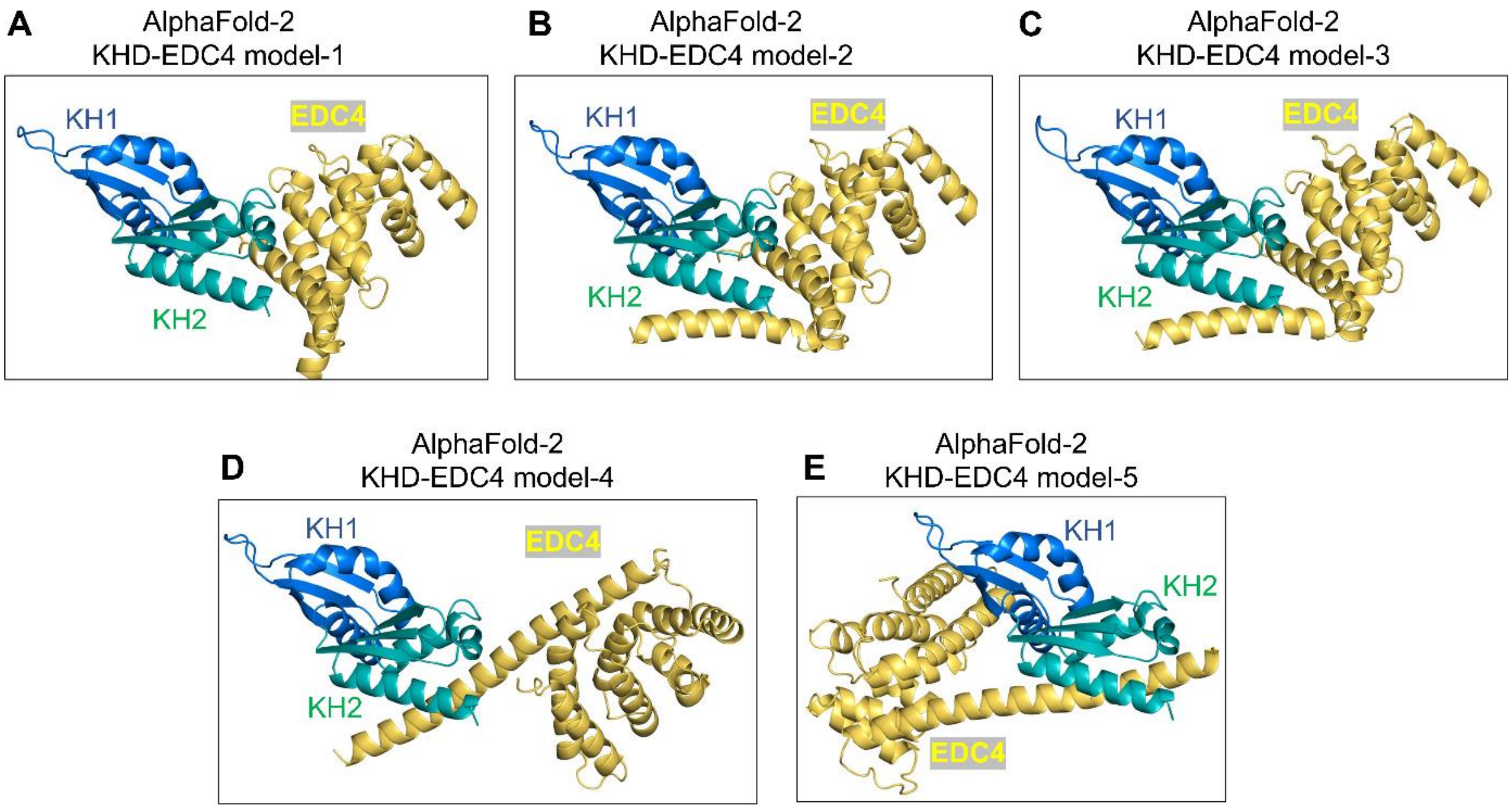

Figure 3 (Figure S12 in Supplementary Information). A-E. Cartoon representation of all 5 structure models for N4BP1 KHD and EDC4 C-fold as predicted by AlphaFold 2.

Was AF2 or AF3 used here? The plots look more like AF3.

AF2 Multi was used to predict the structure models.

The authors also misunderstood what I asked for. The Walter lab offers several resources. First, they predicted a large number of protein-protein interactions. One can browse or screen these to see if a favorite prediction has already been reported. Second, they offer a validation server. This server calculates a Q-score based on the AF2 prediction. The Q-score is a better metric than the iPTM.

The predictome server does not have EDC4 listed as interactor of N4BP1 protein. As suggested, we calculated the Q-score for the prediction, which comes out to be 0.28, suggesting a low confidence model. We have tried to avoid strong claims from the prediction and have mentioned it in the manuscript for clarification as well.

Another way to get a better measure than the iPTM is to use the LIVIA Prediction Analysis. It analyses AF3 predictions directly and provides the LIS score.
For the iPTM, no clear cutoff has been defined. As a rough rule, the IPTM should be above 0.5 for an interaction. The authors observe an iPTM of 0.25 for their best model. This suggests that there is no interaction, or that the predicted model is of very low confidence.
The authors should therefore use one of the two servers to cross-validate their results. In the current form, the AlphaFold analysis is not of sufficient quality to draw a conclusion. The LIS score analyses the five models individually and together. This allows a more robust judgement. If the prediction confidence remains this low, it must be clearly stated in the text so readers are aware of it.

The LIVIA analysis also predicts a low confidence in the KHD-EDC4 interaction, but we have not mentioned that analysis in the manuscript.

Point 5, RNA binding
The strongest point of the manuscript is the structure of the KH1-2 domains. The authors show that this domain tandem also mediates the binding to EDC4.
The authors determined the crystal structure of KH1-2. So they already established the production of the recombinant protein and it should be available.
I did not suggest an extensive systematic RNA binding analysis. I only suggested to test whether the protein can bind RNA at all. This would be very interesting, also with respect to Major Point 1, which still remains unclear. But I will no insist on this point and leaves this up to the editor to decide whether this is an important point or not.

We are currently working on understanding the kinetics of the degradation of endogenous and exogenous mRNA, as well as the RNA-binding sites of N4BP1. The results of these studies will be presented in a subsequent manuscript. We agree that this is very important issue.

Reviewer #2 (Remarks to the Author):

I think the revised manuscript is substantially improved. The revision has addressed most of my previous concerns. In particular, the new domain-mapping data and the benzonase-treated IP-MS experiment help clarify the relationship between N4BP1, EDC4, and other decapping factors. The added RNA-seq and qRT-PCR analyses also make the functional part of the study more complete.

The revised data now support a more nuanced model than the original manuscript. The tandem KH domains appear to mediate the association of N4BP1 with EDC4 and P-body-associated decapping factors, whereas the reduction of HIV-1 transcripts and selected endogenous transcripts is mainly linked to the NYN domain. This distinction is important and should remain clear throughout the manuscript.

1. I suggest softening the wording that describes N4BP1 as a component of the mRNA decapping complex. The data show association and colocalization with decapping factors, but they do not establish that N4BP1 is a core or functional component of the decapping machinery. For example, the section title "N4BP1 RNase is a component of mRNA-decapping complex" could be revised to something more neutral, such as "N4BP1 associates with mRNA-decapping factors."

Indeed, "interaction" is not the most appropriate term to describe the presence of N4BP1 within the decapping protein complex. In accordance with the reviewer's suggestion, we use the term "interaction" when referring specifically to EDC4, whereas we use "association" when referring to the decapping complex.

2. The endogenous transcript analysis should also be described with some caution. Because the RNA-seq experiment is based on ectopic N4BP1 expression, and because transcript half-life or direct RNA binding was not tested, FREM2, ETV1, and NOTCH3 are better described as N4BP1-sensitive transcripts rather than direct endogenous targets. Along similar lines, "transcript reduction" or "N4BP1-mediated repression" may be more appropriate than "degradation" in places where RNA stability was not directly measured.

In this case as well, the suggested changes have been implemented, and we now use the terms proposed by the reviewer.

3. The RNase A and benzonase results should be interpreted carefully. The RNase A experiment suggests that RNA may contribute to the N4BP1–EDC4 association, whereas the benzonase experiment indicates that at least part of the N4BP1-associated decapping-factor complex is resistant to nuclease treatment. These results are useful, but they do not by themselves establish a direct binary interaction between N4BP1 and EDC4. The text should therefore continue to distinguish between direct binding and association within a larger protein or RNP complex.

The RNase A and Benzonase experiment was repeated in accordance with the suggestion of one of the reviewers, who recommended increasing the Mg^{2+} ion concentration and removing EDTA. At this point, **Fig. 5** presents the updated result, which allows for a fully consistent conclusion: RNA does not participate in the formation of the N4BP1 complex with EDC4 and the core decapping proteins.

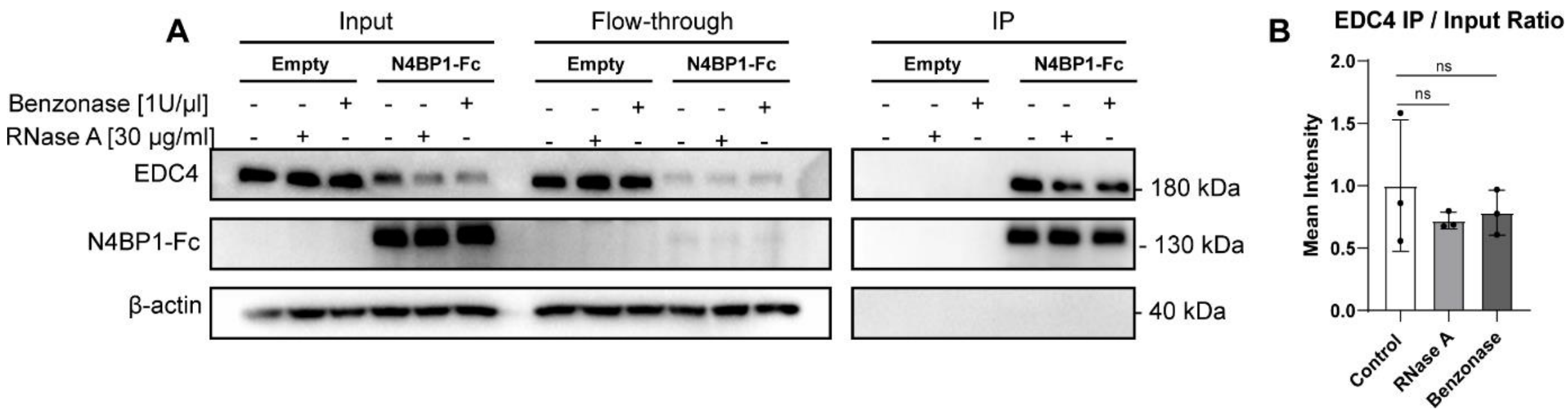

Figure 1 (Figure 5 in the manuscript). The interaction between N4BP1 and EDC4 is preserved upon RNase A or benzonase treatment. A. Co-IP of N4BP1-Fc with EDC4 following RNase A or benzonase treatment for the indicated time points 2h in HEK293T cells. **B.** Densitometric quantification of Western blot signals from panel A, expressed as the EDC4 IP/Input ratio. Data represent mean ± SD from 3 independent experiments. Statistical analysis was performed using Brown-Forsythe and Welch's ANOVA: Control vs RNase A $t(2{,}06) = 0{,}91$, $P = 0{,}66$, Control vs Benzonase $t(2{,}46) = 0{,}67$, $P = 0{,}78$.

Answer to Reviewers comment:

Reviewer #1 (Remarks to the Author):
The Authors have repeated the experiment with Benzonase. Now the picture is more clear.
What is unclear to me: Was the IP mass spec experiment with Benzonase repeated in presence of Mg2+ ions or is the experiment, which was moved to the supplment (Suppl. Figure 8) the old one? The Materials and Methods section would, to me, suggest that IP-MS is the same, but the experiment for Figure 5 is new.
If so, the caveat should be clearly stated.
The discussion section AF is good now; it clearly shows the limitations of the prediction for the readers.
So overall I am ok.

We thank the Reviewer for this helpful comment. We indeed confirm that the IP–MS experiment with Benzonase shown in Supplementary Figure 8 corresponds to the original experiment (i.e. the old one). In this experiment, we did not add EDTA to the IP buffer, and relied on endogenous Mg^{2+} present in the lysate to support Benzonase activity. However, because the exact free Mg^{2+} concentration was not controlled in this setting, we consider this experiment less rigorous and have therefore moved it to the Supplementary Information. In contrast, the experiment shown in **Figure 5** is new and was performed with Mg^{2+} ions deliberately added to the IP buffer, ensuring optimal Benzonase activity under defined conditions.

We have now clarified this point in the Materials and Methods section as stated:

RNase A / Benzonase treatment: HEK293T cells were seeded at the density of 400,000 cells per well on 6-well plates. After 24 h cells were transfected with N4BP1-Fc pcDNA3.1 or an empty vector using JetPRIME reagent. Cells were lysed as described above (see Methods: Immunoprecipitation). After diluting the lysate with dilution buffer, RNase A (EURx) or Benzonase (Millipore) was added to a final concentration of 30 µg/ml or 1 U/µl respectively. Samples were incubated at 10 °C or RT for 2 h. Afterwards, the samples were processed according to the immunoprecipitation protocol using EDTA-free buffers supplemented with 2 mM Mg^{2+} ions instead of the standard EDTA-containing buffers. In the IP-MS experiment (**Supplementary Figure 8**) Mg^{2+} ions were not supplemented and Benzonase activity therefore relied on endogenous divalent cations present in the lysate.
