# Supplementary material for: N4BP1 uses tandem KH domains to associate with EDC4 and mRNA decapping factors in P-bodies
Source: Commun Biol. 2026 Sep 17;9:1215. doi: 10.1038/s42003-026-10938-x (PMC13586276; doi:10.1038/s42003-026-10938-x)
Supplement: Supplementary file 2 [file 42003_2026_10938_MOESM2_ESM.pdf]

SUPPLEMENTARY INFORMATION

N4BP1 uses tandem KH domains to associate with EDC4 and mRNA decapping factors in P-bodies

Paweł Piłat[1,2,#], Ankur Garg[3,4,5,6,#], Udo Heinemann[3,4,*], Mateusz Wilamowski[1*], Jolanta Jura[1*]

[1] Jagiellonian University, Faculty of Biochemistry, Biophysics and Biotechnology, Department of General Biochemistry, Gronostajowa 7, 30-387 Krakow, Poland

[2] Jagiellonian University, Doctoral School of Exact and Natural Sciences, Lojasiewicza 11, 30-348 Krakow, Poland

[3] Macromolecular Structure and Interaction, Max-Delbrück Center for Molecular Medicine in the Helmholtz Association, Robert-Rössle-Str. 10, 13125 Berlin, Germany

[4] Institute for Chemistry and Biochemistry, Freie Universität Berlin, Takustr. 6, 14195 Berlin, Germany

[5] Cold Spring Harbor Laboratory, 1 Bungtown Road, Cold Spring Harbor, New York 11724, USA

[6] Howard Hughes Medical Institute, Cold Spring Harbor Laboratory, 1 Bungtown Road, Cold Spring Harbor, New York 11724, USA

[5, 6] Current affiliations

[#] Joint first authors

***Corresponding authors:**
Jolanta Jura and Mateusz Wilamowski
Faculty of Biochemistry, Biophysics and Biotechnology, Jagiellonian University,
Gronostajowa 7, 30-387 Kraków, Poland
e-mail: jolanta.jura@uj.edu.pl; mateusz.wilamowski@uj.edu.pl
Udo Heinemann
Macromolecular Structure and Interaction, Max Delbrück Center for Molecular Medicine in the Helmholtz Association, Robert-Rössle-Str. 10, 13125 Berlin, Germany
e-mail: heinemann@mdc-berlin.de

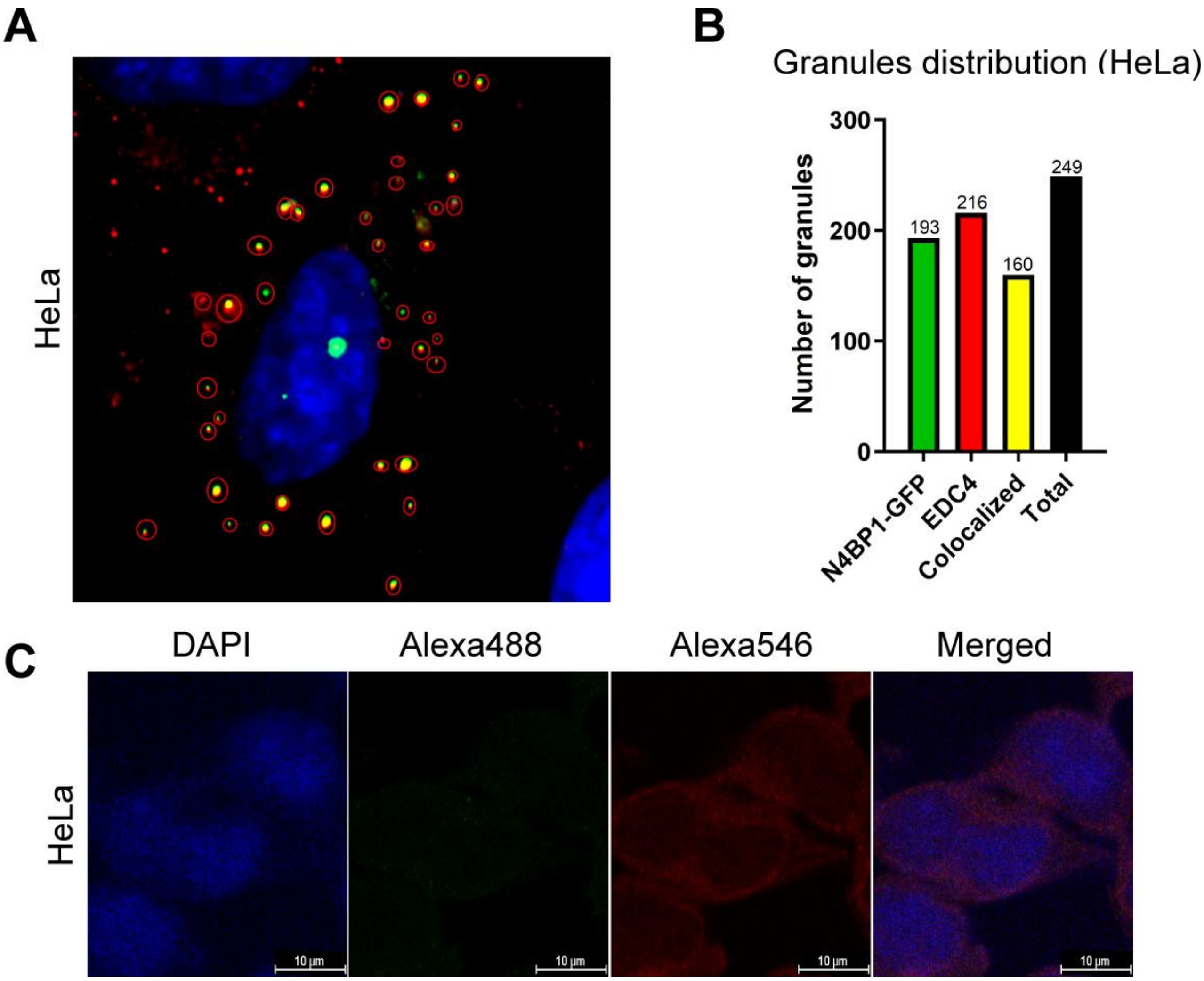

Figure S1. N4BP1-GFP, EDC4 and colocalizing granules. **A.** Circled granules that were counted on each picture. **B.** Granule distribution counted from N4BP1-GFP expressing HeLa cells, sum from 5 pictures for each separate replicate, n=3. **C.** Confocal images of secondary antibodies only control in HeLa cells.

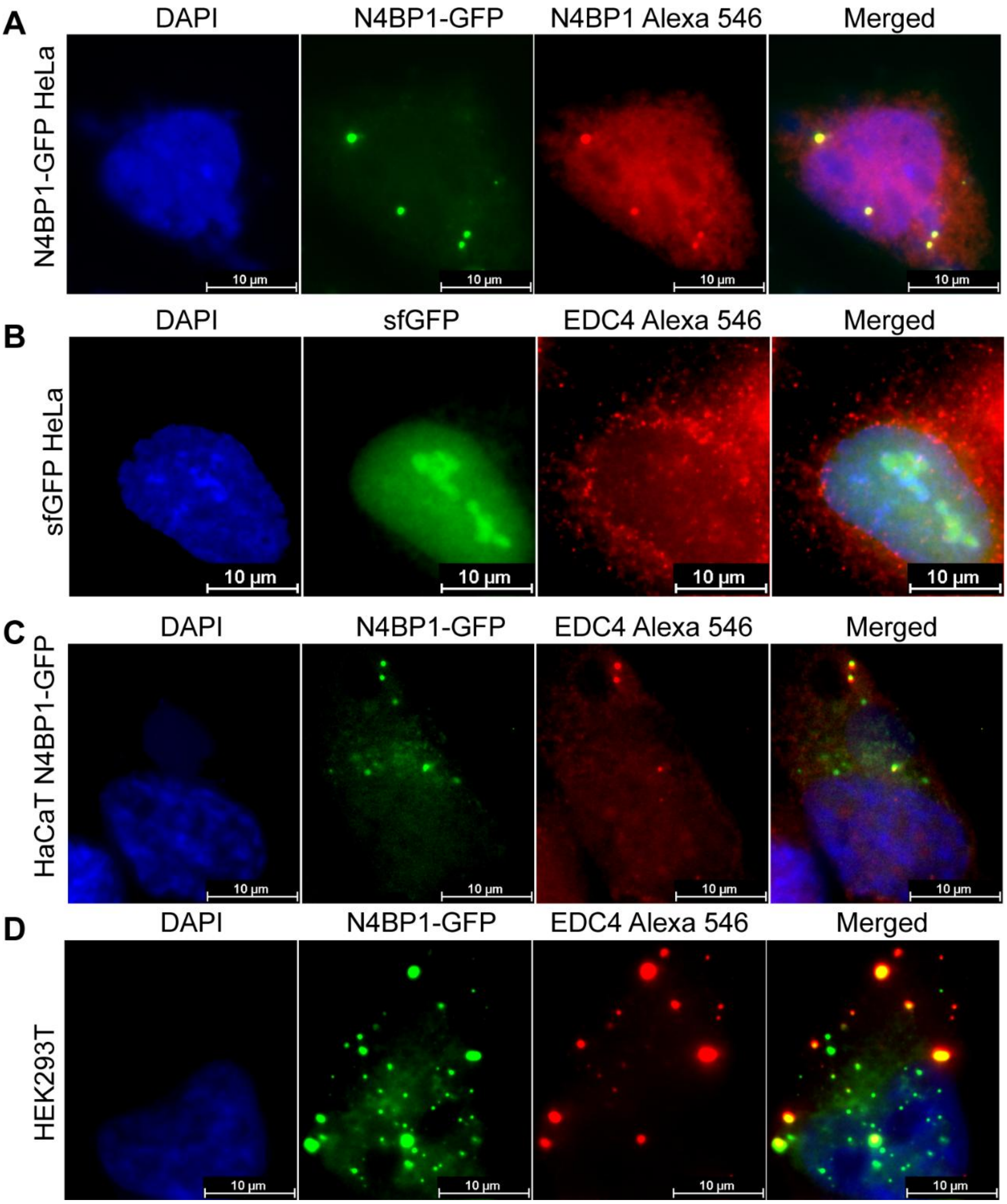

Figure S2. Control of immunofluorescent staining. A. Immunofluorescent staining of N4BP1 with anti-N4BP1 Ab in HeLa cells transfected with N4BP1-GFP pcDNA3.0. **B.** Immunofluorescent staining of EDC4 in HeLa cells transfected with GFP pcDNA3.0. GFP alone does not colocalize with EDC4. **C.** Immunofluorescent staining of EDC4 in HaCaT cells transfected with N4BP1-GFP pcDNA3.0. **D.** Immunofluorescent staining of EDC4 in N4BP1-GFP expressing HEK293T cells.

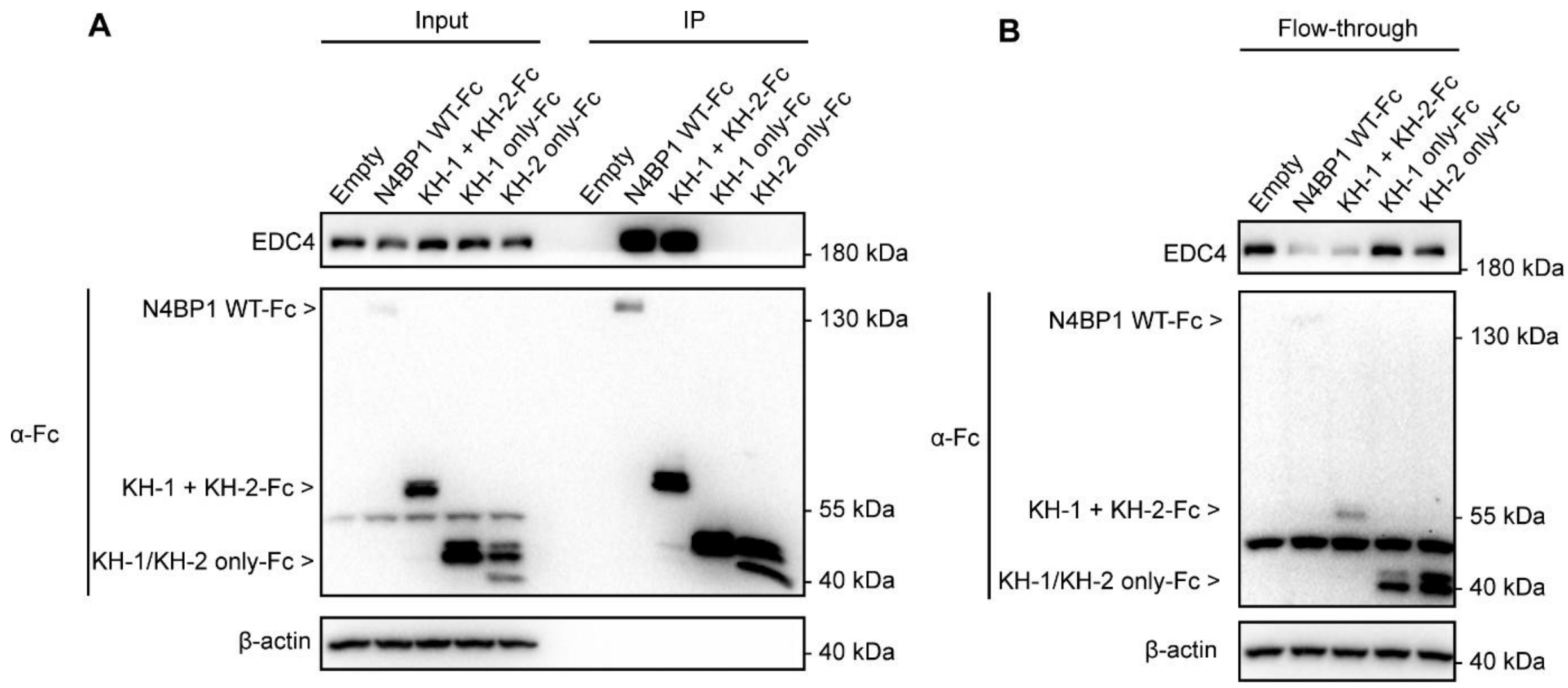

Figure S3. Analysis of the impact of the KH domains of N4BP1 on its association with the decapping complex, represented by EDC4. A. Pull-down assay of full length N4BP1 and its N-terminal fragments (tandem KH domains, KH1 and KH2), followed by Western-blot analysis. **B.** Western blot analysis of flow-through fractions from the pull-down assay of N4BP1s fragments. Presented results are representative of 3 biological replicates.

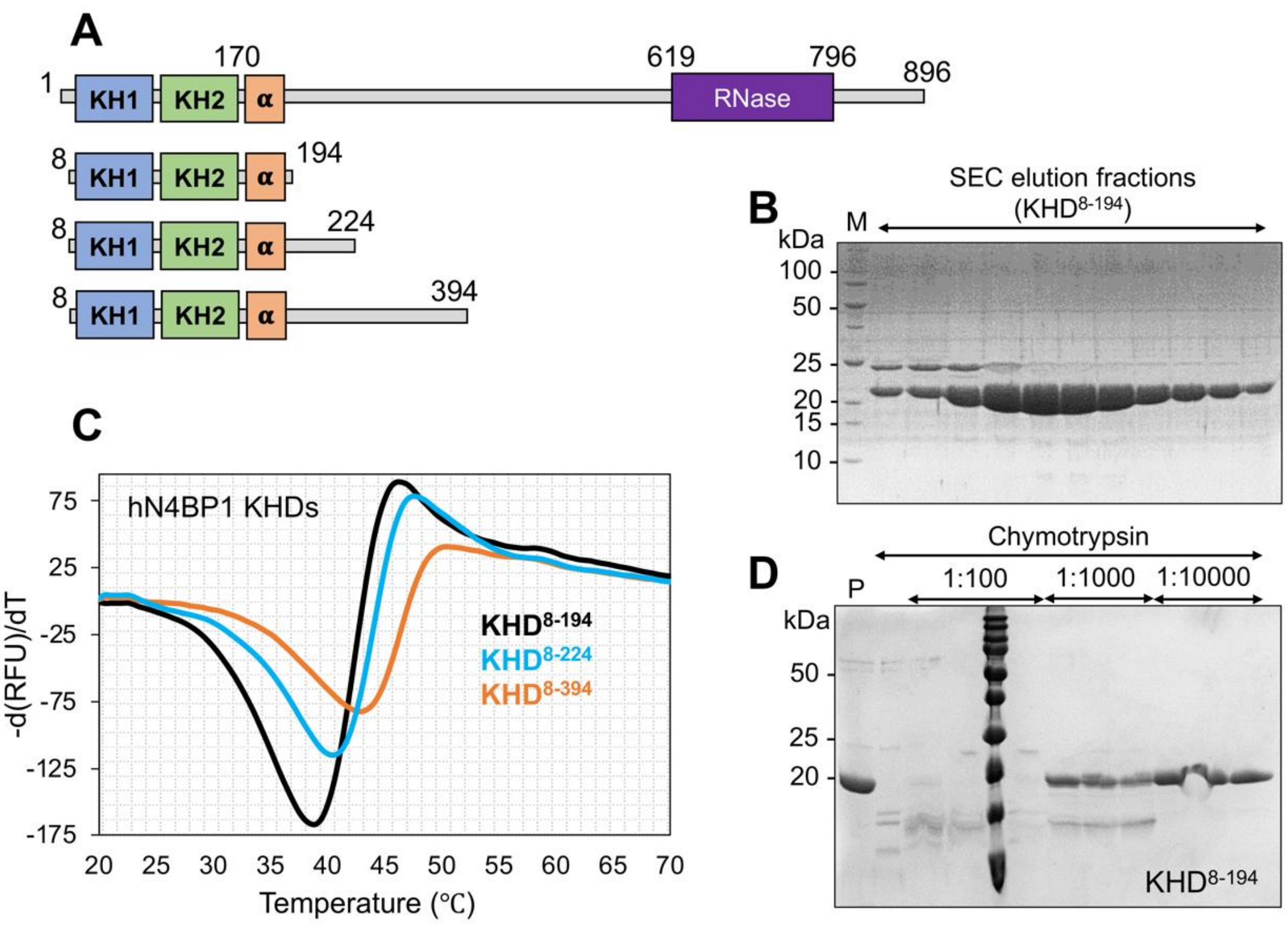

Figure S4. Purification and biophysical characterization of N4BP1 KH domains. A. Domain architecture of the human N4BP1 and different N4BP1 constructs harboring KHDs used in the study. **B.** SDS-PAGE showing size exclusion chromatography (SEC) fractions for N4BP1 KHD$^{8\text{-}194}$ protein. Only the fractions containing pure KHD$^{8\text{-}194}$ protein were further used. **C.** Thermal-melting transitions for different KHD proteins. **D.** Limited proteolysis of KHD$^{8\text{-}194}$ with chymotrypsin. KHD$^{8\text{-}194}$ shows minimal degradation at 1:10,000 w/w ratio and was used for crystallization.

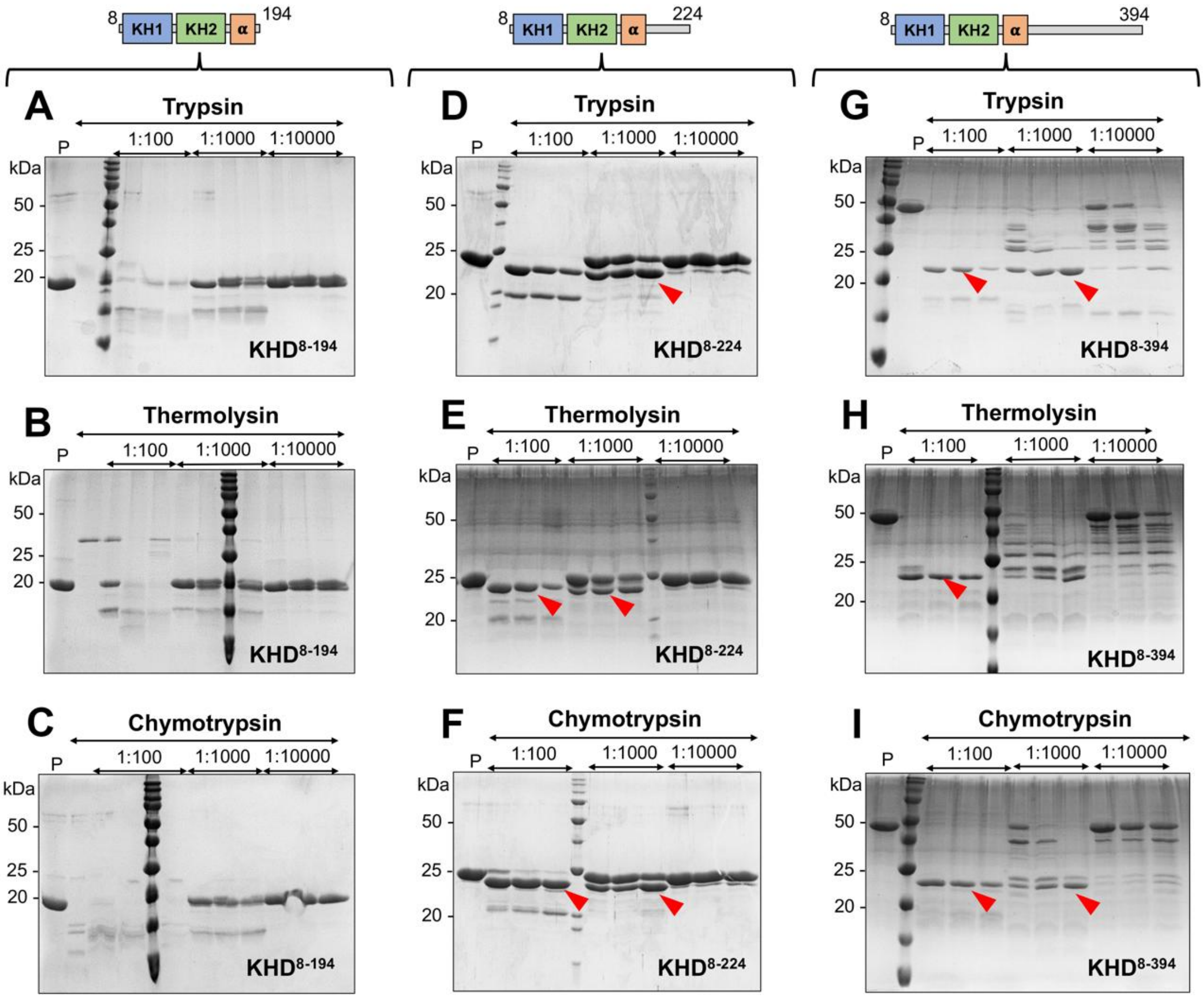

Figure S5. Limited proteolysis of N4BP1 KHDs. Limited proteolysis of KHD$^{8\text{-}194}$ with **A.** trypsin, **B.** thermolysin and **C.** chymotrypsin. Limited proteolysis of KHD$^{8\text{-}224}$ with **D.** trypsin, **E.** thermolysin and **F.** chymotrypsin. Limited proteolysis of KHD$^{8\text{-}394}$ with **G.** trypsin, **H.** thermolysin and **I.** chymotrypsin. KHD$^{8\text{-}194}$ shows only slight degradation with the proteases. The stable products formed after proteolysis of KHD$^{8\text{-}224}$ and KHD$^{8\text{-}394}$ are marked with red arrowheads.

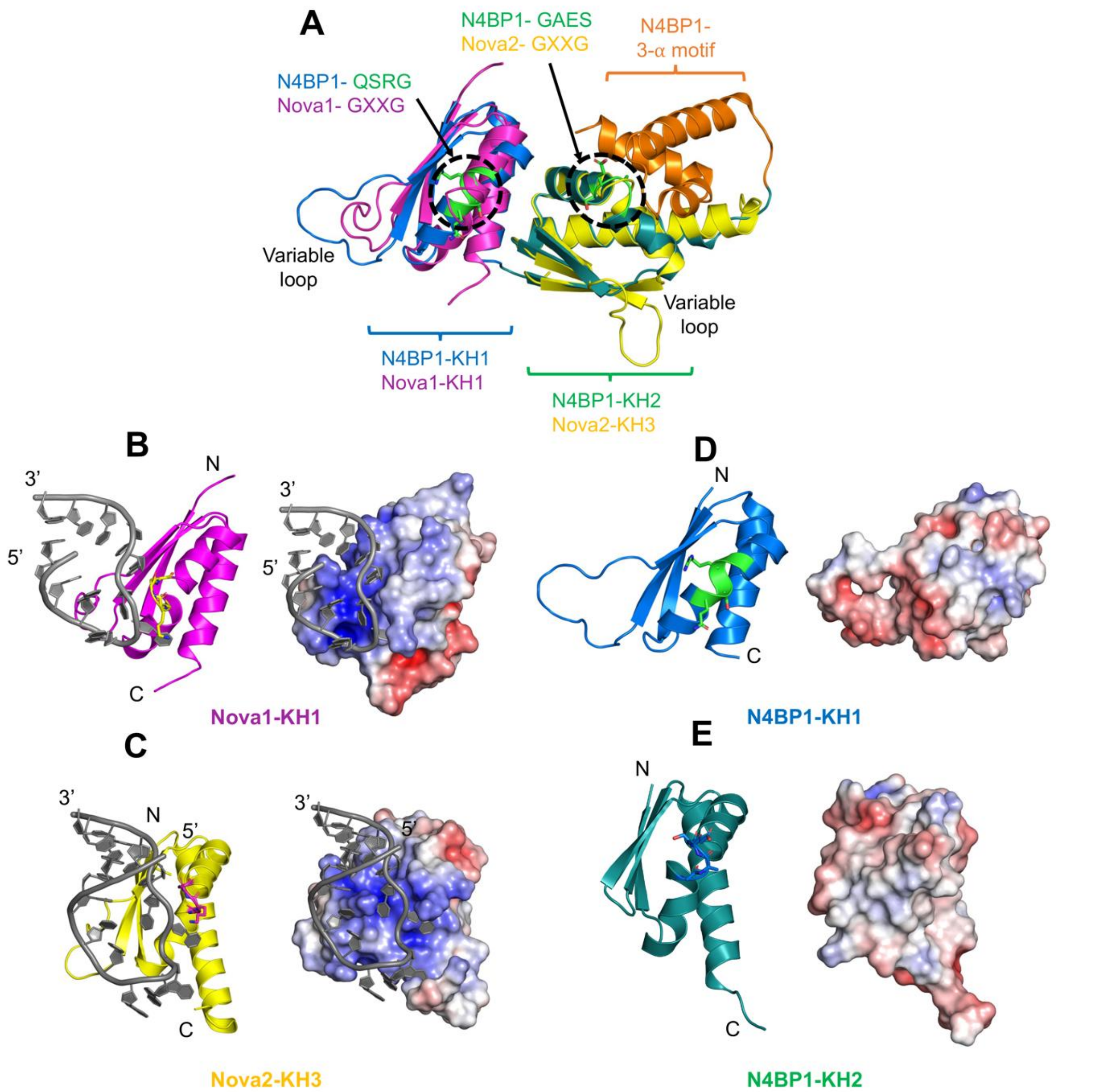

Figure S6. N4BP1 exhibits non-canonical features in its KH domains. A. Structural superposition of N4BP1 KHD$^{8\text{-}194}$ with the Nova1 KH1 (PDBid 2ANN, magenta) and Nova2 KH3 (PDBid 1EC6, yellow) structures. The signature invariable GXXG motif of KH domains is mutated to QSRG (shown as sticks in green) in N4BP1 KH1 and to GAES (shown as sticks in green) in KH2. Both sites are highlighted with black dotted ellipses. Cartoon and electrostatic surface representation of **B.** the Nova1 KH1-RNA structure (PDBid 2ANN) and **C.** the Nova2 KH3-RNA structure (PDBid 1EC6). The GXXG motif (shown as sticks) and the positively charged grooves on Nova1/2 KHDs support the RNA interaction. Cartoon and electrostatic surface view of the N4BP1 KH1 **D.** and KH2 **E.** domains. The N4BP1 KHDs lack a positive groove for RNA binding and their GXXG motifs (shown as sticks) are mutated. The electrostatic surface potential is displayed in a range from –5 (red) to +5 kT/e (blue).

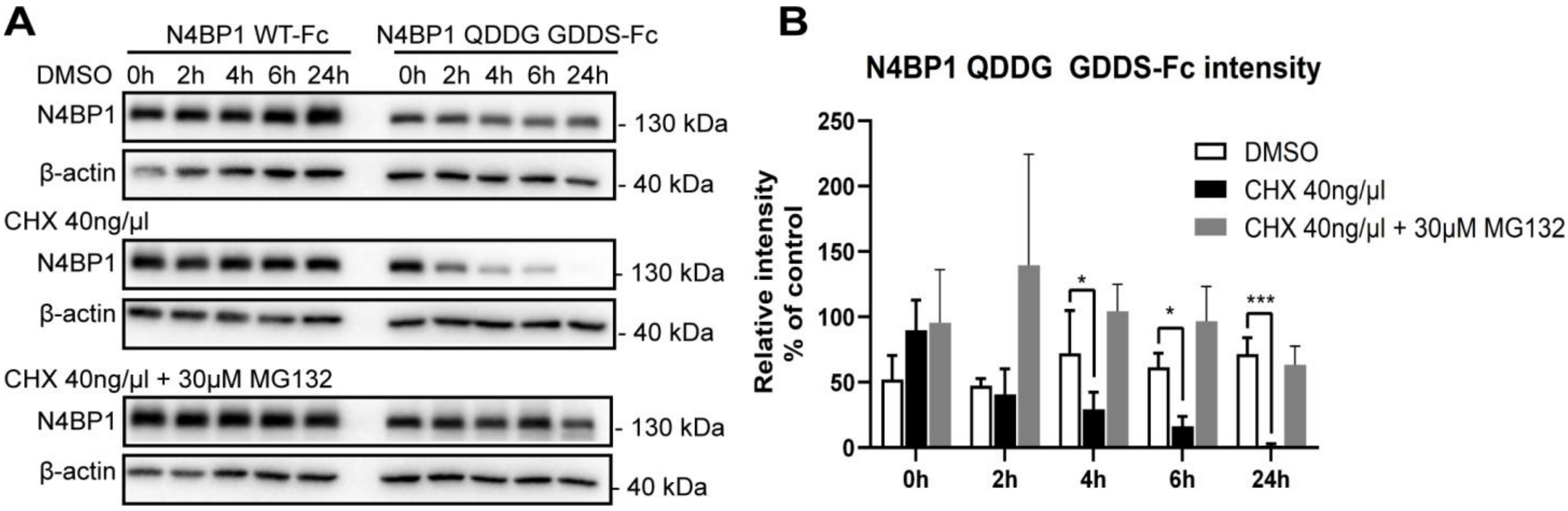

Figure S7. QDDG GDDS N4BP1 mutant undergoes proteasomal degradation. A. Western blot of WT and QDDG GDDS N4BP1-Fc mutant after stimulation with cycloheximide (CHX, 40 ng/µl) and cycloheximide with 30 µM MG132. DMSO was used as a control. **B.** Densitometric analysis of Western Blot shown of Fig. S6A. Values presented are mean ± SD counted as % of control (N4BP1 WT-Fc), relative to β-actin. Two way ANOVA was used for statistical analysis. Data were collected from 3 independent experiments. *p < 0,05, ***p < 0,001.

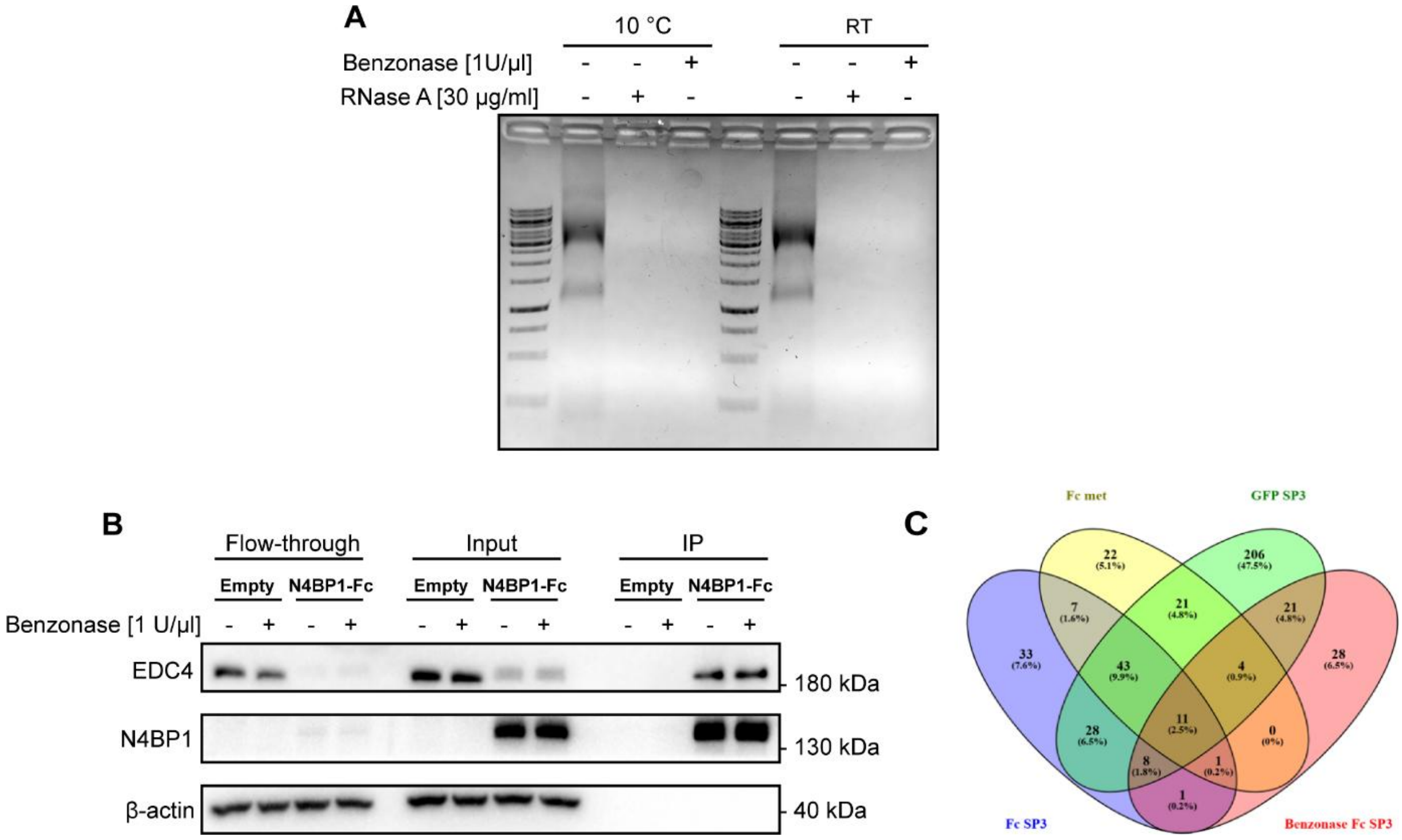

Figure S8. A. Electrophoresis of 2 µg RNA samples untreated or treated with RNase A or benzonase at 10 °C or room tempeature. All samples were supplemented with 2 mM $MgCl_2$ in order to ensure optimal conditions for benzonase activity. **B.** Western blot analysis of samples subjected to immunoprecipitation following benzonase treatment for 2h at 4 °C. **C.** Venn diagram illustrating number of proteins identified by mass spectrometry under all tested conditions including benzonase treated samples.

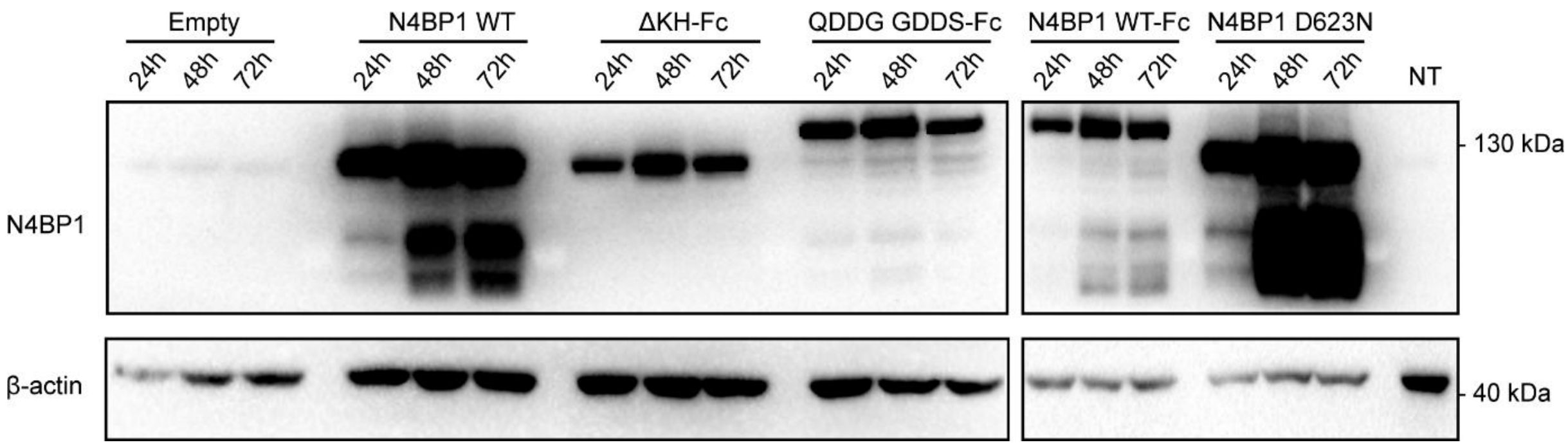

Figure S9. Western blot analysis at 48 hours post-transfection confirming robust expression of wild-type (WT) N4BP1 as well as the D623N, ΔKH-Fc, QDDG GDDS-Fc mutants, along with Fc-tagged WT N4BP1 in HEK293T cells.

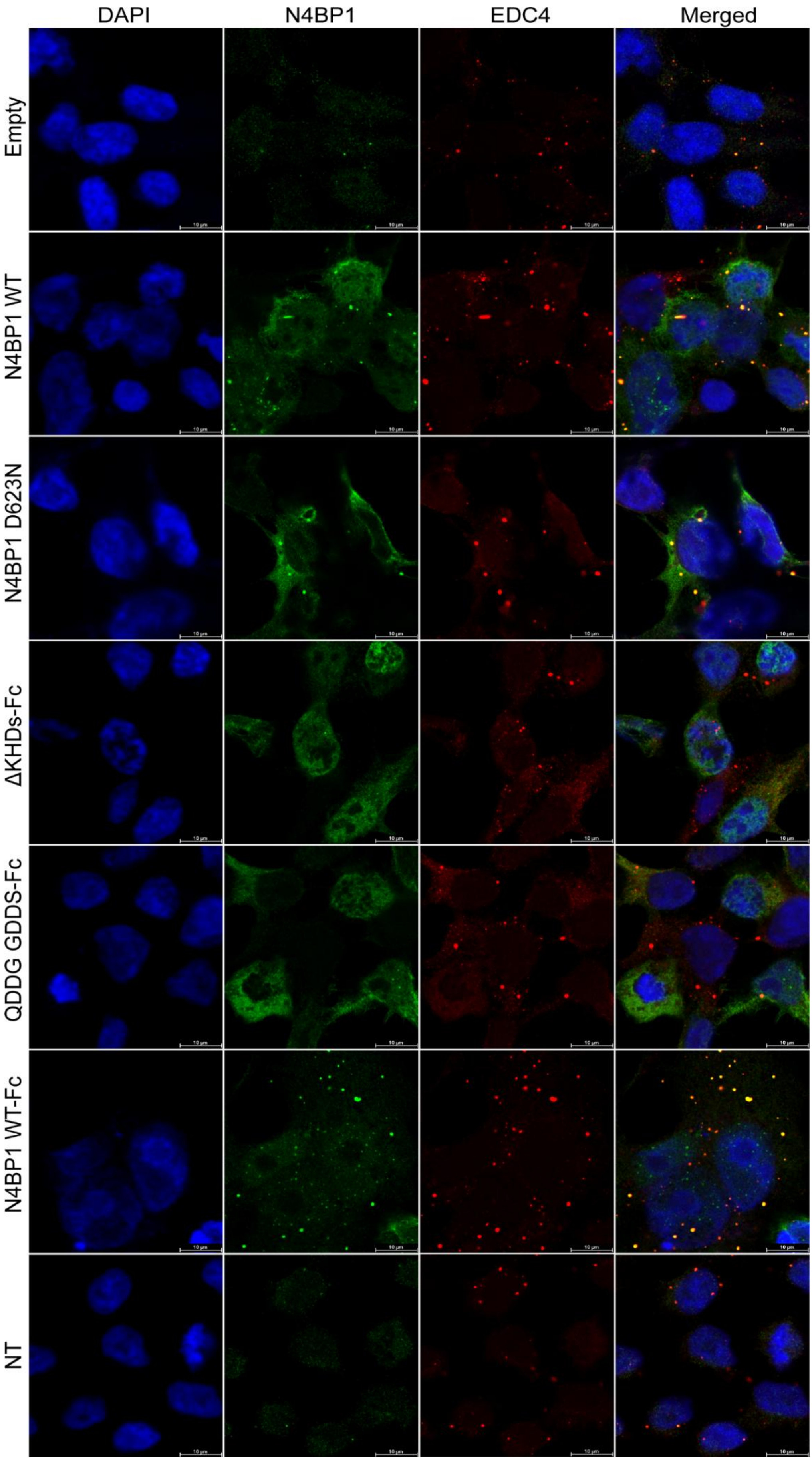
DAPI
N4BP1
EDC4
Merged
Empty
N4BP1 WT
N4BP1 D623N
ΔKHDs-Fc
QDDG GDDS-Fc
N4BP1 WT-Fc
NT

Figure S10. Immunofluorescent staining of N4BP1 and EDC4 in HEK293T cells after co-transfection with empty vector or N4BP1 expression vector and pspax2. Cells were fixed 48 hours post-transfection. In the case of empty and non-transfected (NT) samples endogenous N4BP1 can be observed.

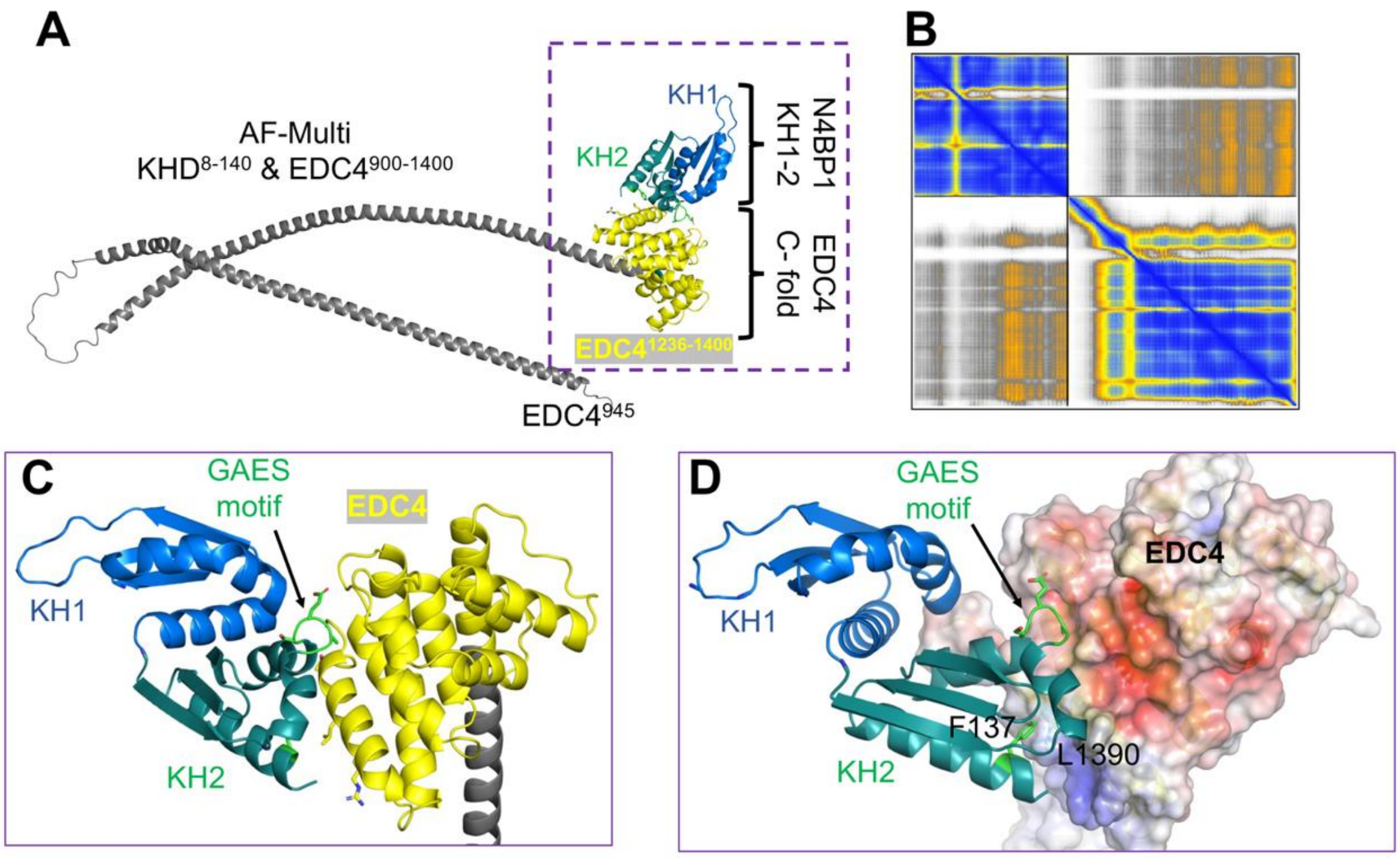

Figure S11. Model of the N4BP1-EDC4 complex generated using AlphaFold-Multi illustrates interaction between tandem KHDs of N4BP1 and the C-terminal α-helical fold of EDC4. **A.** Cartoon representation of modeled structure of EDC4 (depicted as gray and yellow) shows interaction with tandem KHDs of N4BP1 (depicted as blue and teal). **B.** The PAE plot for the predicted structure model. **C.** The cartoon representation of the interface between the N4BP1 KHD and the EDC4 C-terminal fold (C-fold). The KH2 GAES motif (shown in green sticks) seems to directly interact with the EDC4 C-fold. **D.** N4BP1 KH2 GAES motif docks onto the surface of EDC4 C-fold. The KHD is shown in cartoon and the C-fold is shown as electrostatic surface with potential range from -5 (red) to +5 kT/e (blue).

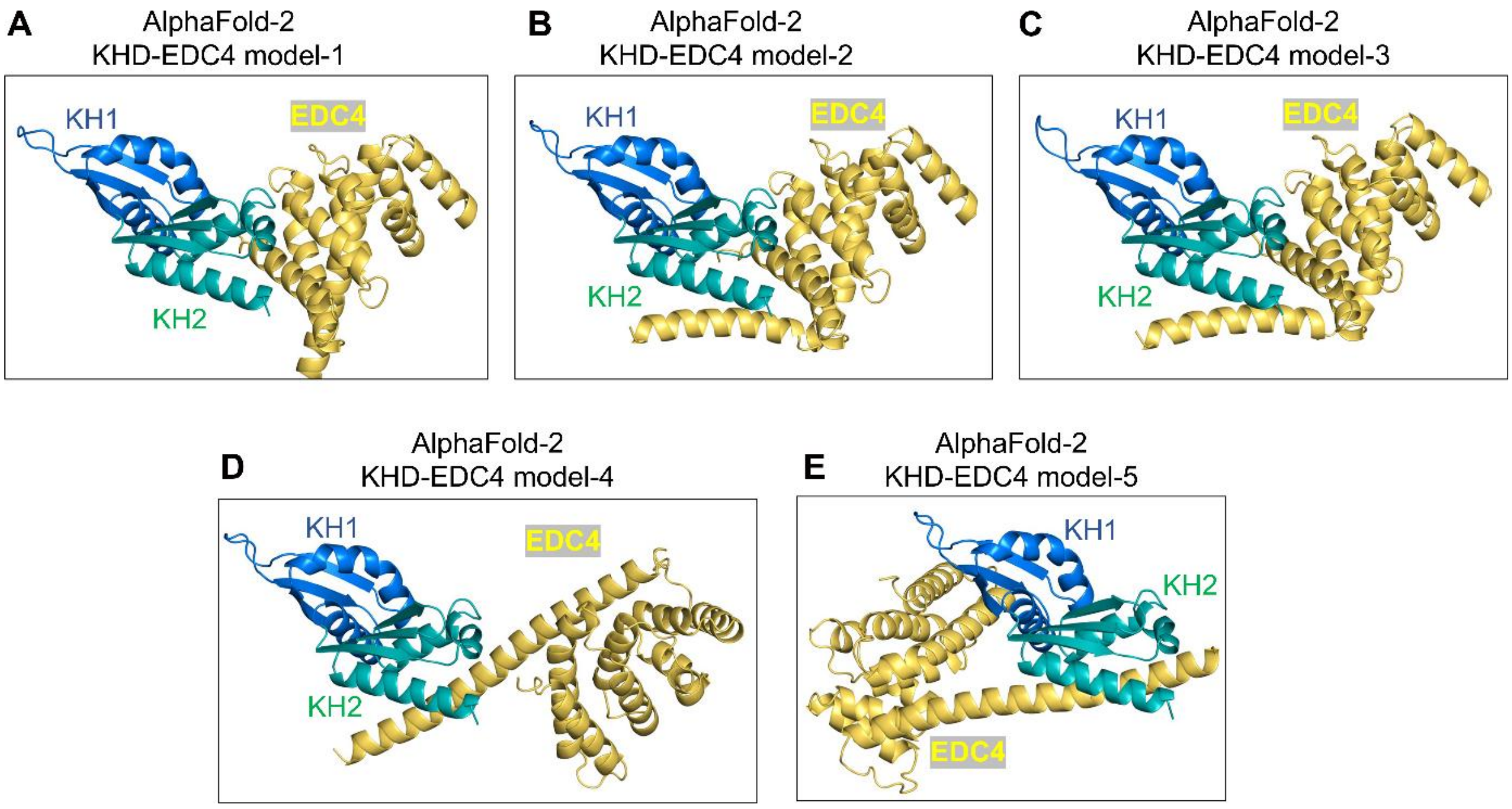

Figure S12. Cartoon representation of all 5 structure models for N4BP1 KHD and EDC4 C-fold as predicted by AlphaFold 2.

Raw blot images (merged with MW marker) for **Fig. 1A, 1B**

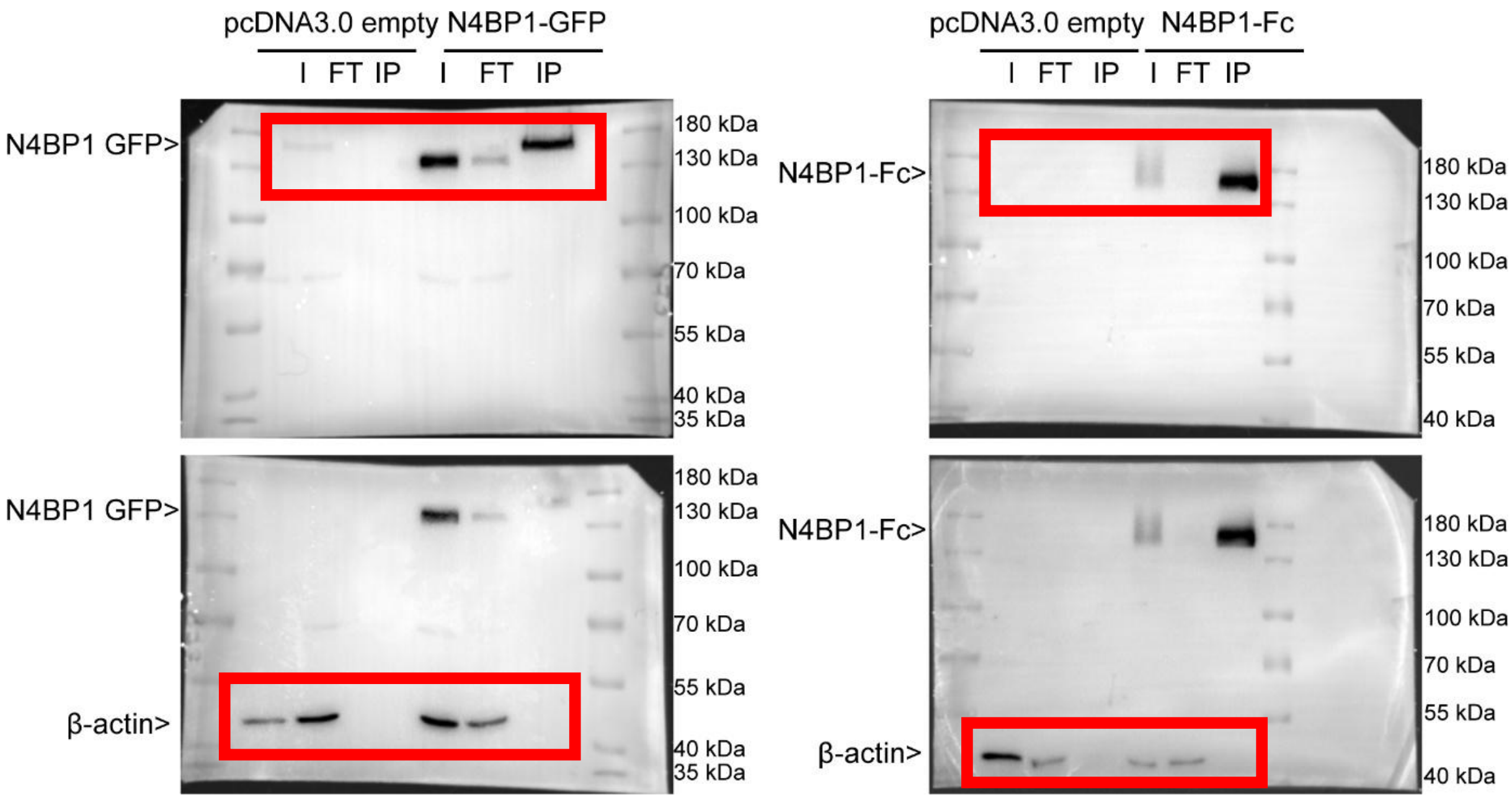

Raw blot images (merged with MW marker) for **Fig. 2F**

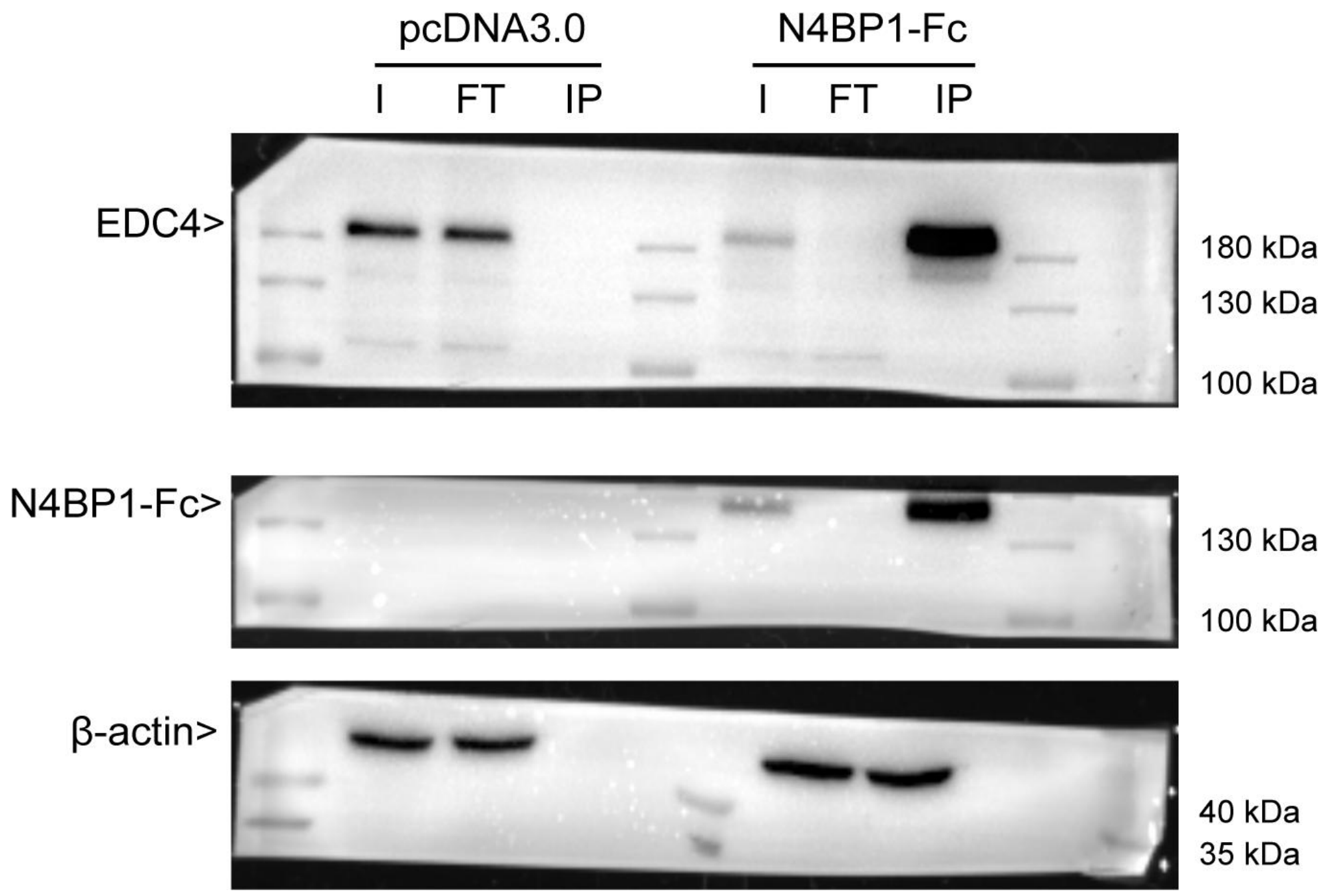

Raw blot images (merged with MW marker) for **Fig. 2G**

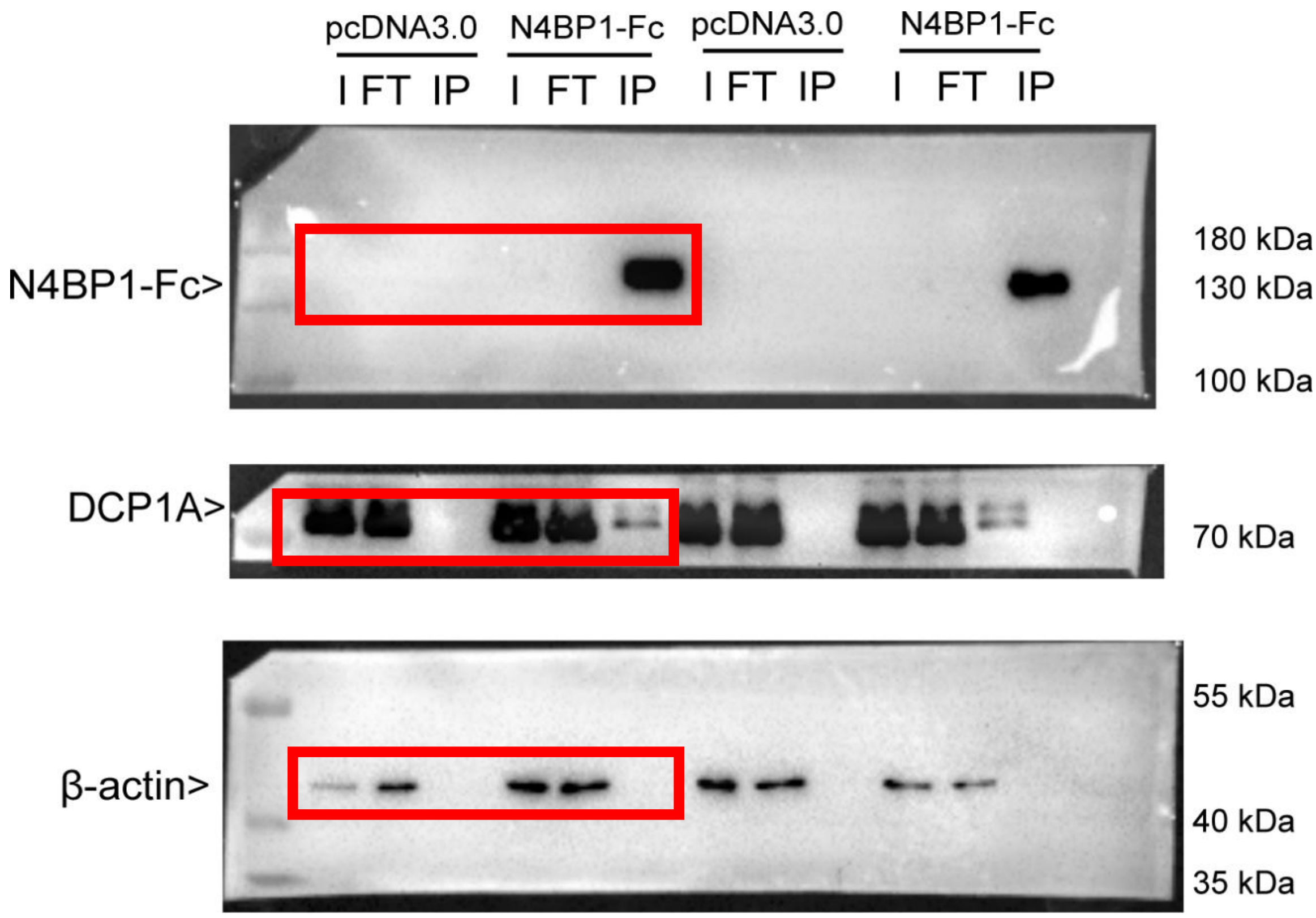

Raw blot images (merged with MW marker) for **Fig. 2H**

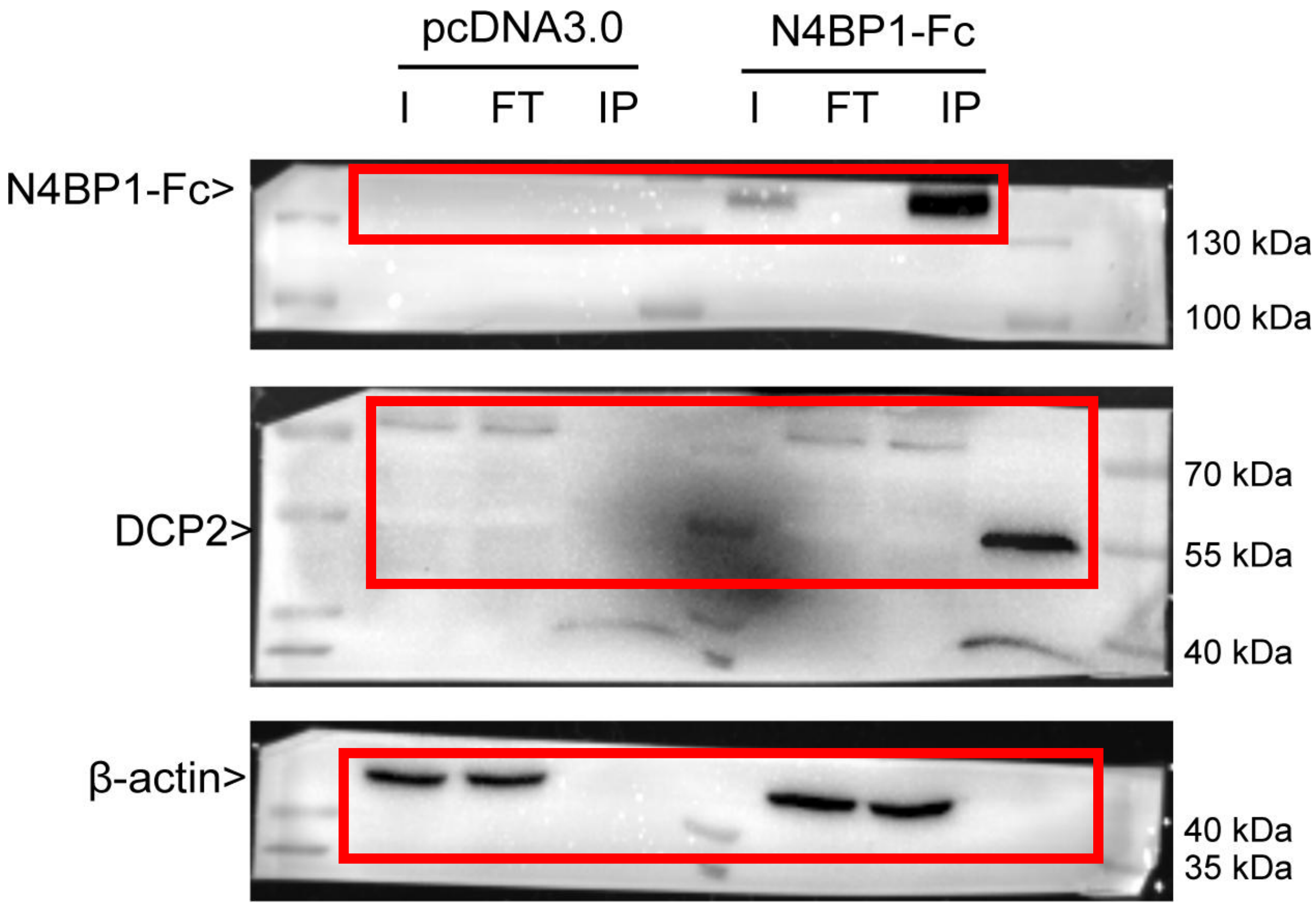

Raw blot images (merged with MW marker) for **Fig. 2I**

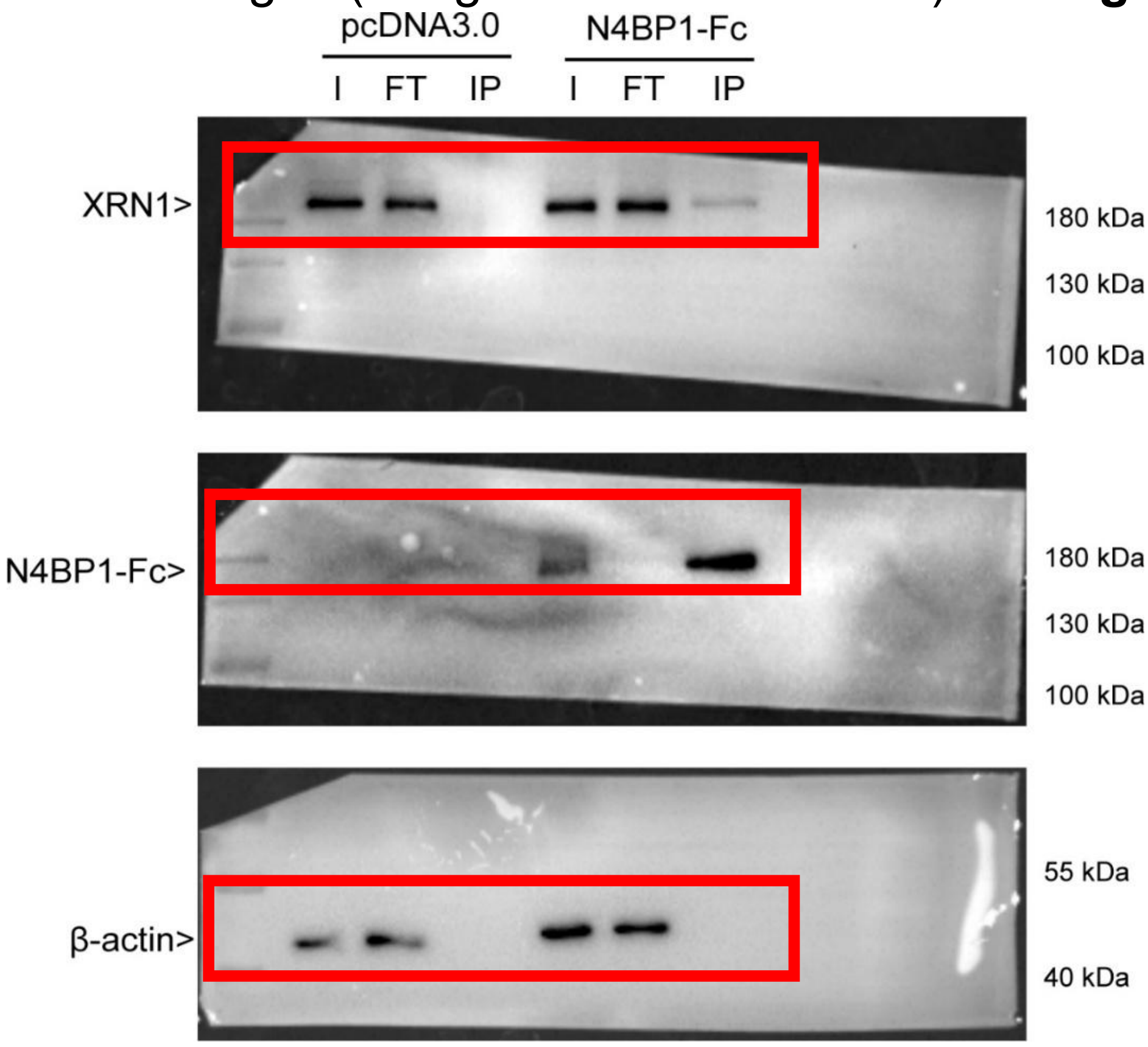

Raw blot images (merged with MW marker) for **Fig. 2K**

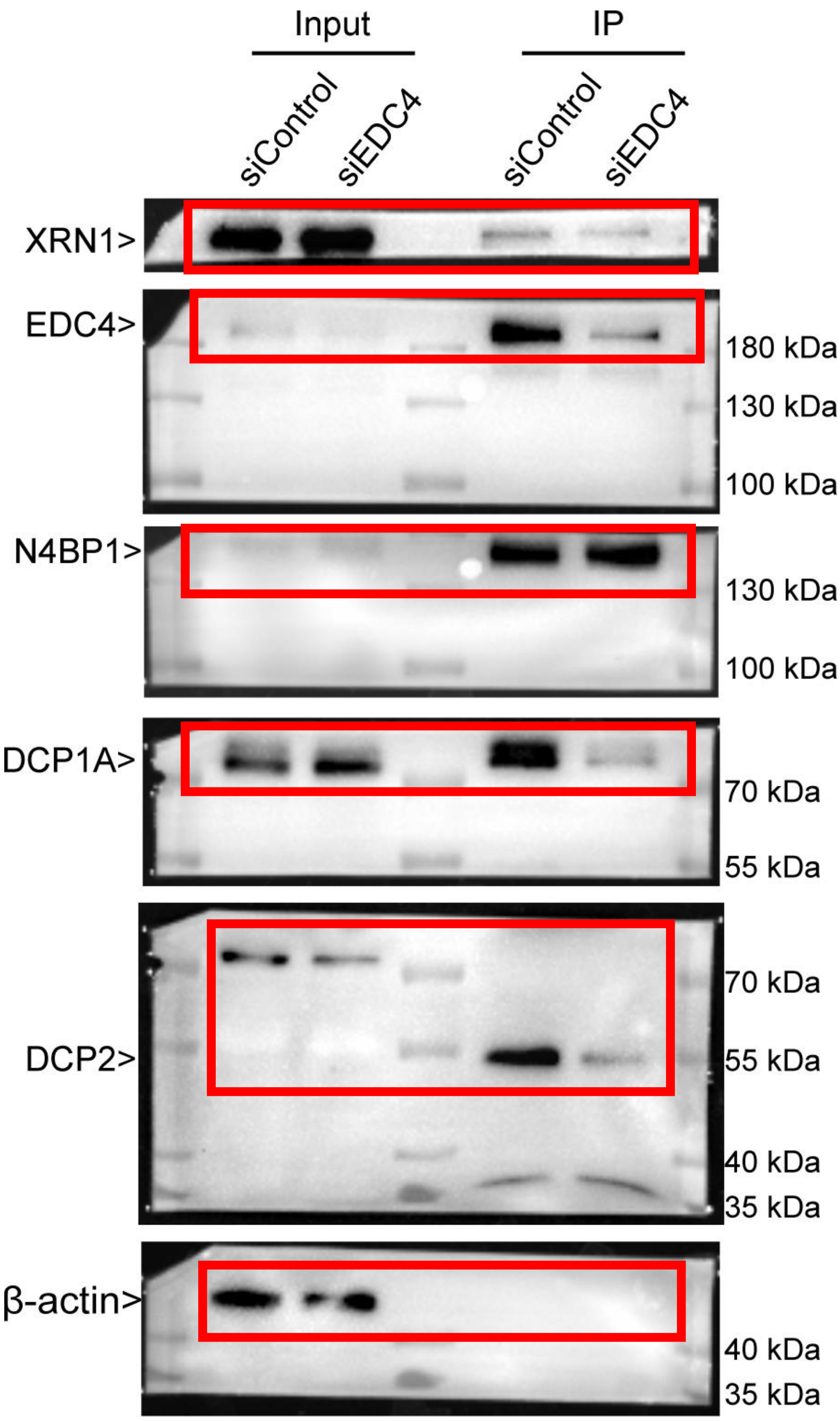

Raw blot images (merged with MW marker) for **Fig. 3B**

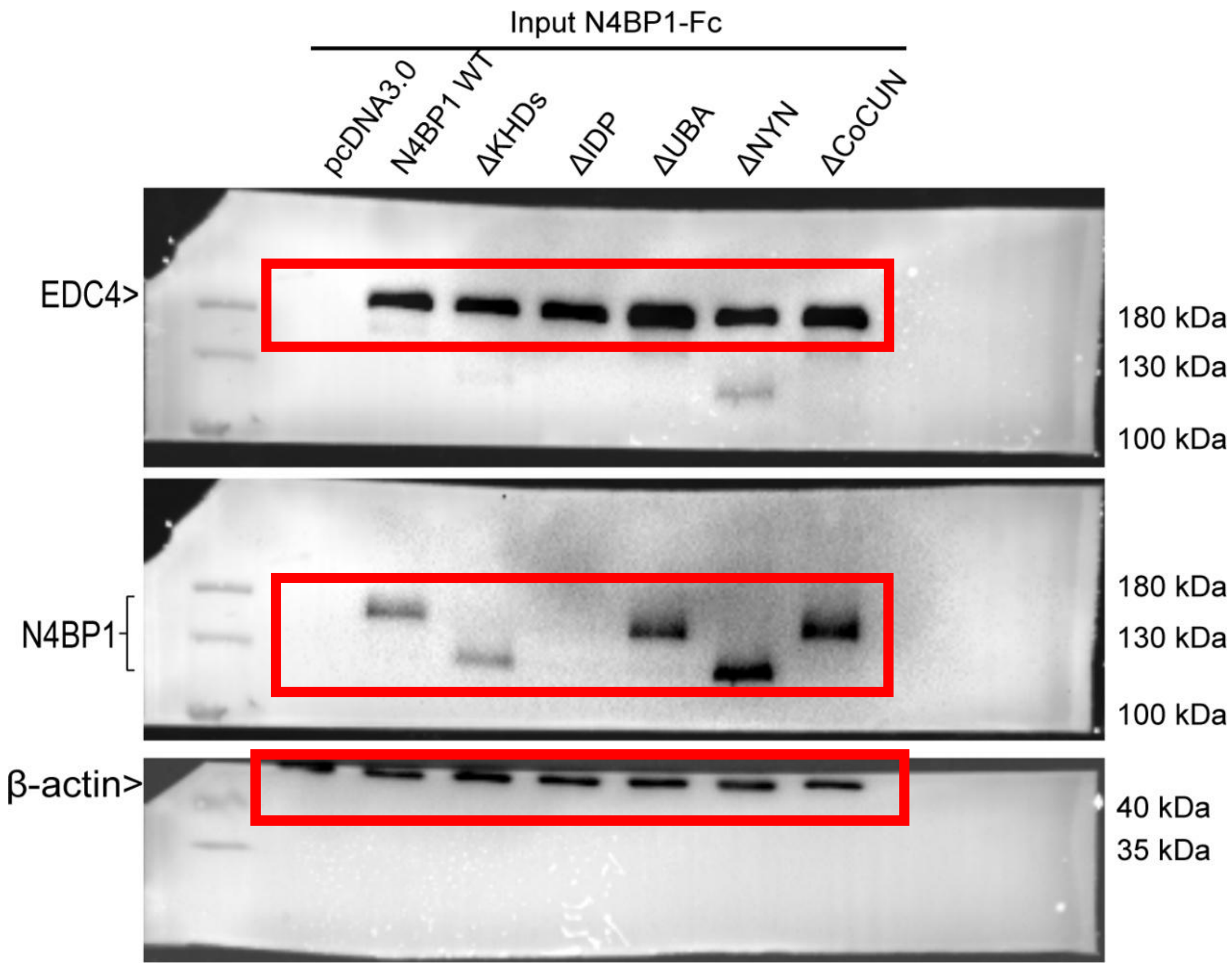

Raw blot images (merged with MW marker) for **Fig. 3C**

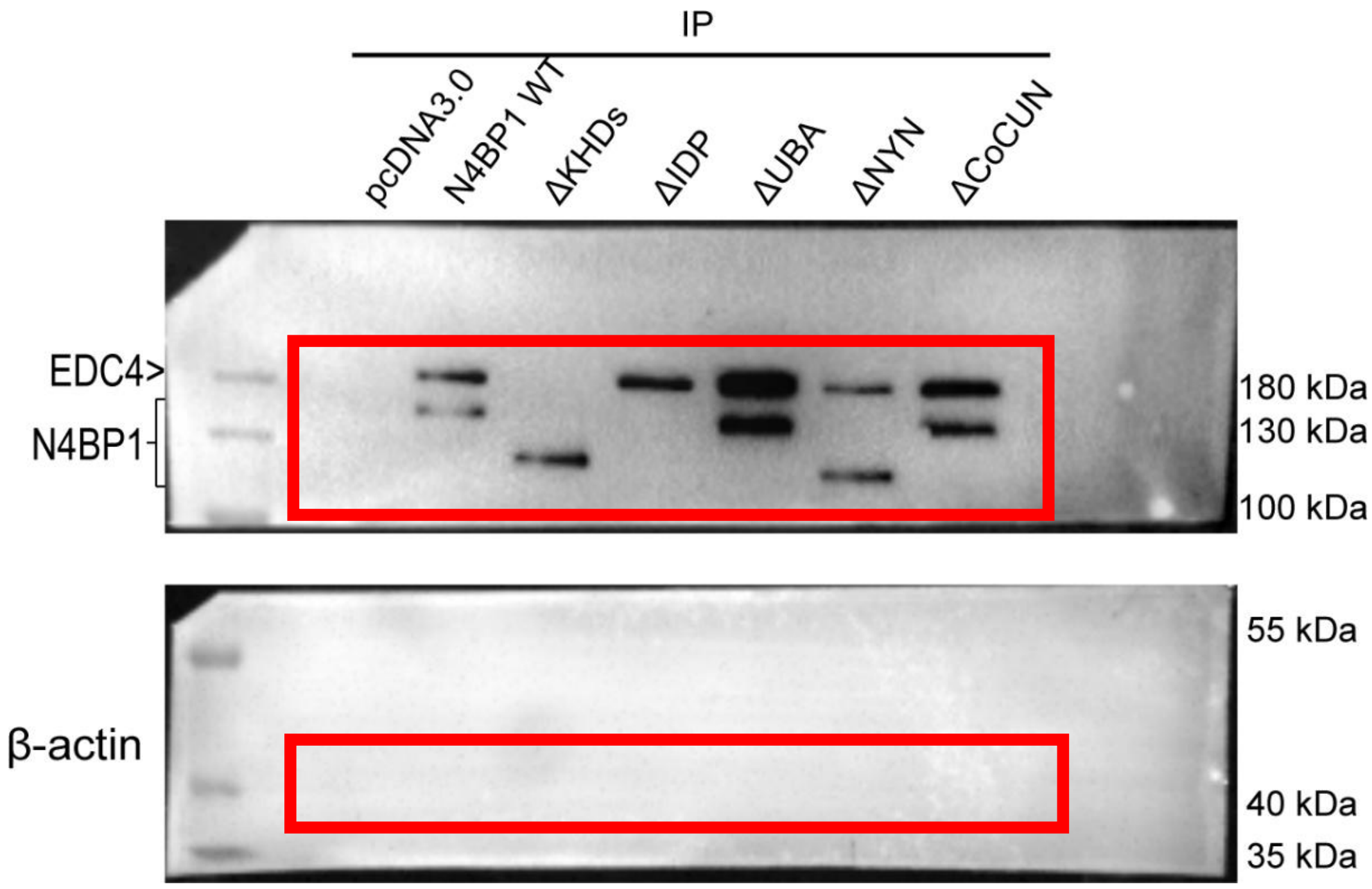

Raw blot images (merged with MW marker) for **Fig. 3D**

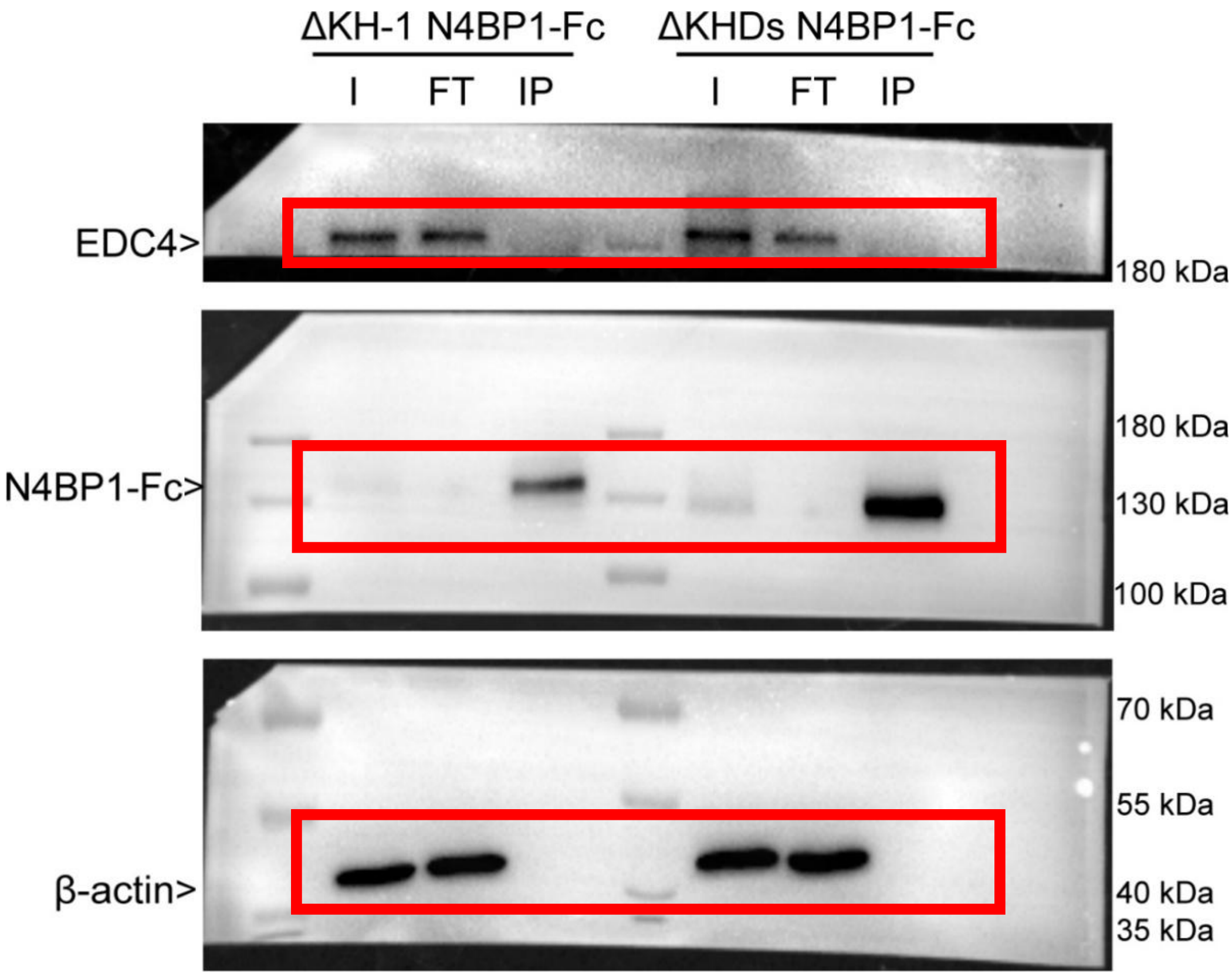

Raw blot images (merged with MW marker) for **Fig. 3G**

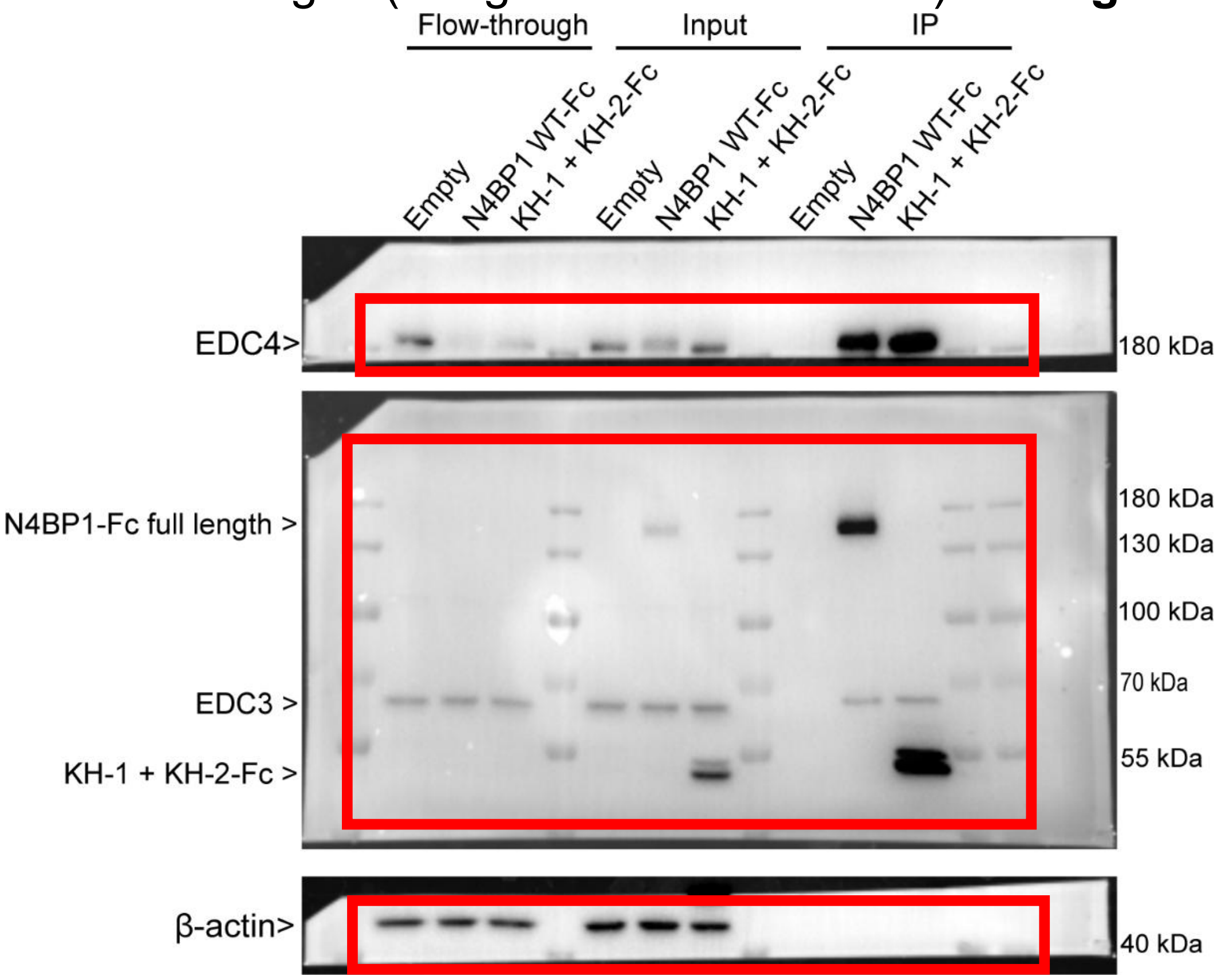

Raw blot images (merged with MW marker) for **Fig. 4D**

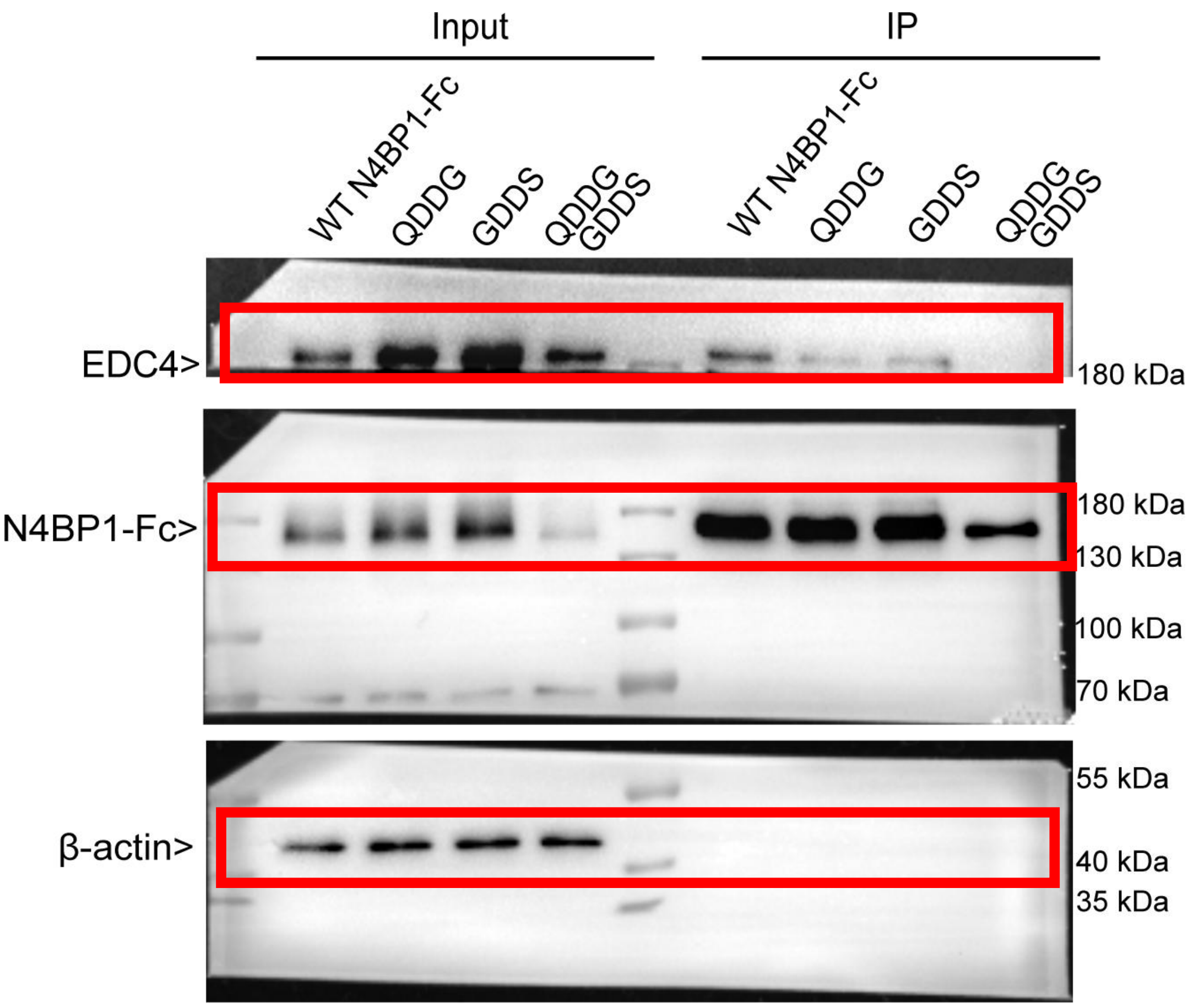

Raw blot images (merged with MW marker) for **Fig. 5A**

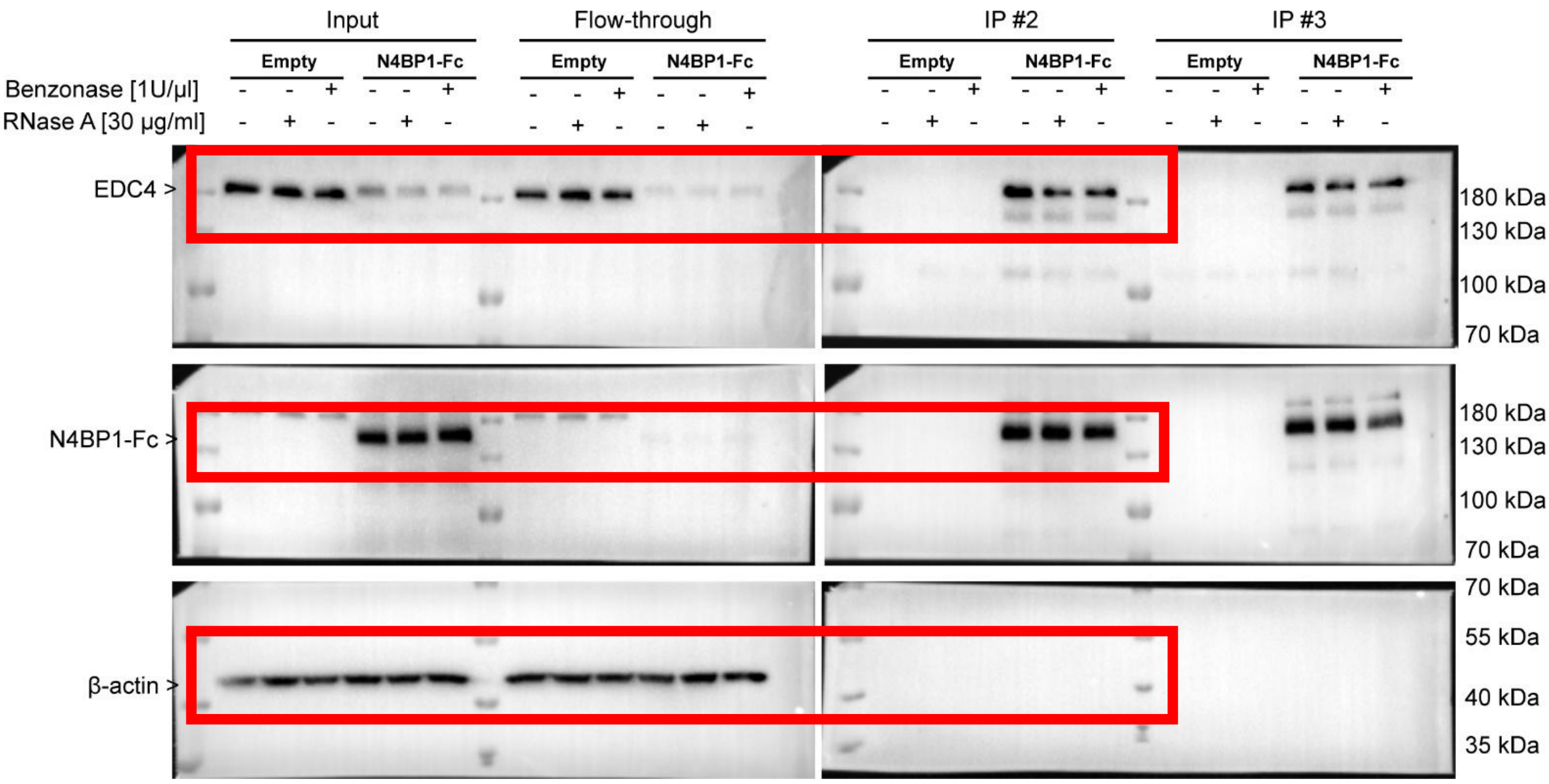

Raw blot images (merged with MW marker) for **Fig. 6D**

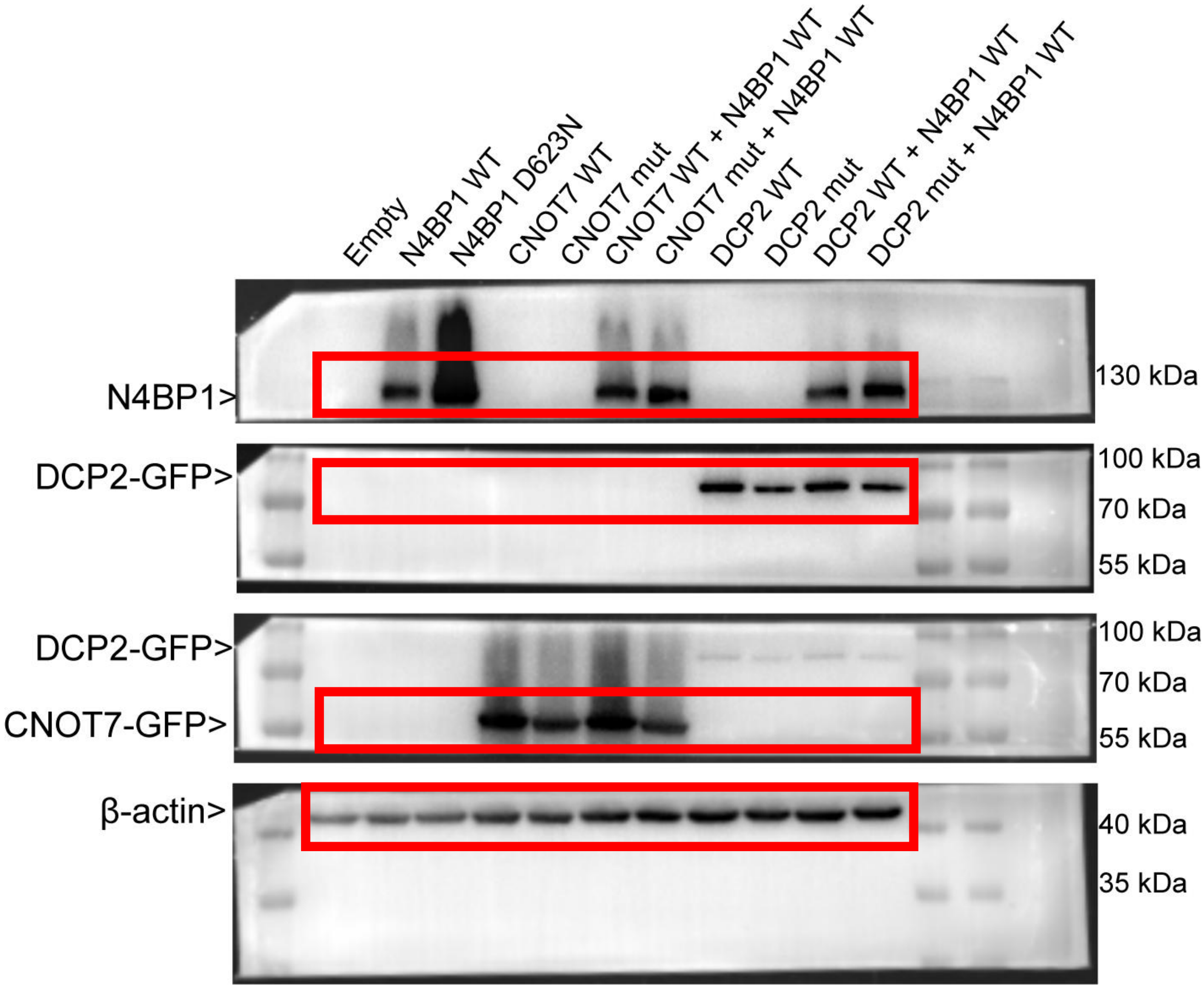

Raw blot images (merged with MW marker) for **Fig. S8B**

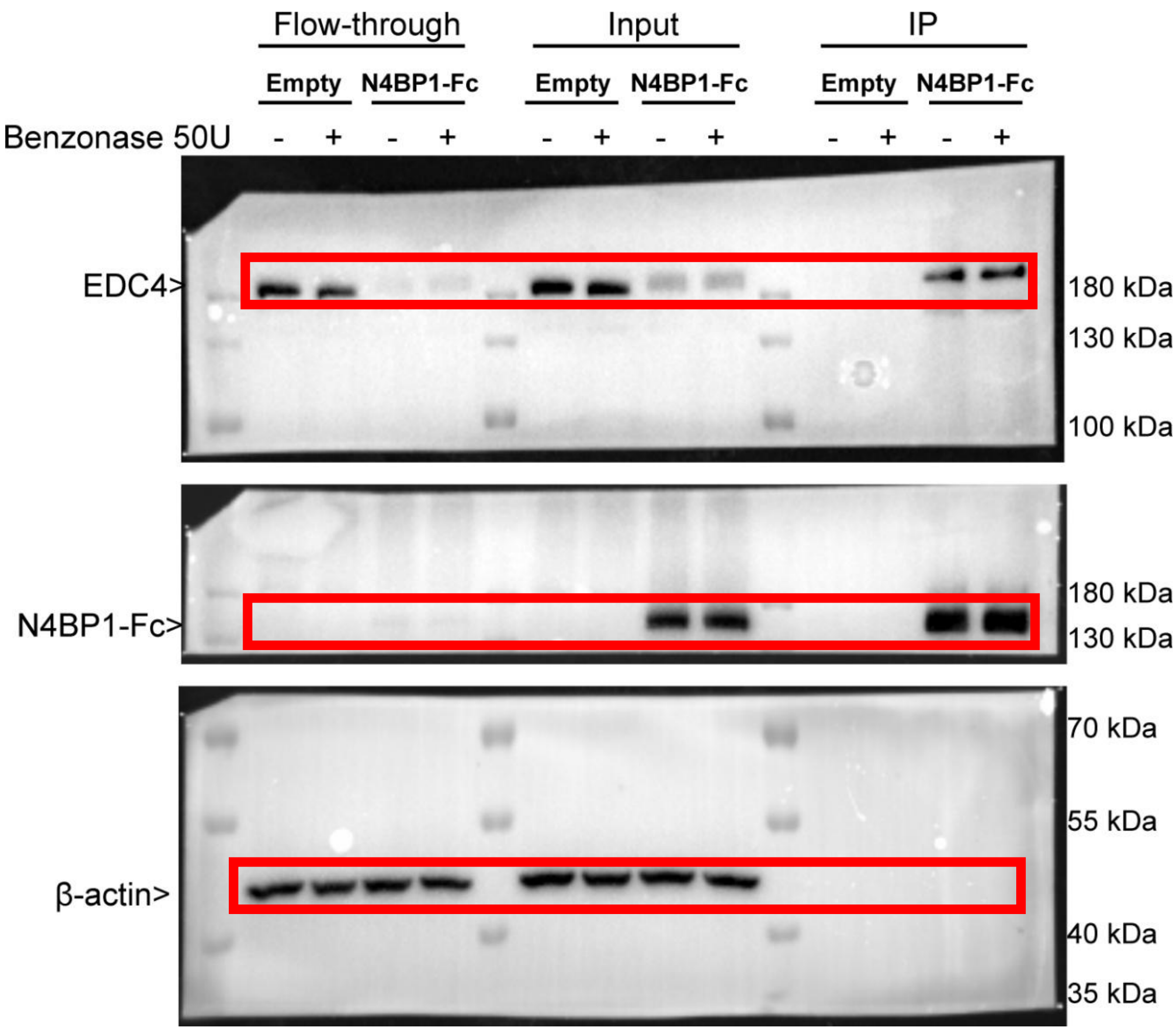

Figure S13. Raw, uncut Western blot membranes of the blots shown in the main manuscript. The corresponding main manuscript figure number for each membrane is indicated above the respective membrane in this figure. Red fields mark the parts included in the main manuscript figures.

Supplementary Table 1. Sequences of primers used to amplify mutations of N4BP1 in PCR and Q-RT-PCR targets and target sequences of used siRNAs.

Primer name:	Primer sequence 5′-3′
ΔKH-1 1-57 Forward	GCGCAGGAGGCGGTGCACAGC
ΔKH-1 1-57 Reverse	CATGGCGGGCGCGGCCTC
ΔKHDs 1-191 Forward	GGTGAGGAGAATCTCTTTGAAACAGG
ΔKHDs 1-191 Reverse	CATGGCGGGCGCGG
ΔIDP 192-612 Forward	GGGAGAACGGATTTGAAACACATTGTTATAGATGG
ΔIDP 192-612 Reverse	TTGTGTGAGTGTCAAAAGTTCTTTTTTCAAGG

ΔUBA 344-392 Forward	GAAGACAGAGAATTTTCAGCTG
ΔUBA 344-392 Reverse	CTCAGTAGTTTCTTTTATATCTGGAC
ΔNYN 616-896 Forward	GGATCCGACAAAACTCACACATGC
ΔNYN 616-896 Reverse	ATCCGTTCTCCCTGGTTCA
ΔCoCUN 849-896 Forward	GGATCCGACAAAACTCACACATGC
ΔCoCUN 849-896 Reverse	CTGAGCTGGCATGGGCAGG
QDDG Forward	GACGACGGCCGTATCGAGGGCCTG
QDDG Reverse	CTGCTCCAGCAGCTCCG
GDDS Forward	GACGACAGCCTGTTTCTGAAAAGCTTG
GDDS Reverse	CCCAACAAAAATGCAGTGCATG
N4BP1 D623N Forward	TTGTTATAAATGGGAGCAATGTTGC
N4BP1 D623N Reverse	TGTGTTTCAAATCCGTTCT
HIV-1 *pol* Forward	ACATGGGAAGCATGGTGGACA
HIV-1 *pol* Reverse	TTTCCCTATTGGCTGCCCCA
HIV-1 *Tat/rev* Forward	ATGGCAGGAAGAAGCGGAG
HIV-1 *Tat/rev* Reverse	ATTCCTTCGGGCCTGTCG
28s rRNA Forward	CACCCACTAATAGGGAACGTG
28s rRNA Reverse	CTGACTTAGAGGCGTTCAGTC
N4BP1 Forward	CAGTGGAGAACAAGGCGTGA
N4BP1 Reverse	GCCACCAGTTTTGTCCGCTA
FREM2 Forward	TGGACAAAGGCAACCACAGC
FREM2 Reverse	CGTTAACTTGTTTCCACCACCATCT
NOTCH3 Forward	TGGCAGTAGAGGGCATGGTG
NOTCH3 Reverse	GGGGGTCTCCTCCTTGCTAT
ETV1 Forward	TACATGGTCACCAATAGTCAGCG
ETV1 Reverse	CTTCTGCAAGCCATGTTTCCTG
KH-1/2only Forward	GGAGGAGGATCCGACAAAACTCACACATGC
ΔKH-1 1-76 Forward	CTAGAAGAAAGAGAATGTTACCCCAAGG
KH-1only Reverse	AGGTTCACAGATTCCTTTAATATATTCCTTGG
KH-2only Reverse	TTTATTTTCAAAGAGCTTTACAAATTGTTGAATGTGACTCC
siRNA:	siRNA target sequence:
siGENOME Non-targeting siRNA pool D-001206-13-05 (siControl)	UAGCGACUAAACACAUCAA
	UAAGGCUAUGAAGAGAUAC
	AUGUAUUGGCCUGUAUUAG
	AUGAACGUGAAUUGCUCAA

siGENOME Human *EDC4* siRNA SMART pool D-016635 (siEDC4)	GGACCAUGCCACCCAUUAA
	GAUGGAGAUCGGCAUAAUA
	UGACCAGAACCGAGAGUUA
	GCCCACAGCACCUGACAUU
