# Supplementary material for: N4BP1 uses tandem KH domains to associate with EDC4 and mRNA decapping factors in P-bodies
Source: Commun Biol. 2026 Sep 17;9:1215. doi: 10.1038/s42003-026-10938-x (PMC13586276; doi:10.1038/s42003-026-10938-x)
Supplement: Supplementary file 3 [file 42003_2026_10938_MOESM3_ESM.pdf]

Description of Additional Supplementary Files

Supplementary data 1 contains list of proteins identified in IP-MS experiments.

Supplementary data 2 contains Gene Ontology results on IP-MS data.

Supplementary data 3 contains details on Manders' Overlap Coefficient calculations.

Supplementary data 4 contains gene counts obtained upon RNAseq analysis and Gene Ontology results on RNAseq data.
